# 2021 ISHNE/HRS/EHRA/APHRS collaborative statement on mHealth in Arrhythmia Management: Digital Medical Tools for Heart Rhythm Professionals: From the International Society for Holter and Noninvasive Electrocardiology/Heart Rhythm Society/European Heart Rhythm Association/Asia Pacific Heart Rhythm Society

**DOI:** 10.1002/joa3.12461

**Published:** 2021-01-29

**Authors:** Niraj Varma, Iwona Cygankiewicz, Mintu Turakhia, Hein Heidbuchel, Yufeng Hu, Lin Yee Chen, Jean‐Philippe Couderc, Edmond M. Cronin, Jerry D. Estep, Lars Grieten, Deirdre A. Lane, Reena Mehra, Alex Page, Rod Passman, Jonathan Piccini, Ewa Piotrowicz, Ryszard Piotrowicz, Pyotr G. Platonov, Antonio Luiz Ribeiro, Robert E. Rich, Andrea M. Russo, David Slotwiner, Jonathan S. Steinberg, Emma Svennberg

**Affiliations:** ^1^ Cleveland Clinic OH USA; ^2^ Medical University of Lodz Lodz Poland; ^3^ Stanford University Palo Alto CA USA; ^4^ Antwerp University and University Hospital Antwerp Belgium; ^5^ Taipei Veterans General Hospital Taipei Taiwan; ^6^ University of Minnesota Minneapolis MN USA; ^7^ University of Rochester Rochester NY USA; ^8^ Temple University Philadelphia PA USA; ^9^ Hasselt University Hasselt Belgium; ^10^ University of Liverpool Liverpool UK; ^11^ Northwestern University Feinberg School of Medicine Chicago IL USA; ^12^ Duke University Durham NC USA; ^13^ National Institute of Cardiology Warsaw Poland; ^14^ Lund University Lund Sweden; ^15^ Faculdade de Medicina Centro de Telessaúde Hospital das Clínicas and Departamento de Clínica Médica Universidade Federal de Minas Gerais Belo Horizonte Brazil; ^16^ Cooper Medical School of Rowan University Camden NJ USA; ^17^ Cardiology Division NewYork‐Presbyterian Queens and School of Health Policy and Research Weill Cornell Medicine New York NY USA; ^18^ Karolinska University Hospital Stockholm Sweden

**Keywords:** arrhythmias, digital medicine, heart rhythm, atrial fibrillation, comorbidities, mHealth

## Abstract

This collaborative statement from the International Society for Holter and Noninvasive Electrocardiology/Heart Rhythm Society/European Heart Rhythm Association/Asia Pacific Heart Rhythm Society describes the current status of mobile health (“mHealth”) technologies in arrhythmia management. The range of digital medical tools and heart rhythm disorders that they may be applied to and clinical decisions that may be enabled are discussed. The facilitation of comorbidity and lifestyle management (increasingly recognized to play a role in heart rhythm disorders) and patient self‐management are novel aspects of mHealth. The promises of predictive analytics but also operational challenges in embedding mHealth into routine clinical care are explored.

## Graphical abstract

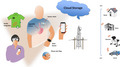

**Abbreviations**
AI: artificial intelligence
ACC: American College of Cardiology
ACS: acute coronary syndrome
AED: automated external defibrillator
AF: atrial fibrillation
AHA: American Heart Association
AHRE: atrial high‐rate episode
APHRS: Asia Pacific Heart Rhythm Society
BP: blood pressure
CIED: cardiovascular implantable electronic device
CPR: cardiopulmonary resuscitation
EHRA: European Heart Rhythm Association
EMR: electronic medical record
ESUS: embolic stroke of unknown source
FDA (U.S.): Food and Drug Administration
GPS: global positioning system
HCP: healthcare professional
HF: heart failure
HR: heart rate
HRS: Heart Rhythm Society
ICD: implantable cardioverter‐defibrillator
ILR: implantable loop recorder
ISHNE: International Society for Holter and Noninvasive Electrocardiology
JITAI: just‐in‐time adaptive intervention
MCT: mobile cardiac telemetry
OAC: oral anticoagulant
PAC: premature atrial complex
PPG: photoplethysmography
PVC: premature ventricular complexes
SCA: sudden cardiac arrest
TADA: Technology Assissted Dietary Assessment
VT: ventricular tachycardia

**Table:** 

1. INTRODUCTION	3
Document scope and rationale	3
2. mHEALTH TECHNOLOGIES	5
2.1. Ambulatory ECG monitoring	6
2.2. New mHealth‐based modalities for arrhythmia monitoring	7
2.2.1 ECG‐based	7
2.2.1.1. Handheld devices	8
2.2.1.2. Wearable patches	8
2.2.1.3. Biotextiles	9
2.2.1.4. Smartphone and smartwatch‐based devices	9
2.2.2 Non‐ECG‐based	10
2.2.2.1. Photoplethysmography	10
2.2.2.2. Oscillometry	10
2.2.2.3. Mechanocardiography	10
2.2.2.4. Contactless video plethysmography	11
2.2.2.5. Smart speakers	11
3. mHEALTH APPLICATIONS FOR ARRHYTHMIAS	14
3.1. Atrial fibrillation	14
3.1.1. Undiagnosed atrial fibrillation identification	14
3.1.2. Targeted identification in high‐risk individuals	17
3.1.3. Diagnostics in people with established atrial fibrillation	18
3.1.4. Atrial fibrillation therapy	18
3.2. Sudden cardiac death	19
4. COMORBIDITIES	24
4.1. Ischemic heart disease	24
4.2. Heart failure	25
4.2.1. Mobile technologies for managing heart failure	26
4.2.2. Hybrid telerehabilitation in patients with heart failure	27
4.3. Diabetes	27
4.4. Hypertension	27
4.5. Disorders including sleep apnea	28
4.6. Lifestyle	28
4.6.1. Physical activity	28
4.6.2. Diet	29
5. PATIENT SELF‐MANAGEMENT—INTEGRATED CHRONIC CARE	34
5.1. Patient engagement	34
5.2. Behavioral modification	35
5.3. Patients as part of a community	35
5.4. Maintaining patient engagement	35
5.5. Digital divide	36
6. CLINICAL TRIALS	38
6.1 Screening	39
6.1.1 The Apple heart study	39
6.1.2 The Huwaei heart study	39
6.1.3 Fitbit study	39
6.2 Point of care	39
6.3 Questions	40
6.3.1 Generalizability	40
6.3.2 Adherence	40
6.3.3 Outcomes	40
7. OPERATIONAL CHALLENGES	41
7.1. Healthcare system—eHealth monitoring and hospital ecosystem	41
7.2. Cybersecurity guidance for mHealth devices	42
7.2.1. Hacking strategies and methods in mHealth technologies	42
7.2.2. Recommendations to the manufacturer	42
7.2.3. Recommendations to clinicians and administrator	43
7.2.4. Recommendations to patients	43
7.3. Reimbursement	43
7.4. Regulatory landscape for mHealth devices	44
8. Predictive analytics	45
9. Future directions	47
CONCLUDING REMARKS	50

## Central Figure

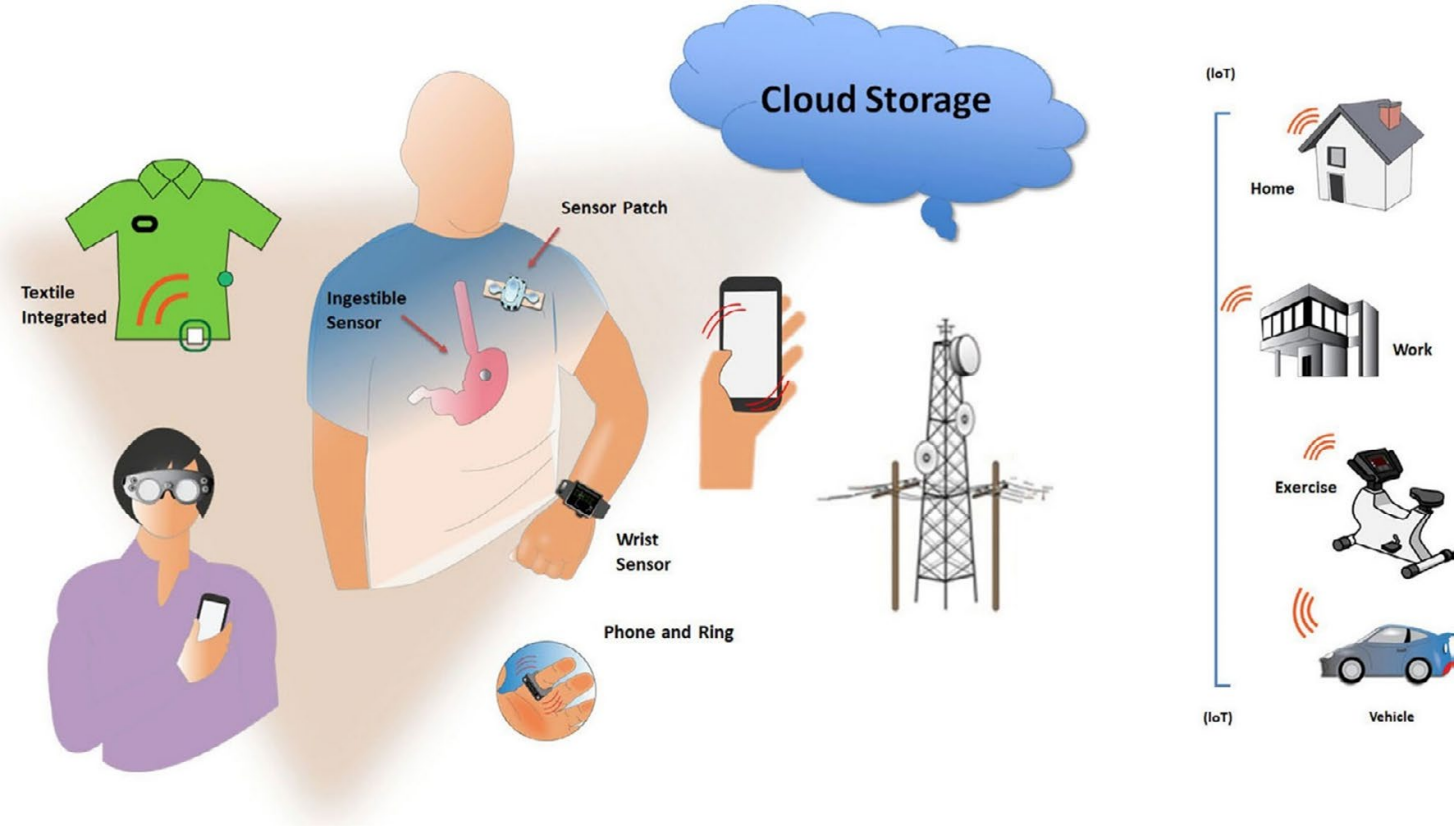

**FIGURE LEGEND** mHealth tools for the individual. Sensors can be embedded in a variety of wearables. (IoT: Internet of things—connects from any location to hospital or cloud; See Table 1).

**TABLE 1:** mHealth‐based modalities for arrhythmia monitoring

	Signal acquisition and visualization	ECG duration	Signal storage and transmission	Indications/Populations tested	Advantages	Limitations
ECG‐based devices						
Handheld	External sensors; Single or multilead ECG on demand; Display in‐screen ECG or screen of PC/laptop/smartphone, after transmission or real‐time ECG analysis available	Intermittent Recording: 10 sec to 2 min	Built‐in memory Bluetooth WiFi	Palpitations AF screening	Easy to use Low cost	Short ECG duration
Wearable patches	Built‐in electrodes Patch attached to the skin	Continuous recording Up to 14 days	Built‐in memory with post hoc analysis, or Bluetooth transmission with real‐time analysis in selected devices	Low‐risk patients with palpitations and syncope; AF screening	Continuous longer‐term ECG recording; Built‐in alarm button High patients’ compliance; Patients can affix at home Water‐resistant	Single‐channel ECG Skin irritation
Biotextiles	Electrodes/sensors embedded into biotextile—vests, belts Single or multichannel	Continuous recording up to 30 days	Built‐in memory Real‐time Bluetooth transmission	Low‐risk patients with palpitations and syncope AF screening	Continuous long‐term recording; Built‐in alarm button; High patients’ acceptance and adherence; Multiparameter evaluation; Can be used as monitoring and treating device (WCD)	Limited Availability Movement artifacts
Smartphone‐based	External sensors attached to mobile phone Single/multilead ECG Real‐time ECG on smartphone’s screen or PC/laptop after transmission	Intermittent recording up to 30 sec Patient activated	Built‐in memory Real‐time or post hoc transmission	Low‐risk patients with palpitations AF screening	Widely available Long‐life possibility of intermittent recording	Intermittent recording
Smartwatch‐based	Built‐in sensors	Intermittent recording Patient activated	Built‐in memory Real‐time or post hoc transmission	Low‐risk patients with palpitations AF screening	Widely available Long‐life possibility of intermittent recording	Intermittent recording Single‐channel
Non‐ECG‐based						
Photoplethysmography (PPG)	HR from changes in reflectance of the tissue blood volume of a skin surface	Intermittent patient activated in smartphones Continuous measurement of HR in smartwatches and wristbands	Built‐in memory Real‐time or post hoc transmission	Low‐risk patients with palpitations AF screening HR measurement during physical activity	Widely available	Irregular heart‐presumed AF
Oscillometry	BP monitors with HR measurement	Intermittent recording during BP measurement	Built‐in memory Post hoc transmission	HR assessment Opportunistic AF screening	Widely available	Irregular heart‐presumed AF
Video recording	Camera from smartphones, TVs	Patient activated Continuous recording in prespecified time frame	Real‐time or post hoc transmission	Low‐risk patients with palpitations AF screening Undiagnosed falls	Can use existing cameras from household goods	Irregular heart‐presumed AF Limited availability

Abbreviations: AF, atrial fibrillation; BP, blood pressure; HR, heart rate; WCD, wearable cardioverter‐defibrillator.

## 1 INTRODUCTION

### 1.1 Document scope and rationale

Digital health is an umbrella term to describe the use of digital information, data, and communication technologies to collect, share, and analyze health information in order to improve patient health, education, and healthcare delivery (https://www.fcc.gov/general/five‐questions‐you‐can‐ask‐your‐doctor‐about‐digital‐health#ab).^1^ This concept encompasses telehealth, electronic health records, implantable device monitoring, wearable sensor data, analytics and artificial intelligence (AI), behavioral health, and personalized medicine. Among these, mobile health—or “mHealth” is a component of digital health, defined by the World Health Organization—as “medical and public health practice supported by mobile devices, such as mobile phones, patient monitoring devices, personal digital assistants (PDAs), and other wireless devices” (https://www.who.int/goe/publications/goe_mhealth_web.pdf;https://apps.who.int/gb/ebwha/pdf_files/WHA71/A71_20‐en.pdf?ua=1).^2^ ^,^ ^3^ Utilization of these devices has proliferated among health‐conscious consumers in recent years and is likely to continue rapid expansion and integration into more formalized medical settings.

mHealth flows intuitively to health professionals in the field of arrhythmia management from experience gained through remote monitoring of cardiovascular implantable electronic devices (CIEDs), such as pacemakers and implantable cardioverter‐defibrillators (ICDs).^4^ A wealth of data garnered from many studies over the last 10‐15 years have confirmed the benefits of remote technology‐assisted follow‐up and established it as standard of care.^5^ ^,^ ^6^ However, results of remote monitoring of CIEDs may not be immediately generalizable to mHealth. For instance, the former is restricted to those with cardiac disease (largely arrhythmias and heart failure (HF)), that is, a group already defined as patients. The care pathways for CIED remote monitoring are also well defined, with billing and reimbursement in place in the United States and many other parts of the world. In comparison, mHealth differs: It is widely available in the form of consumer products that penetrate most sectors of society, including individuals without formal medical diagnoses; it may be applied to a wider group of medical conditions; data can be self‐monitored rather than assessed by healthcare professionals (HCPs); and reimbursement models are not mature. Indeed, some heart rhythm tracking capabilities may be indirectly acquired in products purchased for different goals and then subsequently used for self‐monitoring. Conversely, in the medical space, applications are largely not prescribed by HCPs, often lack validation for disease management use cases, and care pathways remain varied or poorly defined. Nevertheless, if properly implemented, the intersection of these two communities opens up a broad spectrum of opportunities, extending from population screening and surveillance for undiagnosed disease to longitudinal disease management, and importantly, engaging patients in their own cycle of care, allowing much health care to be asynchronous and virtualized. Its value and degree of integration will depend on different healthcare systems in different countries.

mHealth has value only if the acquired information leads to decisions that improve outcome. This requires a clear path of information flow and actionability. Moreover, all stakeholders need to be aware of the logistical chain (so that everyone knows what to expect) and responsibilities clearly defined (possibly including device vendors). Similarly, actions taken based on the monitored information should be transparent to all stakeholders. For example, for a patient who records and transmits an irregular heart rhythm via a wearable device, a designated decision process should be followed to confirm eg whether the rhythm is atrial fibrillation (AF) or not, whether confirmation by another diagnostic test is required, how that is arranged, and finally what therapy should be implemented and in what reasonable time frame? Clearly, there are risks of increasing cost from medical testing and provoking anxiety in consumers—who by virtue of seeking a medical verification become patients. Again, CIED experience sets a precedent. Studies that have shown improved outcome with telemonitoring succeeded when integrated into a clear logistical framework for a specific use case of disease management (e.g., IN‐TIME for remote monitoring in patients receiving cardiac resynchronization therapy, CardioMEMS).^6^, ^7^, ^8^ Replicating this with mHealth creates challenges for healthcare providers and goes far beyond the technological capabilities of the monitoring and transmission equipment. Implementation will require defined aims and fundamental changes to existing workflows and responsibilities. Such changes are always difficult. Apart from the organizational issues required to achieve such changes, reimbursement may drive or hinder such changes in the workplace. Awareness of these factors has been heightened by the SARS‐CoV‐2 pandemic, during which telemedicine solutions have been advocated to reduce patient contact with healthcare providers yet continue healthcare delivery.^9^

In view of the rapid technological development and popularity of wearable and other mobile devices, and the need for analysis and planning of the mHealth infrastructure, ISHNE (International Society for Holter and Noninvasive Electrocardiology), HRS (Heart Rhythm Society), EHRA (European Heart Rhythm Association), and APHRS (Asia Pacific Heart Rhythm Society), recognized the need for this collaborative statement. The aim of this document is to define state‐of‐the‐art mHealth technologies and their application in arrhythmia management and explore future directions for clinical application. As such, the scope of the document encompasses discussion of the different mHealth technologies currently available or in development; the acquisition of health‐related data; the applications of such data, including disease identification and management; clinical trials; the patient perspective; and the issues that must be addressed in the future to permit useful application of mHealth technologies. Addtionally, discussion is extended to mHealth facilitation of those comorbidities increasingly recognized to influence arrhythmia management (e.g., obesity and sleep apnea) that are becoming the responsibility of heart rhythm professionals.^10^

REFERENCES SECTION 11

Turakhia
MP
, 
Desai
SA
, 
Harrington
RA
. The outlook of digital health for cardiovascular medicine: challenges but also extraordinary opportunities. Journal of the American Medical Association Cardiology. 2016;1:743–744.2758027510.1001/jamacardio.2016.26612
World Health Organization
. mHealth New horizons for health through mobile technologies. Switzerland; 2011. Available at: https://www.who.int/goe/publications/goe_mhealth_web.pdf.3
World Health Organization
. mHealth: Use of appropriate digital technologies for public health. Switzerland; 2018. Available at: https://apps.who.int/gb/ebwha/pdf_files/WHA71/A71_20‐en.pdf?ua=1.4

Varma
N
, 
Epstein
AE
, 
Irimpen
A
, 
Schweikert
R
, 
Love
C
. Efficacy and safety of automatic remote monitoring for implantable cardioverter‐defibrillator follow‐up. Circulation. 2010;122:325–332.2062511010.1161/CIRCULATIONAHA.110.9374095

Slotwiner
DJ
, 
Varma
N
, 
Akar
JG
, 
Annas
G
, 
Beardsall
M
, 
Fogel
RI
, … 
Yu
CM
. HRS Expert Consensus Statement on remote interrogation and monitoring for cardiovascular implantable electronic devices. Heart Rhythm. 2015;12(7):e69–e100. 10.1016/j.hrthm.2015.05.008.259811486

Varma
N
, 
Ricci
RP
. Telemedicine and cardiac implants: What is the benefit?
European Heart Journal. 2013;34:1885–1895. 10.1093/eurheartj/ehs388.23211231PMC40512587

Abraham
WT
, 
Adamson
PB
, 
Bourge
RC
, 
Aaron
MF
, 
Costanzo
MR
, 
Stevenson
LW
, … 
Yadav
JS
. Wireless pulmonary artery haemodynamic monitoring in chronic heart failure: A randomised controlled trial. Lancet. 2011;377:658–666. 10.1016/S0140-6736(11)60101-3.213154418

Hindricks
G
, 
Taborsky
M
, 
Glikson
M
, 
Heinrich
U
, 
Schumacher
B
, 
Katz
A
, … 
Søgaard
P
. IN‐TIME study group. Implant‐based multiparameter telemonitoring of patients with heart failure (IN‐TIME): A randomized controlled trial. Lancet. 2014;384:583–590.2513197710.1016/S0140-6736(14)61176-49

Varma
N
, 
Marrouche
NF
, 
Aguinaga
L
, 
Albert
CM
, 
Arbelo
E
, 
Choi
JI
, … 
Varosy
PD
. HRS/EHRA/APHRS/LAHRS/ACC/AHA worldwide practical guidance for telehealth and arrhythmia monitoring during and after a pandemic. Journal of the American College of Cardiology. 2020;76:1363–1374. 10.1016/j.jacc.2020.06.019.32534936PMC728908810

Chung
MK
, 
Eckhardt
LL
, 
Chen
LY
, 
Ahmed
HM
, 
Gopinathannair
R
, 
Joglar
JA
, … 
Trulock
KM
. American Heart Association Electrocardiography and Arrhythmias Committee and Exercise, Cardiac Rehabilitation, and Secondary Prevention Committee of the Council on Clinical Cardiology; Council on Arteriosclerosis, Thrombosis and Vascular Biology; Council on Cardiovascular and Stroke Nursing; and Council on Lifestyle and Cardiometabolic Health. Lifestyle and Risk Factor Modification for Reduction of Atrial Fibrillation: A Scientific Statement from the American Heart Association. Circulation. 2020;141:e750–e772. 10.1161/CIR.0000000000000748.32148086

## 2 mHEALTH TECHNOLOGIES

**FIGURE 1:**
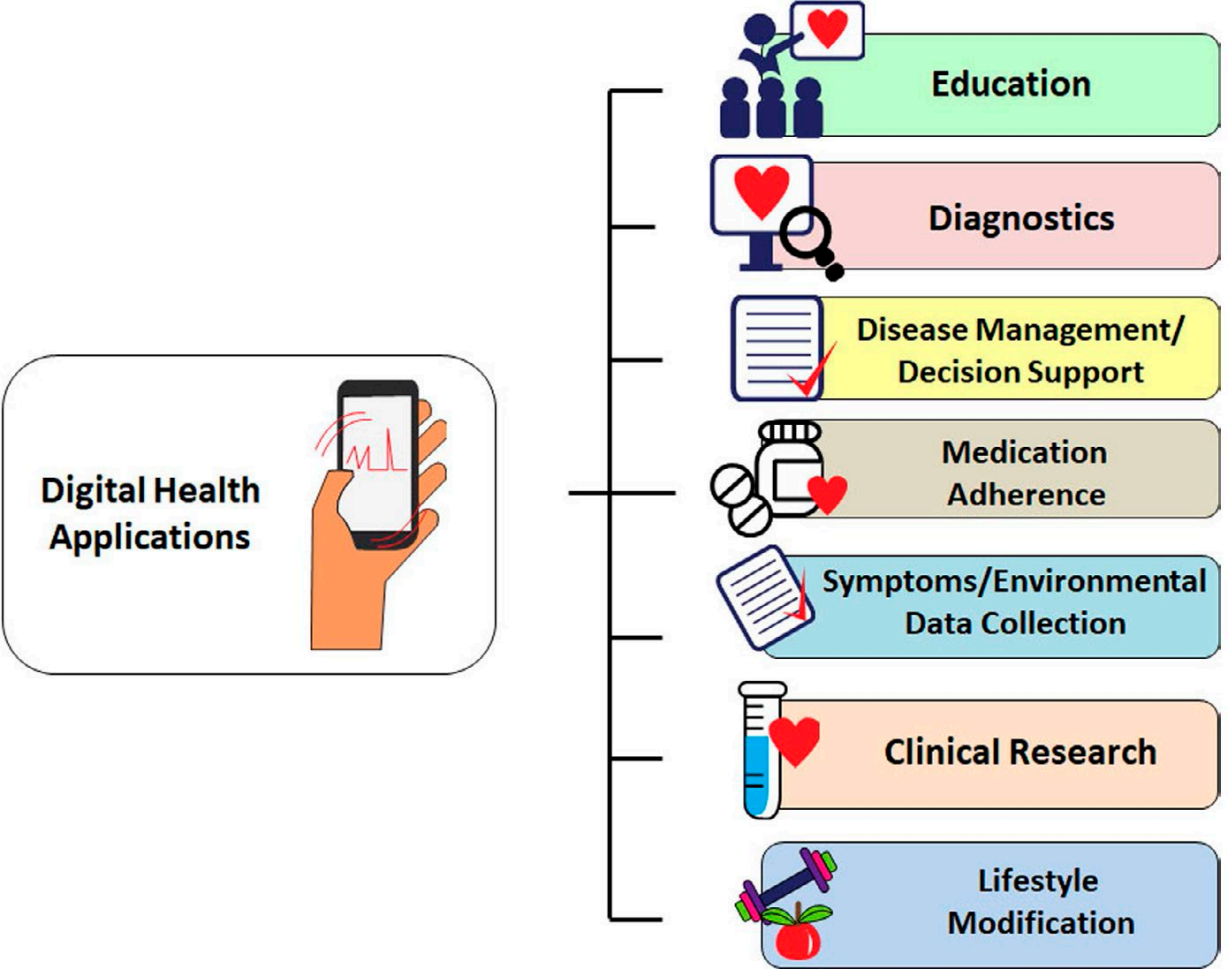
Application of digital health technologies in arrhythmias (Many of these sectors are interconnected).

Dedicated applications and sensors, within or adjunctive to mobile communication devices, enable users to monitor, collect, and share physiologic and health data. Their applications range from diagnostic, decision support, disease management, evaluation of medication adherence, and for educational and clinical research purposes (Figure 1). They synergize naturally with arrhythmia evaluation and extend management to associated comorbidities and lifestyle.

Applications to arrhythmias

- Diagnostic
  - Evaluate patients with symptoms suggestive of arrhythmias
  - Assess patients' response to both pharmacological and invasive treatment of arrhythmias.
- Screening
  - Increasing emphasis on AF.

### 2.1 Ambulatory ECG monitoring

**FIGURE 2:**
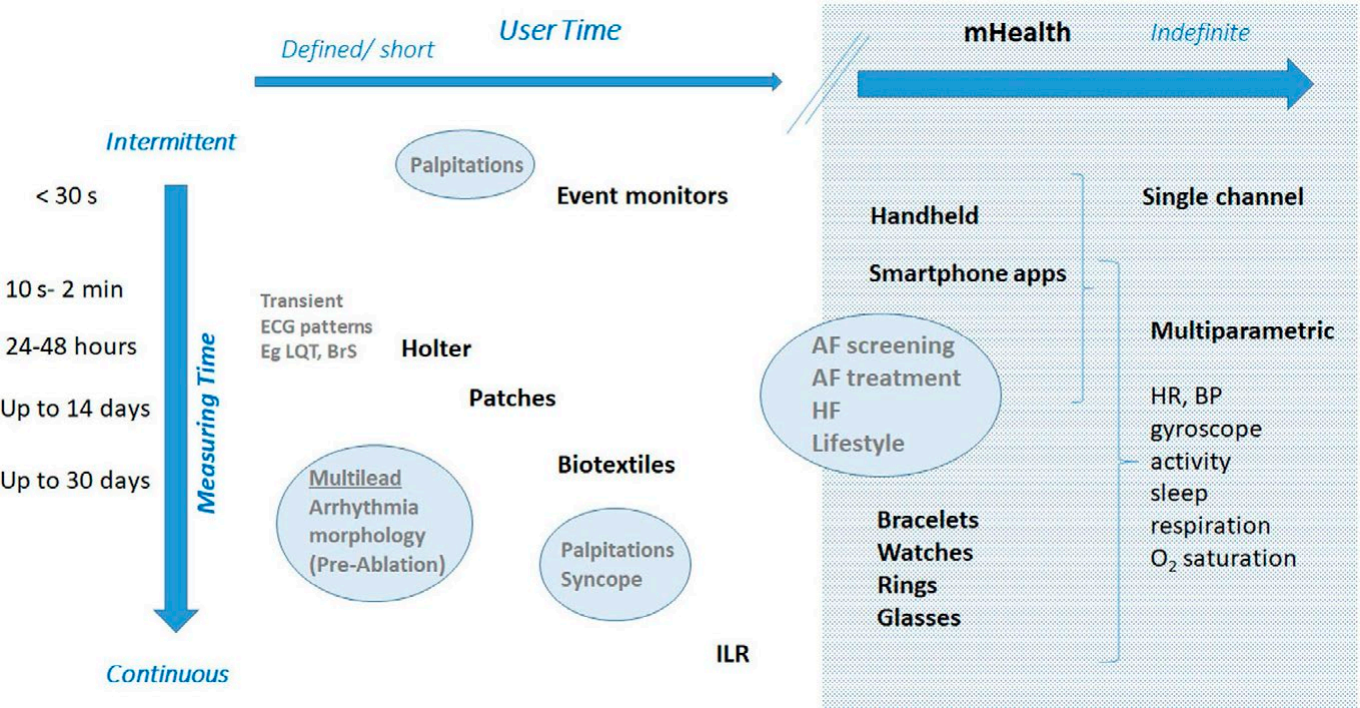
mHealth devices for arrhythmia monitoring according to indications. Traditional wearable monitors are used for defined, short periods of time. Advantages are continuous monitoring and ability to use multiple leads that may be important for arrhythmia differentiation. These have been used historically for evaluation of palpitations, syncope, and defining QRS morphology. mHealth extends monitoring time indefinitely, to be defined by the user, and to the possibility of monitoring other parameters simultaneously with the ECG and linking to machine learning. Typically, mHealth utilizes single‐channel ECG or derived heart rate, and discontinuous monitoring. AF—atrial fibrillation, BP—blood pressure, BrS—Brugada syndrome, HF—heart failure, HR—heart rate, ILR—implantable loop recorder, LQT—long QT.

This is the cornerstone diagnostic method, and the choice of technique and time frame depend on whether symptoms (e.g., palpitations, syncope) are present and how often they occur (Figure 2). Since the XXI century has become the era of the AF epidemic, the emphasis has shifted to screen for asymptomatic patients at high risk of developing AF or in those with cryptogenic stroke, to enable early treatment with the hope of preventing stroke and other serious complications. Novel tools expand the time window in which information can be gathered and overcome existing limitations with traditional methods, that is, intermittent physical examination or ECG for the detection of a largely asymptomatic arrhythmia.

- Conventional ambulatory ECG devices with “continuous” or “intermittent” recording abilities (e.g., Holter, mobile cardiac telemetry; MCT) increase the diagnostic yield for suspected arrhythmias, but limitations such as inadequate duration of monitoring, insufficient sensitivity or specificity for AF detection, cost, and patient discomfort and inconvenience remain important implementation barriers. Further details on these conventional systems are available in a prior expert consensus statement.^21^
- Implantable loop recorders (ILRs) continuously monitor cardiac rhythm, similar to traditional external loop recorders, but only record an ECG shortly before and after activation by either the patient or by an automated algorithm. The total monitoring period is limited only by battery longevity (ca. 2‐5 years). Newer devices have dedicated algorithms resulting in increased interest in their use for AF detection, especially after cryptogenic stroke. Several approved ILR devices are available,^2^, ^3^, ^4^ and several studies have been performed to evaluate the diagnostic accuracy of these devices.^5^, ^6^, ^7^, ^8^, ^9^ Since ILRs are invasive and costly, some functions may shift to mHealth.

### 2.2 New mHealth‐based modalities for arrhythmia monitoring

These can be divided into technologies that:

- Record ECG tracings (single or multilead, in intermittent or continuous format, of various durations).
- use non‐ECG techniques such as pulse photoplethysmography (PPG).

mHealth tools permit indefinite monitoring and widen application to a range of conditions and patient populations. There has been rapid development and integration of diagnostic sensors into consumer devices such as smartwatches, fitness bands, and smartphones. However, validation of their notified data (or underlying algorithms) and mechanisms for professional review (as established for CIEDs and MCTs) are scant, if at all (See Section 7). This is open to risks of not detecting significant events and/or overtreating—for example, false‐positive episodes of AF—if not confirmed by expert physicians.

#### 2.2.1 ECG‐based

Among these, handheld and patch systems have undergone the most extensive validation.

##### 2.2.1.1 Handheld devices

Several stand‐alone handheld devices operate without additional hardware. These devices with two or three ECG electrodes on either side generate short, 30 sec to 1 minute, single or multilead ECG recordings. Some of them display ECG tracings on a monitor. Most of these devices are equipped with dedicated automatic algorithms for detection of arrhythmias and usually focus on AF. Recognition of AF is usually based of the analysis of RR interval irregularity. The devices can store ECG tracings, which can be uploaded to a computer for review and are usually available for physicians via web‐based platforms. Studies across diverse populations have documented the diagnostic accuracy of handheld devices in detection of AF by short‐term rhythm monitoring (Table 2).^10^, ^11^, ^12^, ^13^, ^14^, ^15^, ^16^, ^17^, ^18^

**TABLE 2:** Exemplary validation studies for various mHealth technologies

	Device	Author	n	Setting	Comparator	Sensitivity (%)	Specificity (%)	Requires ECG confirmation
	Pulse palpation	Cooke et al. ^11^	2385	Meta‐analysis	12‐lead ECG	94	72	+
Handheld devices	Zenicor	Doliwa et al. ^12^	100	Outpatient cardiology clinic	12‐lead ECG interpreted by cardiologist	96	92	
	MyDiagnostick	Tieleman et al. ^13^	192	Outpatient cardiology clinic	12‐lead ECG interpreted by cardiologist	100	96	
	Omron HCG‐801	Kearley et al. ^14^	999	Primary care practices	12‐lead ECG interpreted by cardiologist	94.4	94.6	
	Merlin ECG event recorders	Kearley et al. ^4^	999	Primary care practices	12‐lead ECG interpreted by cardiologist	93.9	90.1	
Smartphone ECG device	AliveCor Kardia Mobile	Lau et al. ^15^	204	Recruited patients	12‐lead ECG interpreted by cardiologist	98	97	
Smartphone device PPG	CardioRhythm iPhone	Chan et al. ^16^	1013	Primary care clinic	Single‐lead AliveCor ECG	93	98	+
	PULSE‐SMART App	McManus et al. ^17^	219	Patients undergoing cardioversion	12‐lead ECG or 3‐channel telemetry	97	94	+
	FibriCheck App	Proesmans et al. ^18^	223	Primary care practices	12‐lead ECG	95	97	+
Smartwatch ECG	KardiaBand automated algorithm	Bumgarner et al. ^19^	112	Patients undergoing cardioversion	12‐lead ECG	93	84	
Blood pressure device	Microlife	Wiesel et al. ^20^	405	Cardiology outpatients	12‐lead ECG	95, 97 for one or 3 measurements, respectively	86, 89 for one or 3 measurements, respectively	+

REFERENCES TABLE 21

Cooke
G
, 
Doust
J
, 
Sanders
S
. Is pulse palpation helpful in detecting atrial fibrillation? A systematic review. The Journal of Family Practice. 2006;55:130–134.164517802

Doliwa
PS
, 
Frykman
V
, 
Rosenqvist
M
. Short‐term ECG for out of hospital detection of silent atrial fibrillation episodes. Scandinavian Cardiovascular Journal. 2009;43:163–168. 10.1080/14017430802593435.190969773

Tieleman
RG
, 
Plantinga
Y
, 
Rinkes
D
, 
Bartels
GL
, 
Posma
JL
, 
Cator
R
, …
Houben
RP
. Validation and clinical use of a novel diagnostic device for screening of atrial fibrillation. Europace. 2014;16:1291–1295. 10.1093/europace/euu057.24825766PMC41496084

Kearley
K
, 
Selwood
M
, 
Van den Bruel
A
, 
Thompson
M
, 
Mant
D
, 
Hobbs
FR
, 
Fitzmaurice
D
, 
Heneghan
C
. Triage tests for identifying atrial fibrillation in primary care: A diagnostic accuracy study comparing single‐lead ECG and modified BP monitors. BMJ Open. 2014;4:e004565.10.1136/bmjopen-2013-004565PMC4025411247932505

Lau
JK
, 
Lowres
N
, 
Neubeck
L
, 
Brieger
DB
, 
Sy
RW
, 
Galloway
CD
, 
Albert
DE
, 
Freedman
SB
. iPhone ECG application for community screening to detect silent atrial fibrillation: A novel technology to prevent stroke. International Journal of Cardiology. 2013;165:193–194.2346524910.1016/j.ijcard.2013.01.2206

Chan
PH
, 
Wong
CK
, 
Poh
YC
, 
Pun
L
, 
Leung
WW
, 
Wong
YF
, …
Siu
CW
. Diagnostic performance of a smartphone‐based photoplethysmographic application for atrial fibrillation screening in a primary care setting. Journal of the American Heart Association. 2016;5:e003428. 10.1161/JAHA.116.003428.27444506PMC50153797

McManus
DD
, 
Chong
JW
, 
Soni
A
, 
Saczynski
JS
, 
Esa
N
, 
Napolitano
C
, 
Chon
KH
. PULSE‐SMART: Pulse‐based arrhythmia discrimination using a novel smartphone application. Journal of Cardiovascular Electrophysiology. 2016;27:51–57. 10.1111/jce.12842.26391728PMC47683108

Proesmans
T
, 
Mortelmans
C
, 
Van Haelst
R
, 
Verbrugge
F
, 
Vandervoort
P
, 
Vaes
B
. Mobile phone‐based use of the photoplethysmography technique to detect atrial fibrillation in primary care: Diagnostic accuracy study of the fibricheck app. JMIR Mhealth and Uhealth. 2019;7:e12284.3091665610.2196/12284PMC64568259

Bumgarner
JM
, 
Lambert
CT
, 
Hussein
AA
, 
Cantillon
DJ
, 
Baranowski
B
, 
Wolski
K
, …
Tarakji
KG
. Smartwatch algorithm for automated detection of atrial fibrillation. Journal of the American College of Cardiology. 2018;71:2381–2388. 10.1016/j.jacc.2018.03.003.2953506510

Wiesel
J
, 
Fitzig
L
, 
Herschman
Y
, 
Messineo
FC
. Detection of atrial fibrillation using a modified microlife blood pressure monitor. American Journal of Hypertension. 2009;22:848–852.1947879310.1038/ajh.2009.98

##### 2.2.1.2 Wearable patches

Traditional cable/wire‐based devices increasingly have been displaced by solutions with electrodes embedded in adhesive patches. Commercially available patches can be worn up to 14 days.^39^ ^,^ ^40^ Unlike adhesive electrodes for lead‐based systems, the water‐resistant patches are not removed during the monitoring period leading to greater wear time, more analyzable data, and no lead reversal errors. The cutaneous patch monitors are typically single‐use and continuously or intermittently record single‐lead electrocardiography. Most have an integrated button to mark the timing of symptoms on the recorded rhythm trace. After the monitoring period, the device is returned to the manufacturer for data extraction, analysis by a proprietary algorithm, and further secondary analysis of potential arrhythmias by medical technicians. A diagnostic report is sent to the treating physician. This process may be associated with delays of several weeks.

Although such patches only record a single‐lead ECG, a high agreement (P < 0.001) has been demonstrated compared to multilead Holter monitors for identifying AF events and estimating AF burden.^3^ ^,^ ^41^ As the patch has no external leads, it is perceived to be more comfortable to wear compared to conventional Holter monitors, with 94% of the patients preferring the patch over the Holter.^39^ In addition to the validation studies, the feasibility of two‐week continuous monitoring to identify AF in an at‐risk patient population has been examined by Turakhia et al.^42^. It has also been used successfully to determine the prevalence of subclinical AF in the general population.^43^

Newer patch‐based systems add near‐real‐time analytics and by transmitting data continuously to the cloud. This may facilitate more rapid data collection and diagnosis. Multiparametric monitoring may be enabled with a patch worn for up to 3 months.^44^

##### 2.2.1.3 Biotextiles

Textile‐based systems for ECG monitoring were initially designed to ensure patients' comfort during daily activities and address the needs of active patients. These vests and elastic bands adapt easily to patients' movements that is particularly important for those performing physical activities that might be limited by the presence of wires. These biomedical devices capture the electrocardiographic signal via electrodes integrated into the garment that enables noninvasive acquisition of ECG signal up to 30 days. Single/multilead selection (up to full 12‐leads) and event activation are available. ECG signals can be stored in memory cards and analyzed afterward as well as transmitted in real time via Bluetooth to a smartphone (and from there to a cloud‐based platform), along with other signals including accelerometer and global positioning system (GPS). Other than ECG, some devices provide data on activity intensity, respiratory function, and sleep quality. Automatic analysis with manual verification is possible. Several systems for ECG monitoring based on electrodes incorporated into garments have been introduced into market. Some of them acquire signal from chest belts. Maintaining power presents a challenge. These systems have been tested in athletes, in patients with cryptogenic stroke, and in those with pacemaker‐detected AHRE.^25^, ^26^, ^27^, ^28^, ^29^

The wearable cardioverter‐defibrillator transmits 2‐channel ECG data to an online patient management database allowing for remote monitoring of high‐risk patients. Recent incorporation of heart sound evaluation that may predict HF decompensation will be tested in a prospective trial (*HEARIT‐Reg trial ClinicalTrials.gov Identifier: NCT03203629*).

##### 2.2.1.4 Smartphone and smartwatch‐based devices

**FIGURE 3:**
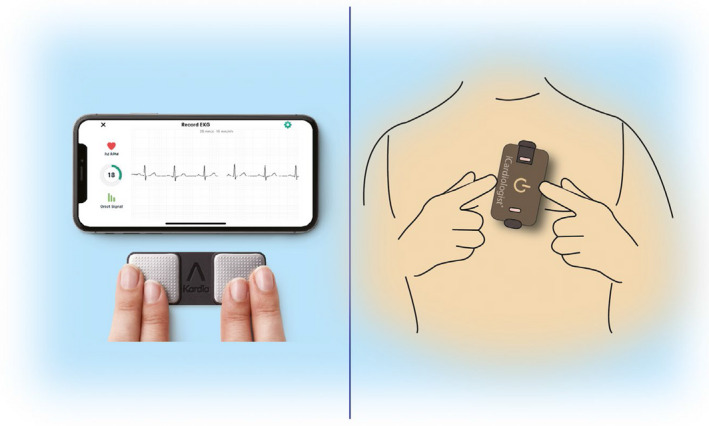
ECG mobile applications. Left—fingertip recordings; Right—card pressed to the chest.

More recently, nonwearable solutions coupled with the smartphone have emerged. These devices (Table 2 and Varma et al.^50^) allow the user to perform a “spot check” single‐lead ECG strip, usually of up to 30 seconds or longer by placing a finger of each hand on the two electrodes, usually located on the phone case or external card (Figure 3). The ECG electrical signal is transmitted wirelessly to a smartphone with an integrated interpretation app. The tracings can be reviewed on the smartphone, electronically stored, or transmitted for review by the user’s provider if desired. These have been directed largely to AF.

**FIGURE 4:**
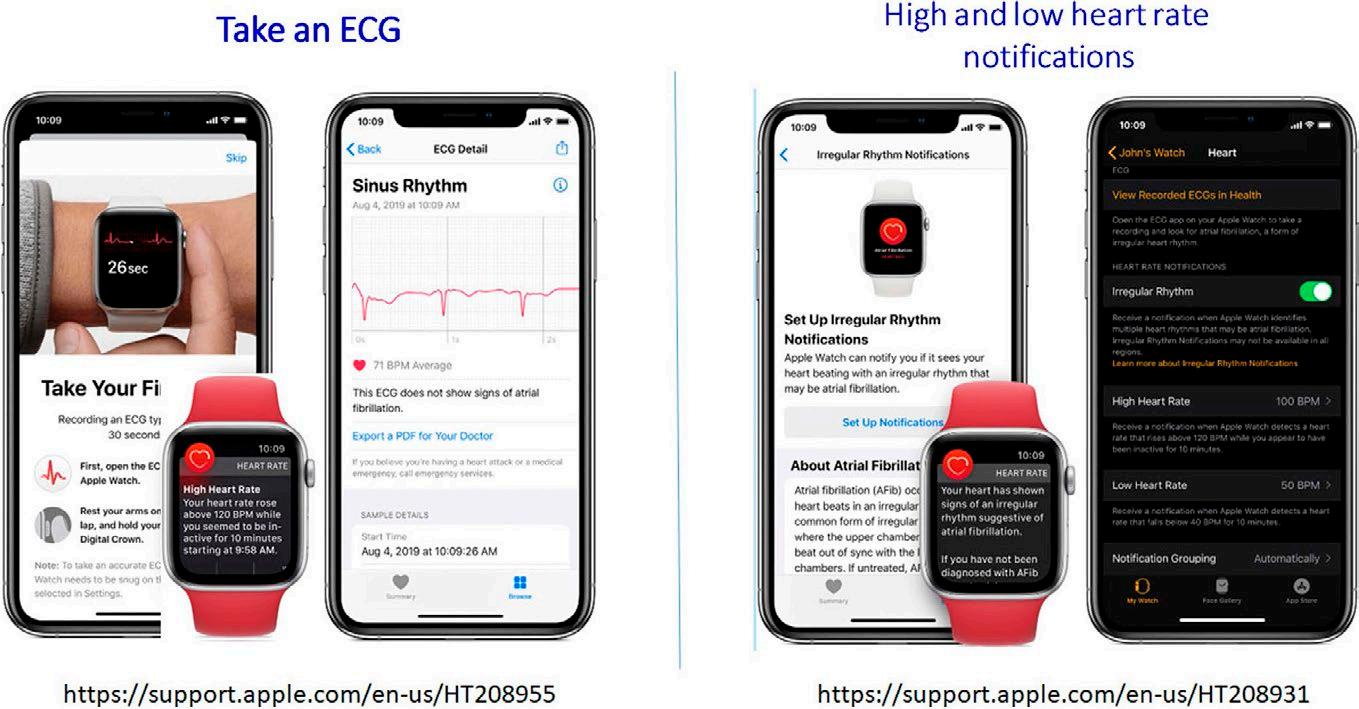
Apple Watch.

Automated algorithms can label the recording as “Possible AF” on the basis of criteria for the presence and absence of a P wave and the irregularity of the RR interval; “Normal” or “Sinus Rhythm” and “Unreadable” when the detector indicates there was too much interference for an adequate recording, whether from too much movement, or poor contact between the electrodes and the patient’s skin. Several versions of the AliveCor’s automated algorithms have been evaluated,^6^, ^31^, ^32^, ^33^, ^34^ and the device has been tested as a screening tool in at‐risk populations.^32^, ^35^ In Apple watch, the algorithm is effective when the heart rate is between 50 and 150 bpm, there are no or very few abnormal beats, and the shape, timing, and duration of each beat is considered normal for the patient (Figure 4).

Sensitivity and specificity depend on the software (which can be calibrated to higher sensitivity or higher specificity), the population studied (e.g., elderly have more tremor and/or difficulty in holding the device leading to more unreadable tracings), and the prevalence of AF in the population. It indicates that use of such device always requires proper evaluation for every intended use case. There is also an accessory band for a smartwatch to allow ECG recording. The single‐lead ECG with automatic AF detection is recorded by touching the band’s integrated sensors that transmit data to a watch application. Recently, a new 6‐lead case has been developed, allowing for 30 second recording of all 6 limb leads by touching each of the three electrodes. Also the QT interval may be derived from this (https://cardiacrhythmnews.com/kardiamobile‐6l‐can‐be‐used‐to‐measure‐qt‐duration‐in‐covid‐19‐patients/).^36^, ^37^ Information is limited; however, on how parameters such as QTc measured on a single (or limited number) lead ECGs can reliably substitute for 12‐lead ECG information. In one study, QT was underestimated by smartphone single‐lead ECG.^58^ Preliminary data indicate ability for ST monitoring for ischemia (Figure 3, Section 4.1).

Such devices may be used by clinicians as a point‐of‐care device to obtain an interpretable rhythm strip in place of a 12‐lead ECG. In addition, patients may use these devices for ad hoc or routine evaluation of their rhythm in a home environment. The ECG data can be instantaneously transmitted for automated interpretation with the ability of the consumer to request a physician overread for a surcharge.

- *Limitations*
  - Single‐lead devices, particularly when used by an active person who may not be recumbent, relaxed, or still, may lead to substantial electrical or motion artifact. Noise‐free tracing may be more difficult for older patients or those with physical limitations (tremor, stroke, etc).
  - Although the interpretation algorithms typically have received regulatory oversight, these algorithms can frequently misclassify rhythms, calling sinus rhythm AF and vice versa, which could lead to potential harm without confirmation by a clinician. For example, in a recent study of a consumer ECG device to detect AF, a third of ECGs were unclassifiable by the device but could be classified by experts.^19^ Therefore, some devices have limitations placed on them for diagnostic assessment. For example, the Apple Watch is unable to assess the ECG for AF if the heart rate is above 150 or below 50 bpm (https://www.apple.com/healthcare/docs/site/Apple_Watch_Arrhythmia_Detection.pdf) and is cleared by the U.S. Food and Drug Administration (FDA) only for use in persons without a diagnosis of AF (Figure 4) (https://support.apple.com/en‐us/HT208931, accessed January 2, 2020) (See Section 6).
  - For consumer watches, ECG diagnosis is considered a prediagnostic pending medical verification and not designed to be acted on without clinician review.
  - ECG classification of other arrhythmias (premature ventricular complexes (PVCs), premature atrial complexes (PACs), ventricular tachycardia (VT)) is currently unavailable.

#### 2.2.2 Non‐ECG‐based

##### 2.2.2.1 Photoplethysmography

Consumer devices such as smartphones and smartwatches require accessories and often extra cost for conversion into rhythm monitoring tools. In contrast, the PPG technologies allow for the detection of arrhythmias using hardware already present on most consumer devices (smartwatches and fitness bands) through a downloadable application. PPG is an optical technique that can be used to detect AF by measuring and analyzing a peripheral pulse waveform. Using a light source and a photodetector, the pulse waveform can be measured by detecting changes in the light intensity, which reflects the tissue blood volume of a skin surface such as the fingertip, earlobe, or face.^39^, ^40^ An automated algorithm can subsequently analyze the generated pulse waveform to detect AF. PPG avoids the instability and motion artifacts of ECG sensors and can be passively and opportunistically measured.

This technology has been applied for use with smartphones using the phone’s camera to measure a fingertip pulse waveform. Rapid irregularly conducted AF may produce variable pulse pressures that challenge detection.^61^ The performance of algorithms interpreting these PPG signals has been proven to be in high agreement with ECG rhythm strips.^40^, ^42^, ^43^ The smartphone‐based PPG applications have been utilized in at‐risk population to detect AF and as a screening tool in the general population^64^ (See Section 6).

The PPG technology has also been incorporated in smartwatches to measure heart rate and rhythm.^45^, ^46^ Some have developed prototypes of a band that includes a single‐channel ECG, multi‐wavelength PPG, and tri‐axial accelerometry recording simultaneously at 128 Hz^67^ and others use a deep‐neural network based on PPG sensors to detect AF (https://www.mobihealthnews.com/content/study‐apple‐watch‐paired‐deep‐neural‐network‐detects‐atrial‐fibrillation‐97‐percent‐accuracy; https://mrhythmstudy.org). If PPG or optical sensors and detection algorithms can match the performance of ECG‐based rhythm assessment, delivery of AF care may be expected to change substantially and drive a radical departure from relying on an office or ambulatory ECG for ascertainment of AF.

##### 2.2.2.2 Oscillometry

Blood pressure (BP) measurements can be erratic when the pulse is irregular. This characteristic is utilized by automatic oscillometric BP monitors that derive heart rhythm regularity algorithmically.^68^ Automated BP monitors have been used for opportunistic AF detection. Studies have shown that six devices from two manufacturers were reliable with sensitivities and specificities greater than 85%.^69^ These studies suggested that BP devices with embedded algorithms for detecting arrhythmias show promise as screening tools for AF, comparing favorably with manual pulse palpation. Such capability could be added to continuous BP recording devices.^70^ One device identifies possible AF when at least two of three consecutive measurements show pulse irregularity. Several studies addressed the diagnostic accuracy^31^, ^48^, ^51^, ^52^, ^53^, ^54^, ^55^, ^56^, ^57^ and the feasibility of this device as a screening tool.^31^, ^55^, ^58^

The following have undergone preliminary study:

##### 2.2.2.3 Mechanocardiography

Mechanocardiography uses accelerometers and gyroscopes to sense the mechanical activity of the heart. The accuracy of this technology to detect AF using a smartphone’s built‐in accelerometer and gyroscope sensors was assessed in a proof of concept study.^79^ A smartwatch (Sony Experia) was placed on the chest in supine patients to detect micro movements of the chest. Possibly, carrying this device in a pocket may have utility but is likely to be confounded by movement (e.g., walking) artifacts.

##### 2.2.2.4 Contactless video plethysmography

Noncontact video monitoring of respiration and heart rate have been developed less than 15 years ago.^60^, ^61^ In 2014, a pioneering article described the concept of contactless videobased detection of AF.^82^ Deep learning of a video of a person’s face can identify AF by examining irregularity of pulsatile facial perfusion.^83^ It is a monitoring technique extracting the photoplethysmographic‐like signals from a standard digital RGB video recording of the human skin and specifically of an individual’s face. The videoplethysmographic signal describes the absorption peak of ambient light by the hemoglobin from the facial skin. Several studies have been performed to develop a method that is sensitive enough to detect each cardiac pulse and provide insights into variability on pulse on a beat‐to‐beat basis. The HealthKam works using HUE color space from video cameras^64^, ^65^ and can easily be integrated to any portable computer device with a camera (smartphone, tablet, etc.). By using mobile devices with cameras, the deployment of the technology is easy and scalable since it does not require the use and distribution of any physical devices. Such a system may change the approach to AF screening, which currently is only 1 patient at a time. High‐throughput AF detection from multiple patients concurrently using a single digital camera and a pretrained deep convolutional neural network (DCNN) was feasible in a pilot study.^86^

###### Limitations

One requirement for these technologies is steady focus: Thus moving subjects present a challenge. It is important to avoid recording, sending, or communicating any video of the patient thus protecting privacy and dignity. Video‐based technologies in telemedicine have raised a new set of societal and ethical concerns that are being continuously re‐evaluated such as during the COVID‐19 pandemic. Issues regarding privacy, confidentiality, and legal and ethical obligation to treat are crucial factors to be considered when these technologies are deployed at larger scale.^87^

##### 2.2.2.5 Smart speakers

There are preliminary reports on using commodity smart devices to identify agonal breathing.^68^, ^69^ Identification of abnormal heart rate patterns may be made possible by converting smart speakers into a sonar device with emission of in‐audible frequencies sound waves and receiving them to detect motion. These are not in consumer domain but potentially have wide scalability.

REFERENCES SECTION 21

Steinberg
JS
, 
Varma
N
, 
Cygankiewicz
I
, 
Aziz
P
, 
Balsam
P
, 
Baranchuk
A
, …
Piotrowicz
R
. ISHNE‐HRS expert consensus statement on ambulatory ECG and external cardiac monitoring/telemetry. Annals of Noninvasive Electrocardiology. 2017;22(3):e12447. 10.1111/anec.12447.PMC6931745284806322

Musat
DL
, 
Milstein
N
, 
Mittal
S
. Implantable loop recorders for cryptogenic stroke (plus real‐world atrial fibrillation detection rate with implantable loop recorders). Cardiac Electrophysiology Clinics. 2018;10:111–118. 10.1016/j.ccep.2017.11.011.294281323

Sakhi
R
, 
Theuns
DAMJ
, 
Szili‐Torok
T
, 
Yap
SC
. Insertable cardiac monitors: Current indications and devices. Expert Review of Medical Devices. 2019;16:45–55. 10.1080/17434440.2018.1557046.305223504

Tomson
TT
, 
Passman
R
. The reveal LINQ insertable cardiac monitor. Expert Review of Medical Devices. 2015;12:7–18. 10.1586/17434440.2014.953059.251549705

Ciconte
G
, 
Saviano
M
, 
Giannelli
L
, 
Calovic
Z
, 
Baldi
M
, 
Ciaccio
C
, …
Pappone
C
. Atrial fibrillation detection using a novel three‐vector cardiac implantable monitor: The atrial fibrillation detect study. Europace. 2017;19:1101–1108. 10.1093/europace/euw181.277028656

Hindricks
G
, 
Pokushalov
E
, 
Urban
L
, 
Taborsky
M
, 
Kuck
KH
, 
Lebedev
D
, …
Pürerfellner
H
. XPECT Trial Investigators. Performance of a new leadless implantable cardiac monitor in detecting and quantifying atrial fibrillation: Results of the XPECT trial. Circulation Arrhythmia and Electrophysiology. 2010;3:141–147. 10.1161/circep.109.877852.201601697

Mittal
S
, 
Rogers
J
, 
Sarkar
S
, 
Koehler
J
, 
Warman
EN
, 
Tomson
TT
, 
Passman
RS
. Real‐world performance of an enhanced atrial fibrillation detection algorithm in an insertable cardiac monitor. Heart Rhythm. 2016;13:1624–1630. 10.1016/j.hrthm.2016.05.010.271656948

Nölker
G
, 
Mayer
J
, 
Boldt
L‐H
, 
Seidl
K
, 
Van driel
V
, 
Massa
T
, …
Lewalter
T
. Performance of an implantable cardiac monitor to detect atrial fibrillation: Results of the DETECT AF study. Journal of Cardiovascular Electrophysiology. 2016;27:1403–1410. 10.1111/jce.13089.275651199

Sanders
P
, 
Purerfellner
H
, 
Pokushalov
E
, 
Sarkar
S
, 
Di Bacco
M
, 
Maus
B
, 
Dekker
LR
. Reveal LINQ Usability Investigators. Performance of a new atrial fibrillation detection algorithm in a miniaturized insertable cardiac monitor: Results from the Reveal LINQ Usability Study. Heart Rhythm. 2016;13:1425–30. 10.1016/j.hrthm.2016.03.005.2696129810

Doliwa
PS
, 
Frykman
V
, 
Rosenqvist
M
. Short‐term ECG for out of hospital detection of silent atrial fibrillation episodes. Scandinavian Cardiovascular Journal. 2009;43:163–168.1909697710.1080/1401743080259343511

Tieleman
RG
, 
Plantinga
Y
, 
Rinkes
D
, 
Bartels
GL
, 
Posma
JL
, 
Cator
R
, …
Houben
RP
. Validation and clinical use of a novel diagnostic device for screening of atrial fibrillation. Europace. 2014;16:1291–1295.2482576610.1093/europace/euu057PMC414960812

Desteghe
L
, 
Raymaekers
Z
, 
Lutin
M
, 
Vijgen
J
, 
Dilling‐Boer
D
, 
Koopman
P
, …
Heidbuchel
H
. Performance of handheld electrocardiogram devices to detect atrial fibrillation in a cardiology and geriatric ward setting. Europace. 2017;19:29–39. 10.1093/europace/euw025.2689349613

Hendrikx
T
, 
Rosenqvist
M
, 
Wester
P
, 
Sandstrom
H
, 
Hornsten
R
. Intermittent short ECG recording is more effective than 24‐hour Holter ECG in detection of arrhythmias. BMC Cardiovascular Disorders. 2014;14:41. 10.1186/1471-2261-14-41.24690488PMC423432514

Kaasenbrood
F
, 
Hollander
M
, 
Rutten
FH
, 
Gerhards
LJ
, 
Hoes
AW
, 
Tieleman
RG
. Yield of screening for atrial fibrillation in primary care with a hand‐held, single‐lead electrocardiogram device during influenza vaccination. Europace. 2016;18:1514–1520. 10.1093/europace/euv426.26851813PMC507213515

Poulsen
MB
, 
Binici
Z
, 
Dominguez
H
, 
Soja
AM
, 
Kruuse
C
, 
Hornnes
AH
, …
Overgaard
K
. Performance of short ECG recordings twice daily to detect paroxysmal atrial fibrillation in stroke and transient ischemic attack patients. International Journal of Stroke. 2017;12:192–196. 10.1177/1747493016669883.2769431216

Svennberg
E
, 
Stridh
M
, 
Engdahl
J
, 
Al‐Khalili
F
, 
Friberg
L
, 
Frykman
V
, 
Rosenqvist
M
. Safe automatic one‐lead electrocardiogram analysis in screening for atrial fibrillation. Europace. 2017;19:1449–1453. 10.1093/europace/euw286.2833957817

Tavernier
R
, 
Wolf
M
, 
Kataria
V
, 
Phlips
T
, 
Huys
R
, 
Taghji
P
, …
Duytschaever
M
. Screening for atrial fibrillation in hospitalised geriatric patients. Heart. 2018;104:588–593. 10.1136/heartjnl-2017-311981.2888303218

Vaes
B
, 
Stalpaert
S
, 
Tavernier
K
, 
Thaels
B
, 
Lapeire
D
, 
Mullens
W
, 
Degryse
J
. The diagnostic accuracy of the MyDiagnostick to detect atrial fibrillation in primary care. BMC Family Practice. 2014;15:113. 10.1186/1471-2296-15-113.24913608PMC406934019

Barrett
PM
, 
Komatireddy
R
, 
Haaser
S
, 
Topol
S
, 
Sheard
J
, 
Encina
SJ
, 
Topol
EJ
. Comparison of 24‐hour Holter monitoring with 14‐day novel adhesive patch electrocardiographic monitoring. The American Journal of Medicine. 2014;127:95.e11–e17. 10.1016/j.amjmed.2013.10.003.PMC38821982438410820

Turakhia
MP
, 
Hoang
DD
, 
Zimetbaum
P
, 
Miller
JD
, 
Froelicher
VF
, 
Kumar
UN
, …
Heidenreich
PA
. Diagnostic utility of a novel leadless arrhythmia monitoring device. American Journal of Cardiology. 2013;112:520–524. 10.1016/j.amjcard.2013.04.017.2367298821

Rosenberg
MA
, 
Samuel
M
, 
Thosani
A
, 
Zimetbaum
PJ
. Use of a noninvasive continuous monitoring device in the management of atrial fibrillation: A pilot study. Pacing and Clinical Electrophysiology. 2013;36:328–333.2324082710.1111/pace.12053PMC361837222

Turakhia
MP
, 
Ullal
AJ
, 
Hoang
DD
, 
Than
CT
, 
Miller
JD
, 
Friday
KJ
, …
Heidenreich
PA
. Feasibility of extended ambulatory electrocardiogram monitoring to identify silent atrial fibrillation in high‐risk patients: The Screening Study for Undiagnosed Atrial Fibrillation (STUDY‐AF). Clinical Cardiology. 2015;38:285–292.2587347610.1002/clc.22387PMC465433023

Rooney
MR
, 
Soliman
EZ
, 
Lutsey
PL
, 
Norby
FL
, 
Loehr
LR
, 
Mosley
TH
, …
Chen
LY
. Prevalence and characteristics of subclinical atrial fibrillation in a community‐dwelling elderly population: The ARIC study. Circulation: Arrhythmia and Electrophysiology. 2019;12:e007390. 10.1161/CIRCEP.119.007390.31607148PMC681438724

Stehlik
J
, 
Schmalfuss
C
, 
Bozkurt
B
, 
Nativi‐Nicolau
J
, 
Wohlfahrt
P
, 
Wegerich
S
, …
Pham
M
. Continuous wearable monitoring analytics predict heart failure hospitalization: The LINK‐HF multicenter study. Circulation Heart Failure. 2020;13:e006513. 10.1161/CIRCHEARTFAILURE.119.006513.3209350625

Elliot
CA
, 
Hamlin
MJ
, 
Lizamore
CA
. Validity and reliability of the hexoskin wearable biometric vest during maximal aerobic power testing in elite cyclists. The Journal of Strength & Conditioning Research. 2019;33:1437–1444. 10.1519/JSC.0000000000002005.2875953826

Eysenck
W
, 
Freemantle
N
, 
Sulke
N
. A randomized trial evaluating the accuracy of AF detection by four external ambulatory ECG monitors compared to permanent pacemaker AF detection. Journal of Interventional Cardiac Electrophysiology. 2019;57:361–369. 10.1007/s10840-019-00515-0.3074136027

Fabregat‐Andres
O
, 
Munoz‐Macho
A
, 
Adell‐Beltran
G
, 
Ibanez‐Catala
X
, 
Macia
A
, 
Facila
L
. Evaluation of a new shirt‐based electrocardiogram device for cardiac screening in soccer players: Comparative study with treadmill ergospirometry. Cardiology Research. 2014;5:101–107. 10.14740/cr333w
28348705PMC535817028

Feito
Y
, 
Moriarty
TA
, 
Mangine
G
, 
Monahan
J
. The use of a smart‐textile garment during high‐intensity functional training: A pilot study. The Journal of Sports Medicine and Physical Fitness. 2019;59:947–954. 10.23736/S0022-4707.18.08689-9
3002412529

Pagola
J
, 
Juega
J
, 
Francisco‐Pascual
J
, 
Moya
A
, 
Sanchis
M
, 
Bustamante
A
, …
Molina
CA
. CryptoAF investigators. Yield of atrial fibrillation detection with Textile Wearable Holter from the acute phase of stroke: Pilot study of Crypto‐AF registry. International Journal of Cardiology. 2018;251:45–50. 10.1016/j.ijcard.2017.10.063.2910736030

Varma
N
, 
Marrouche
NF
, 
Aguinaga
L
, 
Albert
CM
, 
Arbelo
E
, 
Choi
JI
, 
Varosy
PD
. HRS/EHRA/APHRS/LAHRS/ACC/AHA worldwide practical guidance for telehealth and arrhythmia monitoring during and after a pandemic. Journal of the American College of Cardiology. 2020;76:1363–1374. 10.1016/j.jacc.2020.06.019.32534936PMC728908831

Chan
PH
, 
Wong
CK
, 
Pun
L
, 
Wong
YF
, 
Wong
MM
, …
Siu
CW
. Head‐to‐head comparison of the AliveCor heart monitor and microlife WatchBP office AFIB for atrial fibrillation screening in a primary care setting. Circulation. 2017;135:110–112. 10.1161/CIRCULATIONAHA.116.024439.2802806632

Lowres
N
, 
Neubeck
L
, 
Salkeld
G
, 
Krass
I
, 
McLachlan
AJ
, 
Redfern
J
, 
Freedman
SB
. Feasibility and cost‐effectiveness of stroke prevention through community screening for atrial fibrillation using iPhone ECG in pharmacies. The SEARCH‐AF study. Thrombosis and Haemostasis. 2014;111:1167–1176. 10.1160/th14-03-0231.2468708133

Tarakji
KG
, 
Wazni
OM
, 
Callahan
T
, 
Kanj
M
, 
Hakim
AH
, 
Wolski
K
, …
Lindsay
BD
. Using a novel wireless system for monitoring patients after the atrial fibrillation ablation procedure: The iTransmit study. Heart Rhythm. 2015;12:554–559. 10.1016/j.hrthm.2014.11.015.2546085434

Chan
PH
, 
Wong
CK
, 
Pun
L
, 
Wong
YF
, 
Wong
MM
, …
Siu
CW
. Head‐to‐head comparison of the AliveCor heart monitor and microlife WatchBP office AFIB for atrial fibrillation screening in a primary care setting. Circulation. 2017;135:110–112. 10.1161/CIRCULATIONAHA.116.024439.2802806635

Halcox
JPJ
, 
Wareham
K
, 
Cardew
A
, 
Gilmore
M
, 
Barry
JP
, 
Phillips
C
, 
Gravenor
MB
. Assessment of remote heart rhythm sampling using the alivecor heart monitor to screen for atrial fibrillation: The REHEARSE‐AF Study. Circulation. 2017;136:1784–1794. 10.1161/circulationaha.117.030583.2885172936

Chung
EH
, 
Guise
KD
. QTC intervals can be assessed with the AliveCor heart monitor in patients on dofetilide for atrial fibrillation. Journal of Electrocardiology. 2015;48:8–9. 10.1016/j.jelectrocard.2014.10.005.2545319437

Garabelli
P
, 
Stavrakis
S
, 
Albert
M
, 
Koomson
E
, 
Parwani
P
, 
Chohan
J
, …
Po
S
. Comparison of QT interval readings in normal sinus rhythm between a smartphone heart Monitor and a 12‐Lead ECG for healthy volunteers and inpatients receiving sotalol or dofetilide. Journal of Cardiovascular Electrophysiology. 2016;27:827–832. 10.1111/jce.12976.2702765338

Koltowski
L
, 
Balsam
P
, 
Glłowczynska
R
, 
Rokicki
JK
, 
Peller
M
, 
Maksym
J
, …
Grabowski
M
. Kardia Mobile applicability in clinical practice: A comparison of Kardia Mobile and standard 12‐lead electrocardiogram records in 100 consecutive patients of a tertiary cardiovascular care center. Cardiology Journal. 2013; 10.5603/CJ.a2019.0001.PMC82769943064407939

Conroy
T
, 
Guzman
JH
, 
Hall
B
, 
Tsouri
G
, 
Couderc
JP
. Detection of atrial fibrillation using an earlobe photoplethysmographic sensor. Physiological Measurement. 2017;38:1906–1918. 10.1088/1361-6579/aa8830.2883650740

McManus
DD
, 
Lee
J
, 
Maitas
O
, 
Esa
N
, 
Pidikiti
R
, 
Carlucci
A
, …
Chon
KH
. A novel application for the detection of an irregular pulse using an iPhone 4S in patients with atrial fibrillation. Heart Rhythm. 2013;10:315–319. 10.1016/j.hrthm.2012.12.001.23220686PMC369857041

Choi
A
, 
Shin
H
. Photoplethysmography sampling frequency: Pilot assessment of how low can we go to analyze pulse rate variability with reliability?
Physiol Measurement. 2017;38:586–600.10.1088/1361-6579/aa5efa2816983642

McManus
DD
, 
Chong
JW
, 
Soni
A
, 
Saczynski
JS
, 
Esa
N
, 
Napolitano
C
, …
Chon
KH
. PULSE‐SMART: Pulse‐based arrhythmia discrimination using a novel smartphone application. Journal of Cardiovascular Electrophysiology. 2016;27:51–57.2639172810.1111/jce.12842PMC476831043

Proesmans
T
, 
Mortelmans
C
, 
Van Haelst
R
, 
Verbrugge
F
, 
Vandervoort
P
, 
Vaes
B
. Mobile phone‐based use of the photoplethysmography technique to detect atrial fibrillation in primary care: Diagnostic accuracy study of the fibricheck app. Journal of Medical Internet Research mHealth and uHealth. 2019;7:e12284. 10.2196/12284.PMC64568253091665644

Verbrugge
FH
, 
Proesmans
T
, 
Vijgen
J
, 
Mullens
W
, 
Rivero‐Ayerza
M
, 
Van Herendael
H
, …
Nuyens
D
. Atrial fibrillation screening with photo‐plethysmography through a smartphone camera. Europace. 2019;21:1167–1175. 10.1093/europace/euz119.3105667845

Dörr
M
, 
Nohturfft
V
, 
Brasier
N
, 
Bosshard
E
, 
Djurdjevic
A
, 
Gross
S
, …
Eckstein
J
. The WATCH AF trial: SmartWATCHes for detection of atrial fibrillation. Journal of the American College of Cardiology Clinical Electrophysiology. 2019;5:199–208. 10.1016/j.jacep.2018.10.006.3078469146

Guo
Y
, 
Wang
H
, 
Zhang
H
, 
Liu
T
, 
Liang
Z
, 
Xia
Y
, …
Lip
GYH
. MAFA II Investigators. Mobile photoplethysmographic technology to detect atrial fibrillation. Journal of the American College of Cardiology. 2019;74:2365–2375. 10.1016/j.jacc.2019.08.019.3148754547

Nemati
S
, 
Ghassemi
MM
, 
Ambai
V
, 
Isakadze
N
, 
Levantsevych
O
, 
Shah
A
, 
Clifford
GD
. Monitoring and detecting atrial fibrillation using wearable technology. Conference proceedings: Annual International Conference of the IEEE Engineering in Medicine and Biology Society IEEE Engineering in Medicine and Biology Society Annual Conference. 2016;2016:3394–3397. 10.1109/embc.2016.7591456.2826903248

Chen
Y
, 
Lei
L
, 
Wang
JG
. Atrial fibrillation screening during automated blood pressure measurement‐Comment on “Diagnostic accuracy of new algorithm to detect atrial fibrillation in a home blood pressure monitor”. The Journal of Clinical Hypertension. 2017;19:1148–1151. 10.1111/jch.13081.28942614PMC803128349

Kane
SA
, 
Blake
JR
, 
McArdle
FJ
, 
Langley
F
, 
Sims
AJ
. Opportunistic detection of atrial fibrillation using blood pressure monitors: A systematic review. Open Heart. 2016;3:e000362.2709976010.1136/openhrt-2015-000362PMC483630550

Kario
K
. Evidence and perspectives on the 24‐hour management of hypertension: Hemodynamic biomarker‐initiated ‘anticipation medicine’ for zero cardiovascular event. Progress in Cardiovascular Diseases. 2016;59:262–281. 10.1016/j.pcad.2016.04.001.2708020251

Gandolfo
C
, 
Balestrino
M
, 
Bruno
C
, 
Finocchi
C
, 
Reale
N
. Validation of a simple method for atrial fibrillation screening in patients with stroke. Neurological Sciences. 2015;36:1675–1678. 10.1007/s10072-015-2231-0.2592607252

Kearley
K
, 
Selwood
M
, 
Van den Bruel
A
, 
Thompson
M
, 
Mant
D
, 
Hobbs
FR
, …
Heneghan
C
. Triage tests for identifying atrial fibrillation in primary care: A diagnostic accuracy study comparing single‐lead ECG and modified BP monitors. British Medical Journal Open. 2014;4:e004565. 10.1136/bmjopen-2013-004565.PMC40254112479325053

Marazzi
G
, 
Iellamo
F
, 
Volterrani
M
, 
Lombardo
M
, 
Pelliccia
F
, 
Righi
D
, …
Rosano
G
. Comparison of Microlife BP A200 Plus and Omron M6 blood pressure monitors to detect atrial fibrillation in hypertensive patients. Advances in Therapy. 2012;29:64–70. 10.1007/s12325-011-0087-0.2219890254

Stergiou
GS
, 
Karpettas
N
, 
Protogerou
A
, 
Nasothimiou
EG
, …
Kyriakidis
M
. Diagnostic accuracy of a home blood pressure monitor to detect atrial fibrillation. Journal of Human Hypertension. 2009;23:654–8. 10.1038/jhh.2009.5.1927966155

Omboni
S
, 
Verberk
WJ
. Opportunistic screening of atrial fibrillation by automatic blood pressure measurement in the community. British Medical Journal Open. 2016;6:e010745. 10.1136/bmjopen-2015-010745.PMC48387272707257156

Wiesel
J
, 
Fitzig
L
, 
Herschman
Y
, 
Messineo
FC
. Detection of atrial fibrillation using a modified microlife blood pressure monitor. American Journal of Hypertension. 2009;22:848–852. 10.1038/ajh.2009.98.1947879357

Wiesel
J
, 
Arbesfeld
B
, 
Schechter
D
. Comparison of the Microlife blood pressure monitor with the Omron blood pressure monitor for detecting atrial fibrillation. American Journal of Cardiology. 2014;114:1046–8. 10.1016/j.amjcard.2014.07.016.2521254658

Wiesel
J
, 
Salomone
TJ
. Screening for atrial fibrillation in patients ≥65 years using an automatic blood pressure monitor in a skilled nursing facility. American Journal of Cardiology. 2017;120:1322–1324. 10.1016/j.amjcard.2017.07.016.2882135159

Jaakkola
J
, 
Jaakkola
S
, 
Lahdenoja
O
, 
Hurnanen
T
, 
Koivisto
T
, 
Pankaala
M
, …
Airaksinen
KEJ
. Mobile phone detection of atrial fibrillation with mechanocardiography: The MODE‐AF Study (mobile phone detection of atrial fibrillation). Circulation. 2018;137:1524–1527. 10.1161/circulationaha.117.032804.2952683460

Takano
C
, 
Ohta
Y
. Heart rate measurement based on a time‐lapse image. Medical Engineering & Physics ‐ Journal. 2007;29:853–857.10.1016/j.medengphy.2006.09.0061707452561

Verkruysse
W
, 
Svaasand
LO
, 
Nelson
JS
. Remote plethysmographic imaging using ambient light. Optics Express. 2008;16:21434–21445.1910457310.1364/oe.16.021434PMC271785262

Couderc
J‐P
, 
Kyal
S
, 
Mestha
LK
, 
Xu
B
, 
Peterson
DR
, 
Xia
X
, 
Hall
B
. Detection of atrial fibrillation using contactless facial video monitoring. Heart Rhythm. 2015;12:195–201.2517948810.1016/j.hrthm.2014.08.03563

Yan
BP
, 
Lai
WHS
, 
Chan
CKY
, 
Chan
SC
, 
Chan
LH
, 
Lam
KM
, …
Poh
MZ
. Contact‐free screening of atrial fibrillation by a smartphone using facial pulsatile photoplethysmographic signals. Journal of the American Heart Association. 2018;5(7):e008585. 10.1161/JAHA.118.008585.PMC60154142962259264

Dautov
R
, 
Savur
C
, &
Tsouri
G
. On the Effect of Face Detection on Heart Rate Estimation inVideoplethysmography. 2018. *IEEE Western New York Image and Signal ProcessingWorkshop (WNYISPW)*. 10.1109/wnyipw.2018.8576439
65

Tsouri
GR
, 
Li
Z
. On the benefits of alternative color spaces for noncontact heart rate measurements using standard red‐green‐blue cameras. Journal of Biomedical Optics. 2015;20:48002.10.1117/1.JBO.20.4.0480022587562866

Yan
BP
, 
Lai
WHS
, 
Chan
CKY
, 
Au
ACK
, 
Freedman
B
, 
Poh
YC
, 
Poh
MZ
. High‐throughput, contact‐free detection of atrial fibrillation from video with deep learning. Journal of the American Medical Association Cardiology. 2020;5:105–107. 10.1001/jamacardio.2019.4004.31774461PMC690212367

Turakhia
MP
, 
Desai
M
, 
Hedlin
H
, 
Rajmane
A
, 
Talati
N
, 
Ferris
T
, …
Perez
MV
. Rationale and design of a large‐scale, app‐based study to identify cardiac arrhythmias using a smartwatch: The Apple Heart Study. American Heart Journal. 2019;207:66–75.3039258410.1016/j.ahj.2018.09.002PMC809904868

Chan
J
, 
Rea
T
, 
Gollakota
S
, 
Sunshine
JE
. Contactless cardiac arrest detection using smart devices. NPJ Digital Medicine. 2019;2:52. 10.1038/s41746-019-0128-7.31304398PMC658458269

Wang
A
, 
Sunshine
JE
, 
Gollakota
S
.Contactless infant monitoring using white noise. Availabe at: https://homes.cs.washington.edu/~gshyam/Papers/whitenoise.pdf.70

Steinberg
JS
, 
Varma
N
, 
Cygankiewicz
I
, 
Aziz
P
, 
Balsam
P
, 
Baranchuk
A
, …
Piotrowicz
R
. 2017 ISHNE‐HRS expert consensus statement on ambulatory ECG and external cardiac monitoring/telemetry. Annals of Noninvasive Electrocardiology. 2017;22:e12447. 10.1111/anec.12447.PMC693174528480632

## 3 mHEALTH APPLICATIONS FOR ARRHYTHMIAS

Typically, most patients with palpitations and dizziness are evaluated using the various technologies reviewed in Section 2.1.^90^ Devices capable of recording at least one ECG lead allow the interpreting clinician to distinguish between wide‐ and narrow‐complex rhythms, bradycardia, and tachycardia, and thus distinguish between the various causative rhythms. Smart devices may be useful in pediatric patients.^91^

### 3.1 Atrial fibrillation

**FIGURE 5:**
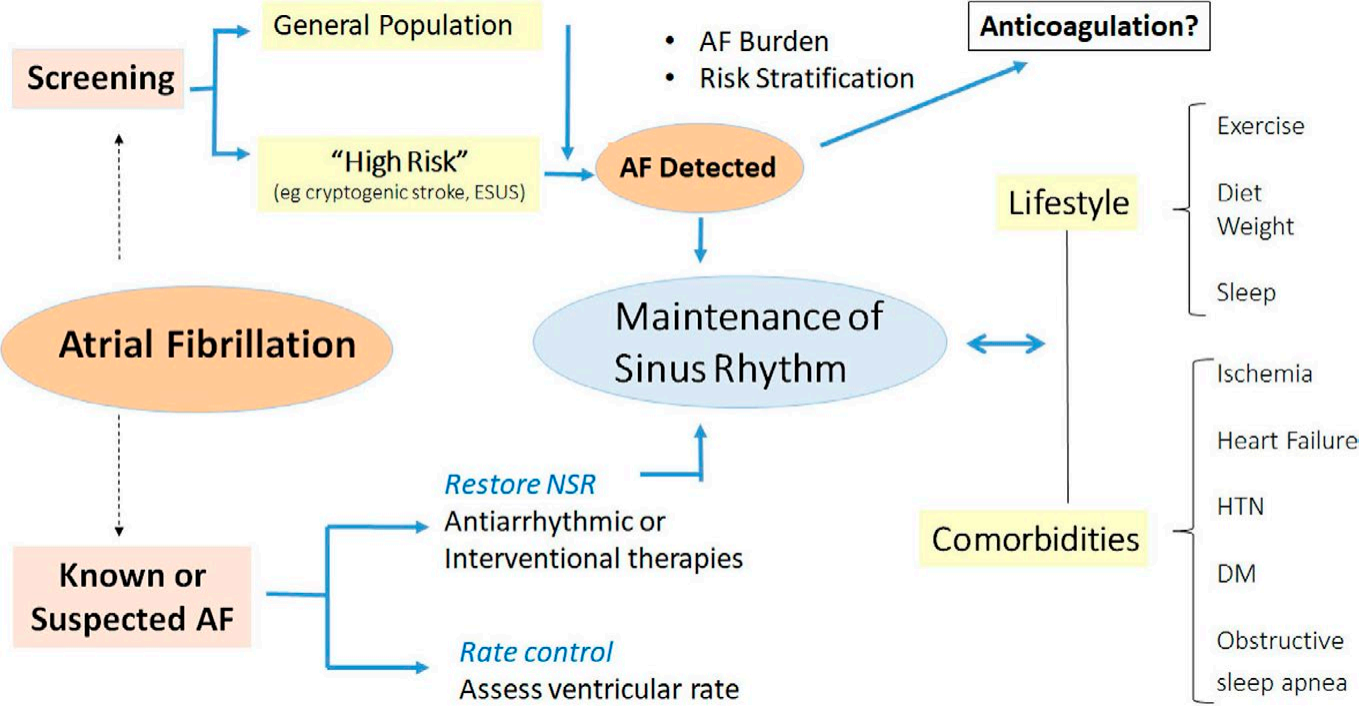
mHealth and AF. Applications include screening for AF in general or high‐risk populations, managing comorbidities and lifestyles important for prevention and control, as well as managing treatment of known AF. AF—atrial fibrillation, DM—diabetes, HTN— hypertension, NSR—normal sinus rhythm.

The disease is often intermittent and asymptomatic, which may delay diagnosis.^92‐94^ lead to incorrect estimation of AF burden,^95,96^ and pose management challenges to healthcare services, thereby exposing the patient to the consequences of untreated AF. New digital health and sensor technologies have the potential for early identification of AF, opening up opportunities for screening, which then can be tied to evidence‐based management. These may be directed to several broad groups: for screening the general population or managing the already diagnosed, for following responses to treatment, and increasingly to managing comorbidities and lifestyle modification (See Section 4) (Figure 5). mHealth mechanisms may facilitate understanding the relation between AF burden, its progression, and cardiovascular risk.^97^

#### 3.1.1 Undiagnosed atrial fibrillation identification

Classical epidemiological data point to the notion that early identification of AF has the potential to improve morbidity and possibly mortality. (1) AF is associated with a 5‐fold increased risk of stroke^98^ and doubled mortality^99^; (2) The prevalence of undiagnosed AF is at least 1.5% for patients >65 years;^100^ (3) In about a quarter of all AF‐related strokes, the stroke is the first manifestation of the arrhythmia;^101^ while other AF patients present first with congestive HF; (4) Stroke risk is independent of symptoms;^102^ (5) Diagnosis often requires repeated or prolonged ECG monitoring; and (6) Oral anticoagulants (OACs) are highly effective in reducing the risk of cardioembolic stroke, mortality, and possibly dementia in the setting of AF.^13^, ^14^

Atrial fibrillation identification depends on factors having to do with the arrhythmia itself, that is the combination of AF prevalence and density,^105^ and factors associated with detection such as the frequency and duration of monitoring and diagnostic test performance.^106^ Several studies including patients with variable stroke risk factors have used mHealth technologies to identify undiagnosed AF (Tables 2 and 3), but these may require gold‐standard ECG confirmation.

**TABLE 3:** Selected screening studies for atrial fibrillation using newer technologies

	Device	Author, year	Setting	Inclusion criteria	N	Mean age (years)	Duration of monitoring	New AF detection (%)
Handheld ECG device	Zenicor SL	Berge et al.^107^	Norway systematic	Age 63‐65 years,_ CHADS‐VaSC≥2 (M) or ≥3 (F)	1510	64	10 sec Twice daily for 2 weeks	0.9%
	Zenicor SL	Svennberg et al.^108^	Sweden systematic	Age 75‐76 years	7173	75	10 sec Twice daily for 2 weeks	3.0%
	Zenicor SL	Engdahl et al.^109^	Sweden systematic	Age 75‐76 years, CHADS2 risk score≥2	403	75	10 sec Twice daily for 2 weeks	7.4%
	Zenicor SL	Gudmundsdottir et al.^110^	Sweden systematic	Age 75‐76 years + NTproBNP≥125 ng/l	3766	75	10 sec Twice daily for 2 weeks	4.4%
	Zenicor SL	Doliwa et al.^111^	Sweden Postdischarge	Recent ischemic stroke/TIA and no prior AF	249	72	10 sec 30 days	4.8%
	My Diagnostick	Tieleman et al.^112^	Netherlands	Influenza vaccination	676	74	1 min	1.6%
	My Diagnostick	Kaasenbrood et al.^113^	Netherlands Primary care Opportunistic	Age>65 years	919		1 min	1.43%
	My Diagnostick SL	Tavernier et al.^114^	Belgium Geriatric ward	Geriatric	252	84	Daily 1 min during hospitalization (median 5 )	13%
ECG Patch	ZioPatch iRhythm	Turakhia et al.^115^ (STUDY‐AF)	US	M, age ≥65 years and ≥risk factors	75	69	Two weeks continuous	5.3%
	ZioPatch iRhythm	Steinhubl et al.^116^	US National health plan members	Age ≥75 years or M>55/F>65 years+risk factors	2659	72	Continuous 4 weeks	2.4%
	Zio XT Patch	Rooney et al.^117^ ARIC study	US Community surveillance study	No prior AF	386	79	Continuous 2‐4 weeks	2.5% (2 weeks) 4% (4 weeks)
	Zio Patch	Heckbert et al.^118^ Multi‐Ethnic Study of Atherosclerosis	US Community surveillance study	No prior AF	804	75	Continuous 2‐4 weeks	4% (AF/AFL)
Smartphone ECG‐based	AliveCor Kardia Mobile SL	Lowres et al.^119^ (SEARCH‐AF)	Australia Pharmacy Opportunistic	Age≥65 years	1000	76	30 sec	1.5%
	AliveCor Kardia Mobile SL	Chan et al.^120^	Hong Kong Outpatient clinic	Age ≥65 years or HTN/diabetes	1013	68	30 sec	0.5%
	AliveCor KardiaMobile SL	Halcox et al.^123^ (REHEARSE AF)	UK Randomized trial	Age≥65 years +CHA 2DS2‐VASc≥2	1001	73	30 sec Twice weekly for 1 year	3.8%
Smartphone device PPG‐based	Cardio Mobile app	Chan et al.^120^	Hong Kong Outpatient	Age years ≥65 years or HTN	1013	68	30 sec	0.5%
	Huawei wristband (Honor Band 4) or Huawei Watch	Guo et al.^66^	General population across China	Age >18 years	187,912	35	≥14 days	0.23%
Smartwatch	Apple smartwatch, iPhone app	Perez et al.^122^	General population across USA	Age >22 years	419,297	41	Median 117 days	0.52% irregular heart rhythm

Abbreviations: AF, atrial fibrillation; AFL, atrial flutter; F, females; HTN, hypertension; M, males; TIA, transient ischaemic attack.

REFERENCES TABLE 31

Gropler
MRF
, 
Dalal
AS
, 
Van Hare
GF
, 
Silva
JNA
. Can smartphone wireless ECGs be used to accurately assess ECG intervals in pediatrics? A comparison of mobile health monitoring to standard 12‐lead ECG. PLoS One. 2018;13:e0204403. 10.1371/journal.pone.0204403.30260996PMC61600472

McCabe
PJ
, 
Chamberlain
AM
, 
Rhudy
L
, 
DeVon
HA
. Symptom representation and treatment‐seeking prior to diagnosis of atrial fibrillation. Western Journal of Nursing Research. 2015;38:200–215.2569417710.1177/01939459155703683

Strickberger
SA
, 
Ip
J
, 
Saksena
S
, 
Curry
K
, 
Bahnson
TD
, 
Ziegler
PD
. Relationship between atrial tachyarrhythmias and symptoms. Heart Rhythm. 2005;2:125–131.1585128310.1016/j.hrthm.2004.10.0424

Verma
A
, 
Champagne
J
, 
Sapp
J
, 
Essebag
V
, 
Novak
P
, 
Skanes
A
, …
Birnie
D
. Discerning the incidence of symptomatic and asymptomatic episodes of atrial fibrillation before and after catheter ablation (DISCERN AF): A prospective, multicenter study. Journal of the American Medical Association Internal Medicine. 2013;173:149–156. 10.1001/jamainternmed.2013.1561.232665975

Boriani
G
, 
Laroche
C
, 
Diemberger
I
, 
Fantecchi
E
, 
Popescu
MI
, 
Rasmussen
LH
, …
Lip
GY
. Asymptomatic atrial fibrillation: clinical correlates, management, and outcomes in the EORP‐AF Pilot General Registry. The American Journal of Medicine. 2015;128:509–518.e2.2553442310.1016/j.amjmed.2014.11.0266

Garimella
RS
, 
Chung
EH
, 
Mounsey
JP
, 
Schwartz
JD
, 
Pursell
I
, 
Gehi
AK
. Accuracy of patient perception of their prevailing rhythm: A comparative analysis of monitor data and questionnaire responses in patients with atrial fibrillation. Heart Rhythm. 2015;12:658–665.2559592610.1016/j.hrthm.2015.01.0127

Wong
JA
, 
Conen
D
, 
Van Gelder
I
, 
McIntyre
WF
, 
Crijns
HJ
, 
Wang
J
, …
Healey
JS
. Progression of device‐detected subclinical atrial fibrillation and the risk of heart failure. Journal of the American College of Cardiology. 2018;71:2603–2611.2988011910.1016/j.jacc.2018.03.5198

Wolf
PA
, 
Abbott
RD
, 
Kannel
WB
. Atrial fibrillation as an independent risk factor for stroke: The Framingham Study. Stroke. 1991;22:983–8.186676510.1161/01.str.22.8.9839

Kirchhof
P
, 
Benussi
S
, 
Kotecha
D
, 
Ahlsson
A
, 
Atar
D
, 
Casadei
B
, …
Vardas
P
. 2016 ESC Guidelines for the management of atrial fibrillation developed in collaboration with EACTS: The Task Force for the management of atrial fibrillation of the European Society of Cardiology (ESC) Developed with the special contribution of the European Heart Rhythm Association (EHRA) of the ESC. Endorsed by the European Stroke Organisation (ESO). European Heart Journal. 2016;37:2893–2962. 10.1093/eurheartj/ehw210.2756740810

Orchard
JJ
, 
Neubeck
L
, 
Freedman
B
, 
Webster
R
, 
Patel
A
, 
Gallagher
R
, …
Lowres
N
. Atrial fibrillation screen, management and guideline recommended therapy (AF SMART II) in the rural primary care setting: An implementation study protocol. British Medical Journal Open. 2018;8:e023130. 10.1136/bmjopen-2018-023130.PMC62527583038544411

Friberg
L
, 
Rosenqvist
M
, 
Lindgren
A
, 
Terént
A
, 
Norrving
B
, 
Asplund
K
. High prevalence of atrial fibrillation among patients with ischemic stroke. Stroke. 2014;45:2599–2605. 10.1161/STROKEAHA.114.006070.2503471312

Xiong
Q
, 
Proietti
M
, 
Senoo
K
, 
Lip
GY
. Asymptomatic versus symptomatic atrial fibrillation: A systematic review of age/gender differences and cardiovascular outcomes. International Journal of Cardiology. 2015;191:172–177. 10.1016/j.ijcard.2015.05.011.2597419313

Ding
M
, 
Qiu
C
. Atrial fibrillation, cognitive decline, and dementia: An epidemiologic review. Current Epidemiology Reports. 2018;5:252–261. 10.1007/s40471-018-0159-7.30148041PMC609685414

Friberg
L
, 
Rosenqvist
M
. Less dementia with oral anticoagulation in atrial fibrillation. European Heart Journal. 2018;39:453–460. 10.1093/eurheartj/ehx579.2907784915

Charitos
EI
, 
Stierle
U
, 
Ziegler
PD
, 
Baldewig
M
, 
Robinson
DR
, 
Sievers
HH
, 
Hanke
T
. A comprehensive evaluation of rhythm monitoring strategies for the detection of atrial fibrillation recurrence: Insights from 647 continuously monitored patients and implications for monitoring after therapeutic interventions. Circulation. 2012;126:806–814. 10.1161/CIRCULATIONAHA.112.098079.2282443416

Ramkumar
S
, 
Nerlekar
N
, 
D'Souza
D
, 
Pol
DJ
, 
Kalman
JM
, 
Marwick
TH
. Atrial fibrillation detection using single lead portable electrocardiographic monitoring: A systematic review and meta‐analysis. British Medical Journal Open. 2018;8:e024178. 10.1136/bmjopen-2018-024178.PMC61444873022440417

Berge
T
, 
Lyngbakken
MN
, 
Ihle‐Hansen
H
, 
Brynildsen
J
, 
Pervez
MO
, 
Aagaard
EN
, …
Tveit
A
. Prevalence of atrial fibrillation and cardiovascular risk factors in a 63–65 years old general population cohort: The Akershus Cardiac Examination (ACE) 1950 Study. BMJ Open. 2018;8:e021704.10.1136/bmjopen-2018-021704PMC607462430068617

#### Accuracy

The positive predictive value of an AF event will differ according to pretest probability of AF in a given population (e.g., those with an established diagnosis or one or more risk factors). This is especially relevant to “healthy consumers.” Many technologies to identify AF are readily available directly to those without defined disease and are not deployed as individual or public health interventions. Rather, consumers who possess these technologies, such as smartwatches or smartphone‐connected ECG recorders, opt into the use of these technologies. Therefore, consumer‐driven AF identification is not the same as healthcare‐initiated AF screening. AF identification by these devices requires confirmation, since these AF screening tools have variable specificity (Table 2), raising the potential of a high false‐positive rate in a low prevalence population, and risks of unnecessary treatment.

There have been almost 500 studies assessing accuracy of mHealth devices for AF detection, as described in recent systematic reviews.^16^, ^17^, ^18^ Their capabilities varied according to technologies utilized, settings, and study populations. Two large‐scale screening trials were reported recently (See Section 6).

#### Outcomes

No large outcome trial of screen detected AF and hard endpoints of stroke and death has been conducted as yet.

Although an incidental diagnosis of AF seems to be associated with increased risk of stroke and protection by OAC therapy,^19^, ^20^, ^21^clinical trials to determine any benefit for opportunistically detected AF have not yet been completed but are underway Heartline study https://www.heartline.com).^1^, ^3^, ^9^ This effort addresses the concern that AF detected by screening may identify inherently lower‐risk patients so that efficacy of anticoagulation (and its risk/benefit ratio) requires recalibration. This is necessary prior to issuance of any recommendations. (Currently, no consensus exists yet on how to treat these arrhythmias, even in those with high CHA_2_DS_2_‐VASc scores).

The European and American guidelines do recommend opportunistic screening for early identification of undiagnosed AF in patients aged ≥65 years.^9^, ^22^, ^23^ On the other hand, the U.S. Preventive Services Task Force has presently given an “insufficient” recommendation for systematic screening for AF with electrocardiograms.^131^

#### 3.1.2 Targeted identification in high‐risk individuals

##### Cryptogenic stroke/TIA

Up to one‐third of ischemic strokes is attributed to AF mediated embolism to the brain.^132^ Further, the risk of recurrent thromboembolism is high if AF is left undetected and untreated.^26^, ^27^ Hence, prolonged monitoring for AF poststroke has been recommended in recent guidelines.^9^, ^23^, ^28^ Detection of AF poststroke depends not only on the monitoring device used and the duration of the monitoring period, but also on stroke type and patient selection; thus, the results of AF detection have been heterogenous.^29^, ^30^, ^31^ A meta‐analysis showed that a stepwise approach to AF detection in poststroke patients led to AF detection in 23.7% of patients,^139^ while a combined analysis of two randomized and two observational studies showed a 55% reduction in recurrent stroke following prolonged cardiac monitoring.^129^ However, the optimal AF duration threshold for initiating anticoagulation is currently unknown and may be lower in a poststroke population compared to those with fewer cardiovascular risk factors.^140^

The risk of undiagnosed AF and other sources of thrombi has been considered high in embolic strokes of unknown source (ESUS), prompting studies that evaluated whether empiric NOAC therapy is more effective than antiplatelet therapy without a requirement of AF detection. Two of these studies, NAVIGATE ESUS^141^ and RESPECT‐ESUS,^142^ have not shown a reduction in recurrent stroke in patients receiving NOACs. It should be emphasized that the mere detection of AF after ESUS is not necessarily proof of positive causation. A third study is ongoing, including patients with suggested atrial myopathy (enlarged atria, increased levels of NT‐proBNP, or enlarged P waves).^143^

These findings underscore the need for AF detection prior to initiation of OAC therapy in patients with cryptogenic stroke, ESUS, or ischemic stroke of known origin, and mHealth devices can ease the process of detection.^138^ The threshold of AF burden may very well differ in patients who have had a suspected cardioembolic event and those who have not.^140^

##### Other high‐risk individuals

The key to making AF identification feasible, efficient and clinically valuable is the selection of patients with an increased likelihood of harboring undiagnosed AF, rather than general screening in unselected populations. mHealth ECG recorders can facilitate frequent brief (e.g., 30 seconds) recordings over prolonged periods of time by the very ubiquity of devices (including smartphone‐based apps or watches). These devices are par ticularly well suited to capture intermittent or nonpersistent arrhy thmias; however, it is likely that frequent sampling would be necessar y to capture infrequent parox ysmal AF and even daily “snapshot” ECG monitoring may miss half of AF episodes.^15^, ^37^ AF burden, increasingly recognized as a power ful independent predictor of stroke,^145^ though accurately measured by implanted devices,^146^ cannot be readily calculated from intermittent ECG data. The use of smar twatches with passive intermittent sur veillance using PPG monitoring plus ECG confirmation may be a more effective screening tool and is currently being evaluated (Heartline study https://www.heartline.com).

Formal screening with mHealth ECG recordings has yielded meaningful incidences of newly diagnosed AF, statistically greater than if diagnosis relied only on the office ECG (Table 3). The yield generally is enhanced by the presence of risk factors, such as older age and higher CHA_2_DS_2_‐VASc scores. Several studies^40^, ^41^, ^42^ screened untargeted populations, and all yielded new AF diagnoses at a rate under 1%. By focusing on older patients (75‐76 years of age) at greater risk, Swedish studies identified new AF in 3% of study participants, and up to 7.4% when additional risk factors beyond age were required.^1^, ^2^, ^3^ Lowres et al.^125^ in a patient level meta‐nalysis found that new AF detection rate increased progressively with age from 0.34% for <60 years to 2.73% ≥85 years. Importantly, the number of subjects needed to screen to discover AF meeting indications for anticoagulation was 1089 for subjects <60 years but 83 ≥65 years.

#### 3.1.3 Diagnostics in people with established atrial fibrillation

mHealth has important implications for the care of those already diagnosed with AF. Several key characteristics of AF can be measured with long‐term continuous or near‐continuous monitoring, and the information gained may provide valuable information for patient management.

Furthermore, while several studies succeeded in establishing the sensitivity and specificity of novel devices for the detection of AF, no study to date has yet evaluated the utility of an mHealth intervention in affecting clinical outcomes. The iPhone Helping Evaluate Atrial Fibrillation Rhythm through Technology (iHEART), a single‐center, prospective, randomized controlled trial, and the Heartline study seek to accomplish this goal (https://www.heartline.com).^43^, ^44^

#### 3.1.4 Atrial fibrillation therapy

##### Atrial fibrillation burden

Current guidelines for anticoagulation are based principally on the presence of risk factors and a diagnosis of clinical AF, regardless of AF duration, symptomatology, or burden.^130^ This applies even if the AF has been quiescent for long periods or eliminated altogether as the result of rhythm control interventions including antiarrhythmic drugs, ablation, or risk factor modification.^130^ However, there is increasing recognition that AF burden matters; for example, paroxysmal events have less thromboembolic risk than persistent AF^145^ This understanding has been extended during continuous monitoring from CIEDs which depict AF with high granularity, and first advanced the metrics of “AF days” and burden in terms of cumulative load (hours/day) and concentration (density of AF days).^146^ This measure is likely to be important for understanding mHealth discovered AF.

##### CIEDS

AF burden can be characterized as %/time monitored, longest duration, and density. Retrieved data provide an insight into natural history and associated sequelae.^33^, ^39^, ^45^, ^46^ This led to oral anticoagulation intervention trials to determine the ability to reduce stroke on the basis of AF duration.^47^, ^48^ These suggest that a threshold exists below which the risk of thromboembolic stroke is low and risk–benefit ratio may not justify chronic administration of oral anticoagulants. For instance, CIED data indicate that short subclinical AF events have lesser risk than more prolonged (and therefore more likely to be symptomatic) events.^156^ Device‐detected, “subclinical” atrial high‐rate episodes (AHRE) lasting 6 minutes to 24 hours are associated with increased stroke risk, but the absolute risk is considerably lower than expected based on risk factors alone.^45^, ^46^, ^50^ Whether these require anticoagulation in high‐risk individuals is the subject of ongoing studies.^47^, ^48^, ^51^ Importantly, very short AF episodes (episodes in which both the onset and offset of AT/AF were present within a single EGM recording) were not associated with adverse outcomes^159^ which may be important for mHealth monitoring.

##### mHealth

AF detection using digital health tools offers further insights in patients without indication for implantable devices. mHealth extends AF screening to younger patients without cardiovascular disease and thromboembolic potential may be low. Those with high AF burden (defined by ≥11.4%; mean duration 11.7 hours) detected on a 14‐day patch monitor had an increased thromboembolic event rate compared to those with lower AF burdens.^160^ There remains significant treatment variation in use of OAC, especially for device‐detected AF.^161^ This may be due to a large clinical uncertainty regarding the optimal cutpoint, even though observational data indicate that OAC is associated with a decreased risk of stroke for episodes >24 hours and possibly for episodes 6‐24 hours.^161^

Currently, there are no prospectively validated cutpoints or risk models that incorporate AF burden into decision‐making for stroke prevention therapies.

Key knowledge gap:

- Identify characteristics (duration, episode number/density) and risk factors that justify anticoagulation for mHealth detected AF.

##### Rhythm and rate control

- *Rhythm* While we await data on OAC treatment for mHealth detected AF, the finding of the arrhythmia should initiate mHealth monitoring of NSR retention, QT intervals (important for those on some antiarrhythmic drugs),^162^ and discussion of cardiovascular risk factor modification and lifestyle changes, since AF coexists with comorbidities that may influence its occurrence and natural history (See Section 4). Thus, alcohol reduction, treatment of OSA, moderate exercise, and weight loss have been shown to reduce AF burden.^56^, ^57^, ^58^, ^59^
- *Rate* While the primary goal of rate control is to minimize AF‐related symptoms, prolonged tachycardia can result in effort intolerance and/or tachycardia‐mediated cardiomyopathy while excessively low heart rate targets may increase the risk of bradyarrhythmias that result in symptoms and device implantation. The European Society of Cardiology recommends lenient resting heart rate targets (<100‐110), whereas the ACC/AHA/HRS guidelines recommend a target rate of <80 bpm. Often these targets are tailored to the individual patient based on symptoms and presence or propensity for HF. mHealth technologies can be used to assess ventricular rates during AF over long time periods and evaluate the effects of rate‐control therapies.^9^, ^23^

### 3.2 Sudden cardiac death

See also section 4.1 Ischemia heart disease.

#### Ventricular arrhythmias

The use of mHealth technology to diagnose ventricular arrhythmias lags behind its application to AF (See Section 3.1). Detection of symptomatic VT has been reported using the AliveCor cardiac monitor (AliveCor, San Francisco, USA) and SmartWatch.^60^, ^61^ Sophisticated automated analysis of a 2‐minute PPG recording by the camera of a commercially available smartphone (iPhone 4S, Apple) can distinguish between AF, PACs, and PVCs from sinus rhythm, with a sensitivity of 0.733 and specificity of 0.976 for PVCs.^62^, ^63^ PVCs may challenge to PPG‐based systems, as many PVCs are nonperfusing.^171^ An ECG tracing is therefore essential in order to facilitate rhythm diagnosis and avoid misclassification of “slow PPG pulse rates” (bradysphygmia) simply as “bradycardia.”

#### Syncope

Syncope presents unique challenges for mHealth applications. While prolonged ambulatory monitoring using medical‐grade devices (wearable and implantable) has been the mainstay of cardiac rhythm diagnosis during episodes of syncope, user‐activated systems must either be activated by the patient during prodromal symptoms (if present and time permits) in anticipation of syncope, or else incorporate loop recording to allow postsyncope activation.^90^ This capability is not incorporated in currently popular consumer‐grade wearable devices. However, a randomized controlled trial of AliveCor versus usual care in participants presenting with palpitations or presyncope showed a faster and increased rate of detection of symptomatic arrhythmias in the intervention group, suggesting that at least in presyncope, patient‐activated rhythm detection using a commercially available mHealth device is productive.^172^ Rhythms reported by devices that rely on heart rates will likely require validation with a medical‐grade system to provide an ECG tracing during an event to allow determination of the causative rhythm.

There is a significant overlap between transient loss of consciousness and mechanical falls due to orthostatic intolerance, neurologic, or orthopedic problems. This is particularly disabling in elderly subjects and often unwitnessed.^66^, ^67^ Mobile applications that combine analysis of heart rate monitoring together with fall detection, GPS positioning, video recording with display of patients' surroundings, and the capability to send alerts either triggered by patients in case of symptoms or automatically in case of detected falls, may be useful.

#### Cardiac arrest

The detection and response to sudden cardiac arrest (SCA) is an area where mHealth applications may prove lifesaving. As rapid treatment for cardiac arrest has consistently been associated with improved survival, pre‐emptive identification of at‐risk persons, detection of cardiac arrests, alerting of nearby lay and professional first responders, and coaching or quality assurance in the performance of cardiopulmonary resuscitation (CPR) are ideally suited to the mHealth paradigm in societies where mobile smartphones are ubiquitous.

#### Prediction

It is possible that mHealth devices which continuously monitor heart rhythm and other physiologic data may be able to better predict impending SCA, even using measures which have not shown sufficient specificity or sensitivity when measured intermittently, such as heart rate variability.^175^ However, such continuous monitoring is present already in CIEDs and has not yet proven to be sufficiently predictive to be clinically useful.^176^ Therefore, the prediction of SCA by mHealth devices, while a tantalizing prospect, remains to be realized.

#### Notification and reaction

Once cardiac arrest occurs, rapid identification is essential to trigger a response by emergency responders. Wearable devices that combine physiologic monitoring, GPS, and a method of communication with emergency services such as cellular service are well positioned to provide almost instantaneous alert as well as location information.^70^, ^71^ An early device using a piezoelectric sensor to detect the pulse was capable of transmitting an alert to emergency medical system or other responders when a pulse was not detected and the watch (and thus the wearer) was still.^179^ Preliminary reports indicate that smart speakers in commodity smart devices may be able to identify agonal breath patterns for sudden cardiac death detection.^180^ Widespread diffusion of such technology to patients at elevated risk of SCA will be necessary before any potential benefits can be tested.

The ubiquity of mobile phones in society leads to more rapid notification of emergency services, and the possibility of a dispatcher gathering information from a bystander at the patient’s side and delivering instructions on care, such as CPR. This was associated with improved outcomes for a variety of emergencies.^181^ Notification of lay first responders in the vicinity of a cardiac arrest is also feasible with current technology. A blinded, randomized trial conducted in Stockholm, Sweden, demonstrated that such a system improved the rate of bystander CPR.^182^ However, almost 10,000 volunteers were recruited over approximately 18 months, during which 667 activations occurred, emphasizing the large resources needed and the low rate of utilization of trained volunteers, even when alerted by mobile phone.

Whether a trained or novice bystander responds, mobile devices may be further useful to provide voice (or video) instructions from a dispatcher or from the device itself. Studies of prerecorded audio, live video, and animation‐based instruction have shown improvements in some aspects of CPR delivery and AED use, although technology continues to evolve.^76^, ^77^, ^78^, ^79^ One limitation is that as such apps are unregulated, many do not convey current basic life support algorithms and may have poor usability.^187^ In addition, delay in commencing CPR and in calling emergency services due to distraction of the rescuer by using an app is a concern.^188^

Automated external defibrillator (AED) use in cardiac arrest is associated with improved survival, but AED use remains low.^189^ Mobile devices have the potential to increase this by assisting with the retrieval and use of AEDs. Multiple apps have been created to locate AEDs in the vicinity of the user, although with mixed results in simulations.^83^, ^84^, ^85^ Barriers include the accuracy of AED location databases, size of the user base, app interface, and the availability of multiple apps instead of a single validated regional, national, or international standard. An emerging approach to circumvent these limitations is the dispatch of an AED via a drone to the location of the cardiac arrest, which is expected to reduce time to defibrillation, especially in rural areas.^193^ Feasibility has been demonstrated.^194^

#### 3.2.1 Clinical trial

The complete chain from activation of citizen responders was tested in the Heartrunner trial^195^ in a region of almost 2 million inhabitants. Results showed that citizen responders arrived before emergency services 42% of out of hospital cardiac arrests, accompanied by a threefold increase in bystander defibrillation with a trend to improved 30‐day survival. Results were more pronounced when emergency arrival times were longer, for example, in rural areas.

REFERENCES SECTION 31

Svennberg
E
, 
Engdahl
J
, 
Al‐Khalili
F
, 
Friberg
L
, 
Frykman
V
, 
Rosenqvist
M
. Mass screening for untreated atrial fibrillation: The STROKESTOP study. Circulation. 2015;131:2176–2184.2591080010.1161/CIRCULATIONAHA.114.0143432

Engdahl
J
, 
Andersson
L
, 
Mirskaya
M
, 
Rosenqvist
M
. Stepwise screening of atrial fibrillation in a 75‐year‐old population: Implications for stroke prevention. Circulation. 2013;127:930–937.2334356410.1161/CIRCULATIONAHA.112.1266563

Gudmundsdottir
K
, 
Fredriksson
T
, 
Svennberg
E
, 
Al‐Khalili
F
, 
Friberg
L
, 
Frykman
V
, …
Engdahl
J
. Stepwise mass screening for atrial fibrillation using N‐terminal B‐type natriuretic peptide: The STROKESTOP II study. Europace. 2020;22:24–32. 10.1093/europace/euz255.31790147PMC69450544

Doliwa
R
, 
Sobocinski
P
, 
Anggårdh
RE
, 
Frykman Kull
V
, 
von Arbin
M
, 
Wallén
H
, 
Rosenqvist
M
. Improved screening for silent atrial fibrillation after ischaemic stroke. Europace. 2012;2012(14):1112–1116. 10.1093/europace/eur431.223080865

Tieleman
RG
, 
Plantinga
Y
, 
Rinkes
D
, 
Bartels
GL
, 
Posma
JL
, 
Cator
R
, 
Hofman
C
, 
Houben
RP
. Validation and clinical use of a novel diagnostic device for screening of atrial fibrillation. Europace. 2014;16:1291–1295.2482576610.1093/europace/euu057PMC41496086

Kaasenbrood
F
, 
Hollander
M
, 
de Bruijn
SHM
, 
Dolmans
CPE
, 
Tieleman
RG
, 
Hoes
AW
, 
Rutten
FH
. Opportunistic screening versus usual care for diagnosing atrial fibrillation in general practice: A cluster randomised controlled trial. British Journal of General Practice. 2020;70(695):e427–e433. 10.3399/bjgp20X708161.PMC6988680319880847

Tavernier
R
, 
Wolf
M
, 
Kataria
V
, 
Phlips
T
, 
Huys
R
, 
Taghji
P
, …
Duytschaever
M
. Screening for atrial fibrillation in hospitalised geriatric patients. Heart. 2018;104:588–593. 10.1136/heartjnl-2017-311981.288830328

Turakhia
MP
, 
Ullal
AJ
, 
Hoang
DD
, 
Than
CT
, 
Miller
JD
, 
Friday
KJ
, …
Heidenreich
PA
. Feasibility of extended ambulatory electrocardiogram monitoring to identify silent atrial fibrillation in high‐risk patients: The Screening Study for Undiagnosed Atrial Fibrillation (STUDY‐AF). Clinical Cardiology. 2015;2015(38):285–292. 10.1002/clc.22387.PMC4654330258734769

Steinhubl
SR
, 
Waalen
J
, 
Edwards
AM
, 
Ariniello
LM
, 
Mehta
RR
, 
Ebner
GS
, …
Topol
EJ
. Effect of a home‐based wearable continuous ECG Monitoring patch on detection of undiagnosed atrial fibrillation: The mSToPS randomized clinical trial. Journal of the American Medical Association. 2018;320:146–155.2999833610.1001/jama.2018.8102PMC658351810

Rooney
MR
, 
Soliman
EZ
, 
Lutsey
PL
, 
Norby
FL
, 
Loehr
LR
, 
Mosley
TH
, …
Chen
LY
. Prevalence and characteristics of subclinical atrial fibrillation in a community‐dwelling elderly population. Circulation: Arrhythmia and Electrophysiology. 2019;12(10):e007390. 10.1161/CIRCEP.119.007390.31607148PMC681438711

Heckbert
SR
, 
Austin
TR
, 
Jensen
PN
, 
Floyd
JS
, 
Psaty
BM
, 
Soliman
EZ
, 
Kronmal
RA
. Yield and consistency of arrhythmia detection with patch electrocardiographic monitoring: The Multi‐Ethnic Study of Atherosclerosis. Journal of Electrocardiology. 2018;51:997–1002. 10.1016/j.jelectrocard.2018.07.027.30497763PMC627860812

Lowres
N
, 
Neubeck
L
, 
Salkeld
G
, 
Krass
I
, 
McLachlan
AJ
, 
Redfern
J
, …
Freedman
SB
. Feasibility and cost‐effectiveness of stroke prevention through community screening for atrial fibrillation using iPhone ECG in pharmacies. The SEARCH‐AF study. Thrombosis & Haemostasis. 2014;111:1167–1176. 10.1160/th14-03-0231.2468708113

Chan
PH
, 
Wong
CK
, 
Poh
YC
, 
Pun
L
, 
Leung
WW
, 
Wong
YF
, 
Siu
CW
. Diagnostic performance of a smartphone‐based photoplethysmographic application for atrial fibrillation screening in a primary care setting. Journal of the American Heart Association. 2016;5(7):e003428. 10.1161/JAHA.116.003428.27444506PMC501537914

Halcox
JPJ
, 
Wareham
K
, 
Cardew
A
, 
Gilmore
M
, 
Barry
JP
, 
Phillips
C
, 
Gravenor
MB
. Assessment of Remote heart rhythm sampling using the alivecor heart monitor to screen for atrial fibrillation: The REHEARSE‐AF Study. Circulation. 2017;136:1784–1794. 10.1161/circulationaha.117.030583.2885172915

Perez
MV
, 
Mahaffey
KW
, 
Hedlin
H
, 
Rumsfeld
JS
, 
Garcia
A
, 
Ferris
T
, …
Turakhia
MP
. Large‐scale assessment of a smartwatch to identify atrial fibrillation. New England Journal of Medicine. 2019;381:1909–1917. 10.1056/NEJMoa1901183.PMC81126053172215116

Giebel
GD
, 
Gissel
C
. Accuracy of mHealth devices for atrial fibrillation screening: Systematic review. JMIR Mhealth Uhealth. 2019;7:e13641. 10.2196/13641.31199337PMC659842217

Lowres
N
, 
Olivier
J
, 
Chao
TF
, 
Chen
SA
, 
Chen
Y
, 
Diederichsen
A
, …
Freedman
B
. Estimated stroke risk, yield, and number needed to screen for atrial fibrillation detected through single time screening: A multicountry patient‐level meta‐analysis of 141,220 screened individuals. PLoS Medicine. 2019;16:e1002903. 10.1371/journal.pmed.1002903.31553733PMC676076618

O'Sullivan
JW
, 
Grigg
S
, 
Crawford
W
, 
Turakhia
MP
, 
Perez
M
, 
Ingelsson
E
, …
Ashley
EA
. Accuracy of smartphone camera applications for detecting atrial fibrillation: A systematic review and meta‐analysis. Journal of the American Medical Association Netw Open. 2020;3:e202064. 10.1001/jamanetworkopen.2020.2064.PMC71254333224290819

Freedman
B
, 
Potpara
TS
, 
Lip
GY
. Stroke prevention in atrial fibrillation. Lancet. 2016;388:806–817. 10.1016/S0140-6736(16)31257-0.2756027620

Martinez
C
, 
Katholing
A
, 
Freedman
SB
. Adverse prognosis of incidentally detected ambulatory atrial fibrillation. A cohort study. Thrombosis and Haemostasis. 2014;112:276–286.2495305110.1160/TH4-04-0383PMC637498321

Tsivgoulis
G
, 
Katsanos
AH
, 
Köhrmann
M
, 
Caso
V
, 
Perren
F
, 
Palaiodimou
L
, …
Alexandrov
AV
. Duration of implantable cardiac monitoring and detection of atrial fibrillation in ischemic stroke patients: A systematic review and meta‐analysis. Journal of Stroke. 2019;21:302–311. 10.5853/jos.2019.01067.31590474PMC678001822

Freedman
B
, 
Camm
J
, 
Calkins
H
, 
Healey
JS
, 
Rosenqvist
M
, 
Wang
J
, …
Yan
BP
. A report of the AF‐SCREEN international collaboration. Circulation. 2017;135:1851–1867.2848383210.1161/CIRCULATIONAHA.116.02669323

January
CT
, 
Wann
LS
, 
Calkins
H
, 
Chen
LY
, 
Cigarroa
JE
, 
Cleveland
JC
, …
Yancy
CW
. 2019 AHA/ACC/HRS focused update of the 2014 AHA/ACC/HRS guideline for the management of patients with atrial fibrillation: A report of the American College of Cardiology/American Heart Association Task Force on Clinical Practice Guidelines and the Heart Rhythm Society in collaboration with the Society of Thoracic Surgeons. Circulation. 2019;140:e125–e151. 10.1161/CIR.0000000000000665.3068604124

Jonas
DE
, 
Kahwati
LC
, 
Yun
JDY
, 
Middleton
JC
, 
Coker‐Schwimmer
M
, 
Asher
GN
. Screening for atrial fibrillation with electrocardiography: Evidence report and systematic review for the US preventive services task force. Journal of the American Medical Association. 2018;320:485–498. 10.1001/jama.2018.4190.3008801525

Hannon
N
, 
Sheehan
O
, 
Kelly
L
, 
Marnane
M
, 
Merwic
KA
, 
Moore
A
, …
Kelly
PJ
. Stroke associated with atrial fibrillation–incidence and early outcomes in the north Dublin population stroke study. Cerebrovascular Diseases. 2010;29:43–49. 10.1159/000255973.19893311PMC291440126

Furie
KL
, 
Goldstein
LB
, 
Albers
GW
, 
Khatri
P
, 
Neyens
R
, 
Turakhia
MP
, 
Wood
KA
. American Heart Association Stroke Council; Council on Quality of Care and Outcomes Research; Council on Cardiovascular Nursing; Council on Clinical Cardiology; Council on Peripheral Vascular Disease. Oral antithrombotic agents for the prevention of stroke in nonvalvular atrial fibrillation: A science advisory for healthcare professionals from the American Heart Association/American Stroke Association. Stroke. 2012;43:3442–53. 10.1161/STR.0b013e318266722a.2285872827

Kolominsky‐Rabas
PL
, 
Weber
M
, 
Gefeller
O
, 
Neundoerfe
RB
, 
Heuschmann
PU
. Epidemiology of ischemic stroke subtypes according to TOAST criteria: Incidence, recurrence, and long‐term survival in ischemic stroke subtypes: A population‐based study. Stroke. 2001;32:2735–2740.1173996510.1161/hs1201.10020928

Schnabel
RB
, 
Haeusler
KG
, 
Healey
JS
, 
Freedman
B
, 
Boriani
G
, 
Brachmann
J
, …
Yan
B
. Searching for atrial fibrillation post‐stroke: A white paper of the Af‐SCREEN international collaboration. Circulation. 2019;140:1834–1850. 10.1161/CIRCULATIONAHA.119.040267.3176526129

Kishore
A
, 
Vail
A
, 
Majid
A
, 
Dawson
J
, 
Lees
KR
, 
Tyrrell
PJ
, 
Smith
CJ
. Detection of atrial fibrillation after ischemic stroke or transient ischemic attack: A systematic review and meta‐analysis. Stroke. 2014;45:520–526. 10.1161/STROKEAHA.113.003433.2438527530

Sanna
T
, 
Diener
HC
, 
Passman
RS
, 
Di Lazzaro
V
, 
Bernstein
RA
, 
Morillo
CA
, …
Brachmann
J
. CRYSTAL AF Investigators. Cryptogenic stroke and underlying atrial fibrillation. New England Journal of Medicine. 2014;370:2478–2486. 10.1056/NEJMoa1313600.2496356731

Zungsontiporn
N
, 
Link
MS
. Newer technologies for detection of atrial fibrillation. British Medical Journal. 2018;363:k3946. 10.1136/bmj.k3946.3033310532

Sposato
LA
, 
Cipriano
LE
, 
Saposnik
G
, 
Ruíz Vargas
E
, 
Riccio
PM
, 
Hachinski
V
. Diagnosis of atrial fibrillation after stroke and transient ischaemic attack: A systematic review and meta‐analysis. Lancet Neurology. 2015;14:377–387. 10.1016/S1474-4422(15)70027-X.2574810233

Kaplan
RM
, 
Koehler
J
, 
Ziegler
PD
, 
Sarkar
S
, 
Zweibel
S
, 
Passman
RS
. Stroke risk as a function of atrial fibrillation duration and CHA2DS2‐VASc score. Circulation. 2019;140:1639–1646. 10.1161/CIRCULATIONAHA.119.041303.3156412634

Hart
RG
, 
Sharma
M
, 
Mundl
H
, 
Kasner
SE
, 
Bangdiwala
SI
, 
Berkowitz
SD
, …
Connolly
SJ
. NAVIGATE ESUS Investigators. New England Journal of Medicine. 2018;378:2191–2201. 10.1056/NEJMoa1802686.35

Diener
HC
, 
Sacco
RL
, 
Easton
JD
, 
Granger
CB
, 
Bernstein
RA
, 
Uchiyama
S
, …
Toyoda
K
. RE‐SPECT ESUS Steering Committee and Investigators. Dabigatran for prevention of stroke after embolic stroke of undetermined source. New England Journal of Medicine. 2019;380:1906–1917. 10.1056/NEJMoa1813959.3109137236

Kamel
H
, 
Longstreth
WT
, 
Tirschwell
DL
, 
Kronmal
RA
, 
Broderick
JP
, 
Palesch
YY
, …
Elkind
MS
. The AtRial cardiopathy and antithrombotic drugs in prevention after cryptogenic stroke randomized trial: Rationale and methods. International Journal of Stroke. 2019;14:207–214. 10.1177/1747493018799981.30196789PMC664538037

Yano
Y
, 
Greenland
P
, 
Lloyd‐Jones
DM
, 
Daoud
EG
, 
Koehler
JL
, 
Ziegler
PD
. Simulation of daily snapshot rhythm monitoring to identify atrial fibrillation in continuously monitored patients with stroke risk factors. PLoS One. 2016;11:e0148914. 10.1371/journal.pone.0148914.26882334PMC475552938

Chen
LY
, 
Chung
MK
, 
Allen
LA
, 
Ezekowitz
M
, 
Furie
KL
, 
McCabe
P
, …
Turakhia
MP
. American Heart Association Council on Clinical Cardiology; Council on Cardiovascular and Stroke Nursing; Council on Quality of Care and Outcomes Research; and Stroke Council. Atrial fibrillation burden: Moving beyond atrial fibrillation as a binary entity: A scientific statement from the American Heart Association. Circulation. 2018;137:e623–e644. 10.1161/CIR.0000000000000568.29661944PMC846325839

Varma
N
, 
Stambler
B
, 
Chun
S
. Detection of atrial fibrillation by implanted devices with wireless data transmission capability. Pacing and Clinical Electrophysiology. 2005;28(Suppl 1):S133–S136.1568348010.1111/j.1540-8159.2005.00083.x40

Chan
NY
, 
Choy
CC
. Screening for atrial fibrillation in 13 122 Hong Kong citizens with smartphone electrocardiogram. Heart. 2017;103:24–31. 10.1136/heartjnl-2016-309993.2773353341

Chan
PH
, 
Wong
CK
, 
Pun
L
, 
Wong
YF
, 
Wong
MM
, 
Chu
DW
, 
Siu
CW
. Head‐to‐head comparison of the alivecor heart monitor and microlife WatchBP office AFIB for atrial fibrillation screening in a primary care setting. Circulation. 2017;135:110–112. 10.1161/CIRCULATIONAHA.116.024439.2802806642

Proietti
M
, 
Mairesse
GH
, 
Goethals
P
, 
Scavee
C
, 
Vijgen
J
, 
Blankoff
I
, …
Lip
GY
. Belgian Heart Rhythm Week Investigators. A population screening programme for atrial fibrillation: A report from the Belgian Heart Rhythm Week screening programme. Europace. 2016;18:1779–1786. 10.1093/europace/euw069.2717000043

Caceres
BA
, 
Hickey
KT
, 
Bakken
SB
, 
Biviano
AB
, 
Garan
H
, 
Goldenthal
IL
, …
Jia
H
. Mobile electrocardiogram monitoring and health‐related quality of life in patients with atrial fibrillation: Findings from the iPhone helping evaluate atrial fibrillation rhythm through technology (iHEART) study. The Journal of Cardiovascular Nursing. 2020;35(4):327–336. 10.1097/JCN.0000000000000646.32015256PMC729973944

Hickey
KT
, 
Hauser
NR
, 
Valente
LE
, 
Riga
TC
, 
Frulla
AP
, 
Creber
MR
, …
Wang
DY
. A single‐center randomized, controlled trial investigating the efficacy of a mHealth ECG technology intervention to improve the detection of atrial fibrillation: The iHEART study protocol. BMC Cardiovascular Disorders. 2016;16:152. 10.1186/s12872-016-0327-y.27422639PMC494729945

Healey
JS
, 
Connolly
SJ
, 
Gold
MR
, 
Israel
CW
, 
Van Gelder
IC
, 
Capucci
A
, …
Hohnloser
SH
. Subclinical atrial fibrillation and the risk of stroke. New England Journal of Medicine. 2012;366:120–129.10.1056/NEJMoa11055752223622246

Van Gelder
IC
, 
Healey
JS
, 
Crijns
H
, 
Wang
J
, 
Hohnloser
SH
, 
Gold
MR
, 
Connolly
SJ
. Duration of device‐detected subclinical atrial fibrillation and occurrence of stroke in ASSERT. European Heart Journal. 2017;38:1339–1344.2832913910.1093/eurheartj/ehx04247

Martin
DT
, 
Bersohn
MM
, 
Waldo
AL
, 
Wathen
MS
, 
Choucair
WK
, 
Lip
GY
, …
Halperin
JL
. IMPACT investigators randomized trial of atrial arrhythmia monitoring to guide anticoagulation in patients with implanted defibrillator and cardiac resynchronization devices. European Heart Journal. 2015;36:1660–1668.2590877410.1093/eurheartj/ehv11548

Lopes
RD
, 
Alings
M
, 
Connolly
SJ
, 
Beresh
H
, 
Granger
CB
, 
Mazuecos
JB
, …
Healey
JS
. Rationale and design of the apixaban for the reduction of thrombo‐embolism in patients with device‐detected sub‐clinical atrial fibrillation (ARTESiA) trial. American Heart Journal. 2017;189:137–145.2862537010.1016/j.ahj.2017.04.00849

Al‐Turki
A
, 
Marafi
M
, 
Russo
V
, 
Proietti
R
, 
Essebag
V
. Subclinical atrial fibrillation and risk of stroke: Past, present and future. Medicina. 2019;55:611. 10.3390/medicina55100611.PMC68433293154707850

Glotzer
TV
, 
Hellkamp
AS
, 
Zimmerman
J
, 
Sweeney
MO
, 
Yee
R
, 
Marinchak
R
, …
Lamas
GA
, 
Investigators
MOST
. Atrial high rate episodes detected by pacemaker diagnostics predict death and stroke: Report of the atrial diagnostics ancillary study of the MOde selection trial (MOST). Circulation. 2003;107:1614–1619.1266849510.1161/01.CIR.0000057981.70380.4551

Kirchhof
P
, 
Blank
BF
, 
Calvert
M
, 
Camm
AJ
, 
Chlouverakis
G
, 
Diener
HC
, …
Vardas
P
. Probing oral anticoagulation in patients with atrial high rate episodes: Rationale and design of the non‐vitamin K antagonist Oral anticoagulants in patients with Atrial High rate episodes (NOAH‐AFNET 6) trial. American Heart Journal. 2017;190:12–18.2876020510.1016/j.ahj.2017.04.015PMC554617452

Swiryn
S
, 
Orlov
MV
, 
Benditt
DG
, 
DiMarco
JP
, 
Lloyd‐Jones
D
, 
Karst
E
, …
Waldo
AL
. Clinical implications of brief device‐detected atrial tachyarrhythmias in a cardiac rhythm management device population: Results from the registry of atrial tachycardia and atrial fibrillation episodes. Circulation. 2016;134:1130–1140. 10.1161/CIRCULATIONAHA.115.020252.2775494653

Go
AS
, 
Reynolds
K
, 
Yang
J
, 
Gupta
N
, 
Lenane
J
, 
Sung
SH
, …
Solomon
MD
. Association of burden of atrial fibrillation with risk of ischemic stroke in adults with paroxysmal atrial fibrillation: The KP‐RHYTHM study. Journal of the American Medical Association Cardiology. 2018;3:601–608.2979994210.1001/jamacardio.2018.1176PMC614566354

Perino
AC
, 
Fan
J
, 
Askari
M
, 
Heidenreich
PA
, 
Keung
E
, 
Raitt
MH
, …
Turakhia
MP
. Practice variation in anticoagulation prescription and outcomes after device‐detected atrial fibrillation. Circulation. 2019;139:2502–2512. 10.1161/CIRCULATIONAHA.118.038988.30880434PMC665219155

Garabelli
P
, 
Stavrakis
S
, 
Albert
M
, 
Koomson
E
, 
Parwani
P
, 
Chohan
J
, …
Po
S
. Comparison of QT interval readings in normal sinus rhythm between a smartphone heart Monitor and a 12‐lead ECG for healthy volunteers and inpatients receiving sotalol or dofetilide. Cardiovascular Electrophysiology. 2016;27:827–32.10.1111/jce.129762702765356

Congrete
S
, 
Bintvihok
M
, 
Thongprayoon
C
, 
Bathini
T
, 
Boonpheng
B
, 
Sharma
K
, …
Cheungpasitporn
W
. Effect of obstructive sleep apnea and its treatment of atrial fibrillation recurrence after radiofrequency catheter ablation: A meta‐analysis. Journal of Evidence‐Based Medicine. 2018;11:145–151. 10.1111/jebm.12313.3009130157

Kanagala
R
, 
Murali
NS
, 
Friedman
PA
, 
Ammash
NM
, 
Gersh
BJ
, 
Ballman
KV
, …
Somers
VK
. Obstructive sleep apnea and the recurrence of atrial fibrillation. Circulation. 2003;107:2589–2594.1274300210.1161/01.CIR.0000068337.25994.2158

Pathak
RK
, 
Middeldorp
ME
, 
Meredith
M
, 
Mehta
AB
, 
Mahajan
R
, 
Wong
CX
, …
Sanders
P
. Long‐term effect of goal‐directed weight management in an atrial fibrillation cohort: A long‐term follow‐up study (LEGACY). Journal of the American College of Cardiology. 2015;65:2159–69. 10.1016/j.jacc.2015.03.002.2579236159

Voskoboinik
A
, 
Kalman
JM
, 
De Silva
A
, 
Nicholls
T
, 
Costello
B
, 
Nanayakkara
S
, …
Kistler
PM
. Alcohol abstinence in drinkers with atrial fibrillation. New England Journal of Medicine. 2020;382:20–28. 10.1056/NEJMoa181759.3189351360

Ringwald
M
, 
Crich
A
, 
Beysard
N
. Smart watch recording of ventricular tachycardia: Case study. The American Journal of Emergency Medicine. 2019;38(4):849.e3–849.e5.10.1016/j.ajem.2019.10.0403178597361

Waks
JW
, 
Fein
AS
, 
Das
S
. Wide complex tachycardia recorded with a smartphone cardiac rhythm monitor. Journal of the American Medical Association Internal Medicine. 2015;175:437–439.2562188010.1001/jamainternmed.2014.758662

Chong
JW
, 
Esa
N
, 
McManus
DD
, 
Chon
KH
. Arrhythmia discrimination using a smart phone. IEEE Journal of Biomedical and Health Informatics. 2018;19:815–24.10.1109/JBHI.2015.2418195PMC65997132583853063

McManus
DD
, 
Chong
JW
, 
Soni
A
, 
Saczynski
JS
, 
Esa
N
, 
Napolitano
C
, …
Chon
KH
. PULSESMART: Pulse‐based arrhythmia discrimination using a novel smartphone application. Journal of Cardiovascular Electrophysiology. 2016;27:51–57.2639172810.1111/jce.12842PMC476831064

Billet
S
, 
Rollin
A
, 
Mondoly
P
, 
Monteil
B
, 
Fournier
P
, 
Cariou
E
, …
Maury
P
. Hemodynamic consequences of premature ventricular contractions: Association of mechanical bradycardia and postextrasystolic potentiation with premature ventricular contraction‐induced cardiomyopathy. Heart Rhythm. 2019;16:853–860.3055083510.1016/j.hrthm.2018.12.00865

Reed
MJ
, 
Grubb
NR
, 
Lang
CC
, 
O’Brien
R
, 
Simpson
K
, 
Padarenga
M
, …
Coats
T
. Multi‐centre randomised controlled trial of a smartphone‐based event recorder alongside standard care versus standard care for patients presenting to the emergency department with palpitations and pre‐syncope: The IPED (Investigation of Palpitations in the ED) study. EClinicalMedicine. 2019;8:37–46.3119363610.1016/j.eclinm.2019.02.005PMC653755566

Davis
JC
, 
Robertson
MC
, 
Ashe
MC
, 
Liu‐Ambrose
T
, 
Khan
KM
, 
Marra
CA
. International comparison of cost of falls in older adults living in the community: A systematic review. Osteoporosis International. 2010;21:1295–306.2019584610.1007/s00198-009-1162-067

Heinrich
S
, 
Rapp
K
, 
Rissmann
U
, 
Becker
C
, 
König
HH
. Cost of falls in old age: A systematic review. Osteoporosis International. 2010;21:891–902.1992449610.1007/s00198-009-1100-168

Lee
H
, 
Shin
SY
, 
Seo
M
, 
Nam
GB
, 
Joo
S
. Prediction of ventricular tachycardia one hour before occurrence using artificial neural networks. Scientific Reports. 2016;6:32390.2756132110.1038/srep32390PMC499995269

Au‐Yeung
WM
, 
Reinhall
PG
, 
Bardy
GH
, 
Brunton
SL
. Development and validation of warning system of ventricular tachyarrhythmia in patients with heart failure with heart rate variability data. PloS One. 2018;13:e0207215.3042788010.1371/journal.pone.0207215PMC623535870

Kwon
JM
, 
Lee
Y
, 
Lee
Y
, 
Lee
S
, 
Park
J
. An algorithm based on deep learning for predicting in‐hospital cardiac arrest. Journal of the American Heart Association. 2018;7:e008678. 10.1161/JAHA.118.008678.29945914PMC606491171

Praveen Kumar
D
, 
Amgoth
T
, 
Annavarapu
CSR
. Machine learning algorithms for wireless sensor networks: A survey. Information Fusion. 2019;49:1–25.72

Rickard
J
, 
Ahmed
S
, 
Baruch
M
, 
Klocman
B
, 
Martin
DO
, 
Menon
V
. Utility of a novel watch‐based pulse detection system to detect pulselessness in human subjects. Heart Rhythm. 2011;8:895–1899.10.1016/j.hrthm.2011.07.0302180239373

Chan
J
, 
Rea
T
, 
Gollakota
S
, 
Sunshine
JE
. Contactless cardiac arrest detection using smart devices. NPJ Digital Medicine. 2019;2:52. 10.1038/s41746-019-0128-7.31304398PMC658458274

Wu
O
, 
Briggs
A
, 
Kemp
T
, 
Gray
A
, 
MacIntyre
K
, 
Rowley
J
, 
Willett
K
. Mobile phone use for contacting emergency services in life threatening circumstances. Journal of Emergency Medicine. 2012;42:291–298.10.1016/j.jemermed.2011.02.0222214266975

Ringh
M
, 
Rosenqvist
M
, 
Hollenberg
J
, 
Jonsson
M
, 
Fredman
D
, 
Nordberg
P
, …
Svensson
L
. Mobile‐phone dispatch of laypersons for CPR in out‐of‐hospital cardiac arrest. New England Journal of Medicine. 2015;372:2316–2325.10.1056/NEJMoa14060382606183676

Bolle
SR
, 
Scholl
J
, 
Gilbert
B
. Can video mobile phones improve CPR quality when used for dispatcher assistance during simulated cardiac arrest?
Acta Anaesthesiologica Scandinavica. 2009;53:116–120.1903256910.1111/j.1399-6576.2008.01779.xPMC265937877

Choa
M
, 
Park
I
, 
Chung
HS
, 
Yoo
SK
, 
Shim
H
, 
Kim
S
. The effectiveness of cardiopulmonary resuscitation instruction: Animation versus dispatcher through a cellular phone. Resuscitation. 2008;77:87–94.1816411910.1016/j.resuscitation.2007.10.02378

Merchant
RM
, 
Abella
BS
, 
Abotsi
EJ
, 
Smith
TM
, 
Long
JA
, 
Trudeau
ME
, 
Asch
DA
. Cell phone cardiopulmonary resuscitation: Audio instructions when needed by lay rescuers: A randomized, controlled trial. Annals of Emergency Medicine. 2010;55:538–543.2020271910.1016/j.annemergmed.2010.01.02079

Abraham
WT
, 
Stevenson
LW
, 
Bourge
RC
, 
Lindenfeld
JA
, 
Bauman
JG
, 
Adamson
PB
; CHAMPION Trial Study Group
. Sustained efficacy of pulmonary artery pressure to guide adjustment of chronic heart failure therapy: Complete follow‐up results from the CHAMPION randomized trial. Lancet. 2016;387:453–461.2656024910.1016/S0140-6736(15)00723-080

Kalz
M
, 
Lenssen
N
, 
Felzen
M
, 
Rossaint
R
, 
Tabuenca
B
, 
Specht
M
, 
Skorning
M
. Smartphone apps for cardiopulmonary resuscitation training and real incident support: A mixed‐methods evaluation study. Journal of Medical Internet Research. 2014;16:e89.2464736110.2196/jmir.2951PMC397855581

Paal
P
, 
Pircher
I
, 
Baur
T
, 
Gruber
E
, 
Strasak
AM
, 
Herff
H
, …
Mitterlechner
T
. Mobile phone‐assisted basic life support augmented with a metronome. Journal of Emergency Medicine. 2012;43:472–477.10.1016/j.jemermed.2011.09.0112225760082

Paal
P
, 
Pircher
I
, 
Baur
T
, 
Gruber
E
, 
Strasak
AM
, 
Herff
H
, …
Mitterlechner
T
. Mobile phone‐assisted basic life support augmented with a metronome. Journal of Emergency Medicine. 2012;43:472–477.10.1016/j.jemermed.2011.09.0112225760083

Hatakeyama
T
, 
Nishiyama
C
, 
Shimamoto
T
, 
Kiyohara
K
, 
Kiguchi
T
, 
Chida
I
, …
Iwami
T
. A smartphone application to reduce the time to automated external defibrillator delivery after a witnessed out‐of‐hospital cardiac arrest: A randomized simulation‐based study. Simulation in Healthcare. 2018;13:387–393.2965941310.1097/SIH.0000000000000305PMC630313084

Neves Briard
J
, 
Grou‐Boileau
F
, 
El Bashtaly
A
, 
Spenard
C
, 
de Champlain
F
, 
Homier
V
. Automated external defibrillator geolocalization with a mobile application, verbal assistance or no assistance: A pilot randomized simulation (AED G‐MAP). Prehospital Emergency Care. 2019;23:420–429.3011122210.1080/10903127.2018.151101785

Sakai
T
, 
Iwami
T
, 
Kitamura
T
, 
Nishiyama
C
, 
Kawamura
T
, 
Kajino
K
, …
Shimazu
T
. Effectiveness of the new “Mobile AED Map” to find and retrieve an AED: A andomized controlled trial. Resuscitation. 2011;82:69–73.2105113010.1016/j.resuscitation.2010.09.46686

Boutilier
JJ
, 
Brooks
SC
, 
Janmohamed
A
, 
Byers
A
, 
Buick
JE
, 
Zhan
C
, …
Chan
TCY
. Optimizing a drone network to deliver automated external defibrillators. Circulation. 2017;135:2454–2465.2825483610.1161/CIRCULATIONAHA.116.026318PMC551653787

Claesson
A
, 
Bäckman
A
, 
Ringh
M
, 
Svensson
L
, 
Nordberg
P
, 
Djärv
T
, 
Hollenberg
J
. Time to delivery of an automated external defibrillator using a drone for simulated out‐of‐hospital cardiac arrests vs emergency medical services. Journal of the American Medical Association. 2017;317:2332–2334.2860952510.1001/jama.2017.3957PMC581500488

Andelius
L
, 
Hansen
MC
, 
Lippert
FK
, 
Karlsson
L
, 
Torp‐Pedersen
C
, 
Kjær Ersbøll
A
, …
Folke
F
. Smartphone activation of citizen responders to facilitate defibrillation in out‐of‐hospital cardiac arrest. Journal of the American College of Cardiology. 2020;76:43–53. 10.1016/j.jacc.2020.04.073.3261616289

Chung
MK
, 
Eckhardt
LL
, 
Chen
LY
, 
Ahmed
HM
, 
Gopinathannaie
R
, 
Joglar
JA
, …
Trulock
KM
. Lifestyle and risk factor modification for reduction of atrial fibrillation: A scientific statement from the American Heart Association. Circulation. 2020;141:e750–e772. 10.1161/CIR.0000000000000748.3214808690

Pizzetti
F
, 
Turazza
FM
, 
Franzosi
MG
, 
Barlera
S
, 
Ledda
A
, 
Maggioni
AP
, …
Tognoni
G
. Incidence and prognostic significance of atrial fibrillation in acute myocardial infarction: The GISSI‐3 data. Heart. 2001;86:527–532.1160254510.1136/heart.86.5.527PMC172996991

Gibson
CM
, 
Holmes
D
, 
Mikdad
IG
, 
Presser
D
, 
Wohns
D
, 
Yee
MK
, 
Krucoff
MW
. Implantable cardiac alert system for early recognition of ST‐segment elevation myocardial infarction. Journal of the American College of Cardiology. 2019;73:1919–1927.3084202810.1016/j.jacc.2019.01.01492

Holmes
DR
Jr
, 
Krucoff
MW
, 
Mullin
C
, 
Mikdadi
G
, 
Presser
D
, 
Wohns
D
, …
Gibson
CM
. Implanted monitor alerting to reduce treatment delay in patients with acute coronary syndrome events. Journal of the American College of Cardiology. 2019;74:2047–2055.3162376210.1016/j.jacc.2019.07.08493

Moser
DK
, 
Kimble
LP
, 
Alberts
MJ
, 
Alonzo
A
, 
Croft
JB
, 
Dracup
K
, …
Zerwic
JJ
. American Heart Association Council on Cardiovascular Nursing and Stroke Council. Reducing delay in seeking treatment by patients with acute coronary syndrome and stroke: A scientific statement from the American Heart Association Council on Cardiovascular Nursing and Stroke Council. Circulation. 2006;114:168–182.1680145810.1161/CIRCULATIONAHA.106.17604094

Van Heuverswyn
F
, 
De Buyzere
M
, 
Coeman
M
, 
de Pooter
J
, 
Drieghe
B
, 
Duyschaever
M
, …
Gheeraert
P
. Feasibility and performance of a device for automatic self‐detection of symptomatic acute coronary artery occlusion in outpatients with coronary artery disease: A multicentre observational study. Lancet Digital Health. 2019;1:e90–e99.3332323310.1016/S2589-7500(19)30026-395

Avila
CO
. Novel use of apple watch 4 to obtain 3‐lead electrocardiogram and detect cardiac ischemia. The Permanente Journal. 2019;23:19–025.10.7812/TPP/19-025PMC66364753131473496

Clemmensen
P
, 
Loumann‐Nielsen
S
, 
Sejersten
M
. Telemedicine fighting acute coronary syndromes. Journal of Electrocardiology. 2010;43:615–618.2083281510.1016/j.jelectrocard.2010.06.01297

Sanchez‐Ross
M
, 
Oghlakian
G
, 
Maher
J
, 
Patel
B
, 
Mazza
V
, 
Hom
D
, …
Klapholz
M
. The STAT‐MI (ST‐Segment analysis using wireless technology in acute myocardial Infarction) trial improves outcomes. Journal of the American College of Cardiology. 2011;4:222–227.10.1016/j.jcin.2010.11.0072134946298

Horwitz
LI
, 
Moriarty
JP
, 
Chen
C
, 
Fogerty
RL
, 
Brewster
UC
, 
Kanade
S
, …
Krumholz
HM
. Quality of discharge practices and patient understanding at an academic medical center. Journal of the American Medical Association Internal Medicine. 2013;173:1715–1722.2395885110.1001/jamainternmed.2013.9318PMC383687199

Ziaeian
B
, 
Araujo
KL
, 
Van Ness
PH
, 
Horwitz
LI
. Medication reconciliation accuracy and patient understanding of intended medication changes on hospital discharge. Journal of General Internal Medicine. 2012;27:1513–1520.2279820010.1007/s11606-012-2168-4PMC3475816100

Vollmer
WM
, 
Owen‐Smith
AA
, 
Tom
JO
, 
Laws
R
, 
Ditmer
DG
, 
Smith
DH
, …
Williams
A
. Improving adherence to cardiovascular disease medications with information technology. The American Journal of Managed Care. 2014;20(SP17):SP502–SP510.25811824PMC6358176101

Chow
CK
, 
Redfern
J
, 
Hillis
GS
, 
Thakkar
J
, 
Santo
K
, 
Hackett
ML
, …
Thiagalingam
A
. Effect of lifestyle‐focused text messaging on risk factor modification in patients with coronary heart disease: A randomized clinical trial. Journal of the American Medical Association. 2015;314:1255–1263.2639384810.1001/jama.2015.10945102

Unal
E
, 
Giakoumidakis
K
, 
Khan
E
, 
Patelarou
E
. Mobile phone text messaging for improving secondary prevention in cardiovascular diseases: A systematic review. Heart Lung. 2018;47:351–359.2980329710.1016/j.hrtlng.2018.05.009103

Shariful Islam
SM
, 
Farmer
AJ
, 
Bobrow
K
, 
Maddison
R
, 
Whittaker
R
, 
Pfaeffli Dale
LA
, …
Chow
CK
. Mobile phone text‐messaging interventions aimed to prevent cardiovascular diseases (Text2PreventCVD): Systematic review and individual patient data meta‐analysis. Open Heart. 2019;6:e001017.3167338110.1136/openhrt-2019-001017PMC6802999104

Park
LG
, 
Beatty
A
, 
Stafford
Z
, 
Whooley
MA
. Mobile phone interventions for the secondary prevention of cardiovascular disease. Progress in Cardiovascular Diseases. 2016;58:639–650.2700124510.1016/j.pcad.2016.03.002PMC4904827105

Ritchey
MD
, 
Maresh
S
, 
McNeely
J
, 
Shaffer
T
, 
Jackson
SL
, 
Keteyian
SJ
, …
Wright
J
. Tracking cardiac rehabilitation participation and completion among medicare beneficiaries to inform the efforts of a national initiative. Circulation: Cardiovascular Quality and Outcome. 2020;13:e005902.10.1161/CIRCOUTCOMES.119.005902PMC809157331931615106

Zwisler
AD
, 
Norton
RJ
, 
Dean
SG
, 
Dalal
H
, 
Tang
LH
, 
Wingham
J
, 
Taylor
RS
. Home‐based cardiac rehabilitation for people with heart failure: A systematic review and meta‐analysis. International Journal of Cardiology. 2016;221:963–969.2744147610.1016/j.ijcard.2016.06.207107

Varnfield
M
, 
Karunanithi
M
, 
Lee
CK
, 
Honeyman
E
, 
Arnold
D
, 
Ding
H
, …
Walters
DL
. Smartphone‐based home care model improved use of cardiac rehabilitation in postmyocardial infarction patients: Results from a randomised controlled trial. Heart. 2014;100:1770–1779.2497308310.1136/heartjnl-2014-305783

## 4 COMORBIDITIES

**FIGURE 6:**
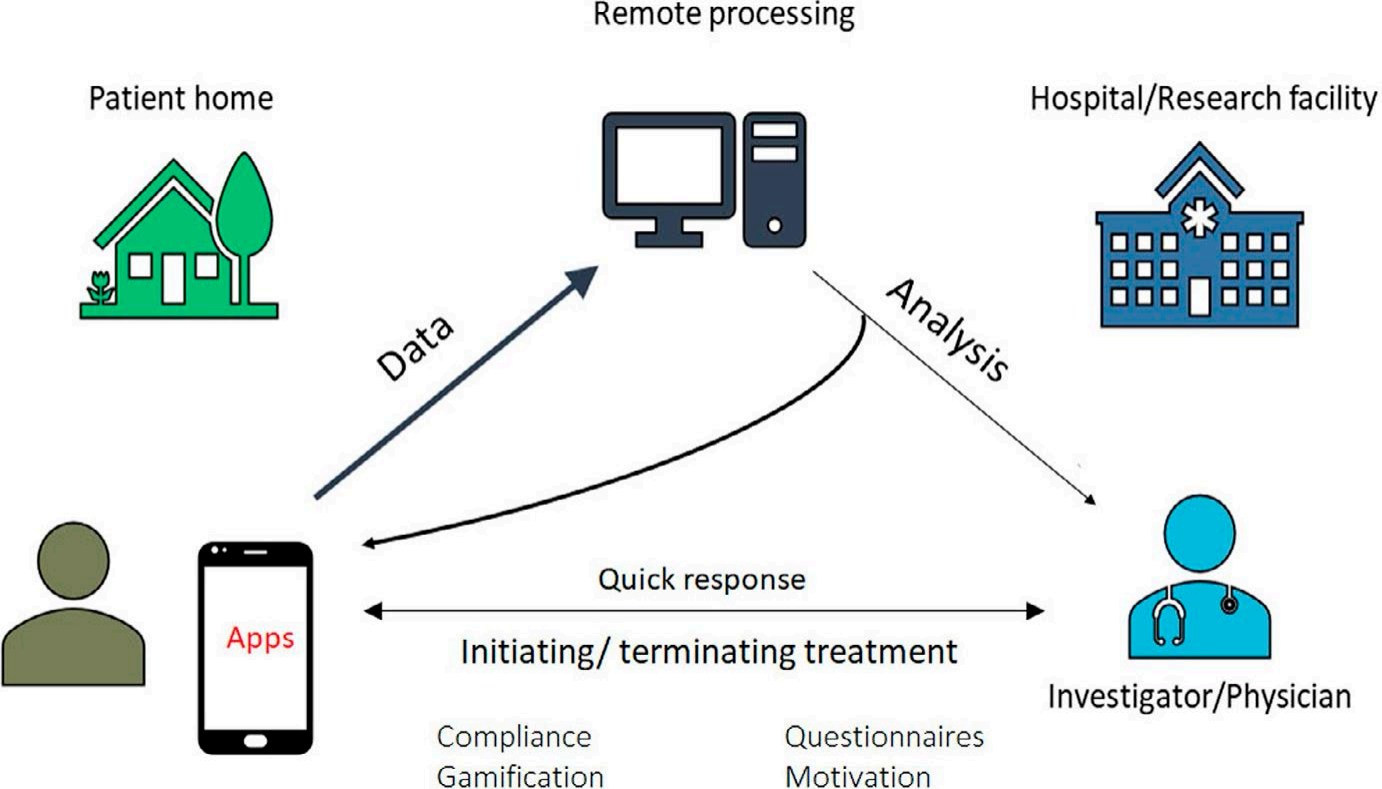
Digital applications can integrate patient relayed information of sensor and clinical information with automatic remote analysis, but also permit patients to receive advice and treatment adjustments from physicians directly.

A large proportion of arrhythmias are influenced by coexisting conditions. Their management may directly affect arrhythmia recurrence and outcome. Thus, lifestyle modifications and management of comorbid conditions (Figure 5) is becoming an objective of arrhythmia management^196^ and received a Class 1 recommendation in most recent guidelines.^130^ mHealth has significant potential for facilitating these interventions (Figure 6).

### 4.1 Ischemic heart disease

Early management (e.g., primary angioplasty) of acute ischemic syndromes may reduce infarct territory and ventricular arrhythmias, thereby improving outcome. AF after myocardial infarction worsens prognosis.^197^

#### 4.1.1 At home

ST segment monitoring technology embedded in conventionally indicated ICDs when tested in a randomized cross‐over study suggested a reduction in the time from the onset of ischemia to presentation to hospital.^91^, ^92^ The AngelMed Guardian system (Angel Medical Systems, Eatontown, New Jersey) is approved for use in the United States for patients with prior acute coronary syndrome (ACS) who remain at high risk for recurrent ACS. For lower‐risk patients, mHealth may improve symptom recognition and earlier presentation, that is, “symptom‐to‐door time”.^200^

Wearable devices that continuously monitor physiologic data promise detection, and possibly pre‐emption, of the early stages of MI, by alerting patient and/or healthcare team early. A noninvasive device consisting of a three‐lead ECG linked wirelessly to a dedicated mobile device has recently been described.^201^ Three lead ECG tracings (as well as derived augmented limb leads) can be recorded with commercially available smartwatches.^202^ Limitations of this approach are the need for the patient or a bystander to possess the device or app, and be familiar with its use, before the onset of symptoms.

An emerging technology (www.heartbeam.com) uses a credit card sized device that is pressed against the user's chest (Figure 3). It collects ECG signals using a novel 3D vector approach. The signals are sent to the cloud, where they are analyzed and compared to the patient’s asymptomatic baseline reading. A proprietary algorithm combines the signal analysis with the patient’s history and reported symptoms. This information, along with a diagnostic recommendation and ECG waveforms, is sent to the patient’s physician, who makes a final determination and informs the patient. This system is used by patients in the telehealth setting to assess whether chest pain is the result of an myocardial infarction.

#### 4.1.2 Emergency teams

The next step of patient care involved transmission of ECGs by emergency responders in the field to hospitals for review and triage and was shown to result in shorter door‐to‐balloon time, lower peak troponin and creatine phosphokinase levels, higher postinfarction left ventricular ejection fraction, and shorter length of stay compared with control patients whose ECGs were not transmitted.^96^, ^97^ This paradigm has now been widely implemented. Technical factors, such as transmission failure and lack of network coverage, are the main impediments to adoption of such systems.

#### 4.1.3 Posthospital care

This is often confusing for patients, who often exhibit a poor understanding of their medications, follow‐up procedures, and future appointments.^98^, ^99^ This contributes to frequent hospital readmissions. Mobile technologies may enable individualized contact between patients and healthcare providers. Phone calls led to a modest improvement in medication adherence in patients with coronary artery disease in one large randomized controlled trial.^207^ Text messaging was shown to increase medication adherence and improved cardiovascular risk factors.^101^, ^102^ Available evidence is limited by short‐term follow‐up and self‐reported adherence.^210^ Success may depend on personalized messages with tailored advice, the ability to respond to texts, timing messages to coincide with medication doses, higher frequency of messages, and the use of additional apps or websites.^211^ Interoperability with the EMR may facilitate this approach.

#### 4.1.4 Cardiac rehabilitation

This was shown to improve health outcomes among patients with heart disease, but is underutilized. The Million Hearts Cardiac Rehabilitation Collaborative aims to increase participation rates to ≥70% by 2022.^212^ Mobile apps and linked sensors to measure heart rate, respiration rate, and exercise parameters may overcome traditional limitations of availability, cost, and convenience and be more acceptable to some patients.^213^ A randomized controlled trial center‐based and mobile rehabilitation found improved uptake, adherence, and completion with home‐based cardiac rehabilitation in postinfaction patients.^214^ (See also 4.2.2.)

### 4.2 Heart failure

Heart failure is widely prevalent, costly to manage, and degrades patient outcomes.^108^, ^109^ HF may trigger AF and ventricular arrhythmias. Conversely, AF may precipitate HF. Remote monitoring of, for example, dietary and medication adherence (See Section 4.6.2), detection of arrhythmias (See Section 3), intercurrent ischemia (See Section 4.1), orthopnea, changes in heart rate, activity, and sleep (See Section 4.5) may enable remote adjustment of management to reduce emergency department visits and unplanned HF‐related hospitalizations. If scalable, remote monitoring coupled with mobile communication could prove to reduce costs associated with HF.

Despite promise, most large, multicenter randomized trials failed to demonstrate improved outcomes of remote monitoring in HF patients (Table 4).^110^, ^111^, ^112^, ^113^, ^1^, ^2^ Combination algorithms based on multiple parameters may be valuable.^223^ One trial stands out. The TIM‐HF2 trial randomized HF patients to either remote patient management plus usual care or to usual care only and were followed up for over a year.^224^ The results showed reduction in the combined endpoint of percentage of days lost due to unplanned hospitalization and all‐cause mortality. However cardiovascular mortality was similar between remote monitoring and standard care groups. Implanted devices that monitor pulmonary arterial pressure may be beneficial in select patients when used in structured programs.^224^ The positive findings of the CHAMPION trial (CardioMEMS Heart Sensor Allows Monitoring of Pressure to Improve Outcomes in NYHA Functional Class III Heart Failure Patients) trial and subsequent FDA approval has renewed interest in remote patient management for HF patients.^5^, ^6^, ^7^ This requires daily download of hemodynamic data and a prespecified medical treatment plan. An app is also available which illustrates patient compliance with monitoring, alerts the patient when transmissions are not received, shows medication reminders, and allows for medication reconciliation and titration.

**TABLE 4:** Randomized trials with neutral results based on external‐device remote patient monitoring (RPM)

Study name	Sample size	Study design and tested modality	Potential explanantion for lack of benefit
TIM‐HF (Koehler Circulation)^219^	N = 710 (355 on RPM)	Randomized trial of a Bluetooth‐enabled device designed to follow 3‐lead electrocardiography, BP, and weight	Participants had stable HF, so it may be that remote monitoring is not as effective in lower‐risk patients
Tele‐HF (Chaudhry N Engl J Med)^218^	N= 1653 (826 on RPM)	Telephone‐based interactive voice response system with a higher risk population than in the TIM‐HF study	Patient adherence was poor, with <55% of the study subjects using the device 3 days per week by the end of the study. Interestingly, a smaller previous trial had shown benefit; this difference in results implies that how a technology is implemented might determine benefit
BEAT‐HF (Ong JAMA Intern Med)^220^	N = 1437 (715 on RPM)	Health‐coaching telephone calls with monitoring of weight, BP, HR, and symptoms in a high‐risk population with 50% rehospitalization rate	Nonadherence was the primary limitation, with only 61% of patients more than half‐adherent in the first 30 days
Mayo Clinic Study (Takahashi Arch Intern Med)^221^	N = 205 (102 on RPM)	Telemonitoring in a PC panel (various health conditions and not only HF) in the top 10% of Elder Risk Assessment Index managed with biometrics (BP, HR, weight, pulse oximetry, etc) plus daily symptom assessment. Video conference capability was present.	Abnormal telehealth data were directed to PC providers. It is unclear what action this drove. It might have caused the PC provider to direct the patient to an emergency department or a hospital. Could increased symptom surveillance actually increase healthcare utilization?
TEHAF (Boyne Eur J Heart Fail)^217^	N = 382 (197 on RPM)	Electronic device to assess symptoms and educate patients with HF. Abnormal symptoms directed to a monitoring nurse. Device tailored itself to patient’s knowledge.	Excellent adherence with use of the device. Planned and unplanned face‐to‐face HF nurse visits were higher in the control group. Event rates for both groups were lower than expected. Primary limitation appeared to be the excellent outcomes in the control group.
LINK‐HF (Stehlik, CIrc HF)^44^	N=100	Disposable multisensor chest patch for 3 months linked via smartphone to cloud analytics. Apply machine‐learning algorithm.	Pilot study, compliance eroded. However, this detected precursors of hospitalization for HF exacerbation with 76% to 88% sensitivity and 85% specificity.

Abbreviations: BP, blood pressure; HF, heart failure; HR, heart rate; PC, primary care; RPM, remote patient monitoring.

REFERENCES TABLE 4108

Albert
C
, 
Estep
JD
. Economic impact of chronic heart failure management in today’s cost‐conscious environment. Cardiac Electrophysiology Clinics. 2019;11:1–9.3071784110.1016/j.ccep.2018.11.002109

Benjamin
EJ
, 
Blaha
MJ
, 
Chiuve
SE
, 
Cushman
M
, 
Das
SR
, 
Deo
R
, …
Muntner
P
. Heart disease and stroke statistics‐2017 update: A report from the American Heart Association. Circulation. 2017;135:e146–e603.2812288510.1161/CIR.0000000000000485PMC5408160110

Boyne
JJ
, 
Vrijhoef
HJ
, 
Crijns
HJ
, 
De Weerd
G
, 
Kragten
J
, &
Gorgels
AP
; TEHAF investigators
. Tailored telemonitoring in patients with heart failure: Results of a multicentre randomized controlled trial. European Journal of Heart Failure. 2012;14:791–801.2258831910.1093/eurjhf/hfs058111

Chaudhry
SI
, 
Mattera
JA
, 
Curtis
JP
, 
Spertus
JA
, 
Herrin
J
, 
Lin
Z
, …
Krumholz
HM
. Telemonitoring in patients with heart failure. New England Journal of Medicine. 2010;363:2301–2309.10.1056/NEJMoa1010029PMC323739421080835112

Koehler
F
, 
Winkler
S
, 
Schieber
M
, 
Sechtem
U
, 
Stangl
K
, 
Böhm
M
, …
Anker
SD
. Telemedical Interventional Monitoring in Heart Failure Investigators. Impact of remote telemedical management on mortality and hospitalizations in ambulatory patients with chronic heart failure: The Telemedical Interventional Monitoring in Heart Failure study. Circulation. 2011;123:1873–80.2144488310.1161/CIRCULATIONAHA.111.018473113

Ong
MK
, 
Romano
PS
, 
Edgington
S
, 
Aronow
HU
, 
Auerbach
AD
, 
Black
JT
, …
Fonarow
GC
. Better effectiveness after transition‐heart failure (BEAT‐HF) research group. Effectiveness of remote patient monitoring after discharge of hospitalized patients with heart failure: The better effectiveness after transition—Heart failure (BEAT‐HF) randomized clinical trial. Journal of the American Medical Association Internal Medicine. 2016;176:310–318.2685738310.1001/jamainternmed.2015.7712PMC4827701

#### 4.2.1 Mobile technologies for managing heart failure

The concept of coupling remote monitoring and mobile cellular technologies is attractive for the HF community^8^, ^9^ Heart rate (ECG), BP, and weight were the most frequently monitored parameters. Sensors that detect respiratory rate and pattern by detecting movement of the chest wall, via pressure, stretch, or accelerometry, may have applications in HF. Detecting breathing via microphone (sounds), change in impedance, or pulse oximetry are other possible means to monitor respiratory function. Some of these modalities could be integrated into smart clothing.^230^

Some trials included also alert reminders of medication use, voice messages on educational tips, video education, and tracking of physical activity (See Section 4.6.1). Patients were mostly monitored daily and followed for an average of 6 months. A reduction was seen in HF‐related hospital days.^228^ High rates of patient engagement, acceptance, usage and adherence have been reported in some trials but not others.^11^, ^12^

Preliminary results using a disposable multisensor chest patch in the LINK‐HF study were encouraging,^44^ detecting precursors of hospitalization for HF exacerbation with 76% to 88% sensitivity and 85% specificity, 1 week before clinical manifestations.

#### 4.2.2 Hybrid telerehabilitation in patients with heart failure

Exercise training is recommended for all stable HF patients.^13^, ^14^ Hybrid cardiac telerehabilitation is a novel approach. Telerehabilitation is the supervision and performance of comprehensive cardiac rehabilitation at a distance, encompassing: telemonitoring (minimally intrusive, often involving sensors), teleassessment (active remote assessment), telesupport (supportive televisits by nurses, psychological support), teletherapy (actual interactive therapy), telecoaching (support and instruction for therapy), and teleconsulting and telesupervision of exercise training.^235^ Various devices have been described, from heart rate monitoring^236^ and transtelephonic electrocardiographic monitoring^237^ to tele‐ECG‐monitoring via a remote device^238^ and real‐time ECG and voice transtelephonic monitoring.^239^

Home‐based telerehabilitation was demonstrated to be safe, effective with high adherence among HF patients. It improves physical capacity^240^ and psychological status,^241^ with similar QoL improvement to standard rehabilitation.^240^ The first randomized, prospective, multicenter study (TELEREH‐HF) showed that hybrid telerehabilitation and telecare in HF patients was more effective than usual care in improving peak VO2, 6‐minute walk distance, and QoL, although not associated with reduction of 24‐month mortality and hospitalization except in the most experienced centers.^22^, ^23^

The recent Scientific Statement from the American Association of Cardiovascular and Pulmonary Rehabilitation, the AHA, and the ACC indicates that home‐based rehabilitation using telemedicine is a promising new direction.^244^

### 4.3 Diabetes

Diabetes mellitus is a strong risk factor for the development of morbidity and mortality associated with a range of cardiovascular diseases. Metabolic syndrome (elevated blood glucose and insulin resistance) acts via multiple mechanisms resultant in microand macrovascular complications, development of autonomic neuropathy, diastolic dysfunction, renal failure, and AF. Important management goals are lifestyle changes (e.g., diet and activity: see later section) to prevent disease development and tight glycemic control, especially for type 1 diabetes mellitus which demands lifelong rigorous self‐monitoring.^25^, ^26^, ^27^, ^28^, ^29^, ^30^ mHealth modalities self‐management was recommended recently by ESC guidelines on diabetes and cardiovascular diseases to.^251^

Glycemic control may reduce AF development and recurrence.^32^, ^33^, ^34^, ^35^

Mobile apps can facilitate self‐management by reminding regular assessment of required parameters and medications to take and provide educational tools and motivational support. Regular transmission of blood glucose levels from patients to their physicians can be based on SMS, email, or diverse web‐based services. Bluetooth‐enabled glucose meters are frequently used.^36^, ^37^ BlueStar (Welldoc, Columbia, MD), first to receive US FDA clearance for diabetes mellitus management, comes with an app which requires a physician prescription and enables patients to titrate insulin dosing by using the proprietary insulin calculator. The Freestyle LibreLink app (Abbott Laboratories, Abbott Park, IL) reads an associated continuous glucose monitoring device and displays trends.^258^

Stand‐alone diabetes management apps have recently been reviewed.^259^ Short‐term measures, such as HbA1c, may be improved by such apps in conjunction with clinical support, but many have suboptimal usability.^260^ Phone‐based interventions were associated with improved glycemic control as compared to standard care.^41^, ^42^, ^43^, ^44^ Efficacy for improving glycemic control in randomized controlled trials has shown mixed results.^45^, ^46^ Meta‐analyses indicate that mobile phone interventions for self‐management reduced HbA1c modestly by 0.2‐0.5% over a median of 6‐month follow‐up duration, with a greater reduction in patients with type 2 compared to type 1 diabetes.^267^ A significant impact on clinical outcomes may affect healthcare expenditures by reducing the need for in‐person contact with healthcare providers, preventing hospital admissions, and improving prognosis. In a retrospective study, the use of mHealth technologies was associated with a 21.9% reduction in medical spending than a control group during the first year.^268^ Key determinants to successful uptake of decision‐support apps will be their user‐friendliness and complexity and the delivery of electronic communications and feedback to the patient.

### 4.4 Hypertension

Hypertension, because of its high prevalence, provides the highest attributable risk for the development of AF.^269^

mHealth strategies for hypertension comprise a continuum of solutions, used by consumers or healthcare providers, and includes wireless diagnostic and clinical decision‐support tools, aiming to monitor health status and improve health outcomes. BP telemonitoring is one of the most commonly used strategies and includes remote data transmission of BP and clinical information from patients in their home or from a community setting to a central service, where they are reviewed by a managing physician for treatment adjustments. Several clinical trials have shown that BP telemonitoring might be more effective than usual care in achieving target BP.^50^, ^51^, ^52^ A meta‐analysis showed that, compared with usual care, BP telemonitoring improved office systolic BP and diastolic BP by 3.99 mmHg (95% confidence interval (CI): 5.06–2.93; P < 0.001) and 1.99 mmHg (95% CI: −2.60 to −1.39; P < 0.001), respectively.^273^ BP telemonitoring nested in a more complex intervention, including additional support, as face‐to‐face counseling, telecounseling, education, behavioral management, medication management, and adherence contracts, led to additional and more sustainable benefit.^53^, ^54^

mHealth has the potential to promote patient self‐management, as a complement to the doctor's intervention, and encourage greater participation in medical decision‐making. Indeed, the TASMINH4 unblinded randomized controlled trial showed that patients who used self‐monitoring of BP to titrate antihypertensives, with or without telemonitoring, achieved better BP control than those assigned to usual care.^275^ The self‐monitoring group that used telemonitoring achieved lower BP quicker than the self‐monitoring group not receiving telemonitoring support, but readings were not significantly different at 1 year of follow‐up. Cost‐effectiveness analysis suggests that self‐monitoring in this context is cost‐effective by NICE criteria, that is, costing well under £20,000 per QALY.^276^

Although mHealth options may aid hypertension management, technological barriers, high costs, heterogeneity of solutions and technologies, and lack of standards challenge clinical implementation. The 2019 ESC guidelines on hypertension stress the importance of self‐monitoring and underline the potential use of smartphone‐based solutions. Nevertheless, they do not recommend the use of mobile apps as independent mean of BP measurements (Williams ESC/ESH guidelines).^277^

### 4.5 Disorders Including Sleep Apnea (See also Heart Failure Section 4.2.1)

Sleep disorders are widely prevalent and contribute to cardiovascular risk and arrhythmias, especially AF.^58^, ^59^, ^60^, ^61^, ^62^ This maybe because sleep disturbance is intimately tied to circadian rhythms and sympatho‐vagal balances.^284^ Standard sleep disorder diagnostics have been validated but require technical support for data acquisition and scoring. For example, polysomnography has long been considered the gold‐standard for acquisition of rich multimodal cardio‐neurorespiratory objective physiologic data to ascertain sleep architecture, total sleep time, and cardiorespiratory abnormalities and is primarily used for the diagnosis of obstructive sleep apnea. Actigraphy has the advantage of collecting objective data over days and nights to characterize sleep–wake patterning and provide measures of total sleep time, sleep efficiency, and sleep onset latency in addition to surrogate circadian measures. However, such tests are obtrusive and expensive.

- Treating sleep apnea may reduce AF burden^65^, ^66^

Consumer technology directed to sleep medicine may revolutionize the detection and treatment of sleep disorders. Since such apps are preinstalled on many smartphones, sleep tracking may be among the most widely applied facets of mHealth.^287^ Applications include mobile device applications, wearable devices, embedded devices (in the individual’s sleep environment), rings (https://bodimetrics.com/product/circul‐sleep‐and‐fitnessring), integration of accessory diagnostic monitoring (e.g., oximetry, ECG monitoring), and sleep therapy adherence monitoring. Several commercially available wearable devices measure total sleep time accurately, but not more detailed parameters such as sleep efficiency and different sleep stages.^288^ Preliminary data suggest that wearable devices may be capable of detecting sleep apnea with good accuracy compared to gold‐standard polysomnography^289^ and transform the approach to sleep disorder screening, diagnosis, and treatment. Sleep irregularity diagnosed by 7 day wrist actigraphy was linked to risk of cardiovascular events.^290^ Preliminary studies indicated that use of wearables may permit behavior modifications that improve sleep quality.^291^ In this regard, mHealth applications to sleep diagnosis and treatment promise facilitation of rhythm control.

### 4.6 Lifestyle

#### 4.6.1 Physical activity

Physical activity is any bodily movement from skeletal muscle contraction to increase energy expenditure above basal level (see Figure 5). Athletic activity varies from recreational sports to competitive events. There is a compelling evidence that regular aerobic exercise at the levels recommended by Physical Activity Guidelines Advisory Committee reduces the risk of a variety of cardiovascular conditions, including AF.^72^, ^73^, ^74^ However, the majority of the population is not engaged in physical activity at the recommended levels.^294^ Among patients with cardiovascular disease, patient activity measured automatically by ICDs correlated with survival following ICD implantation ^295^ Fitness represents an enormous market for mobile technologies and significant opportunity to improve the health of a wide range of mHealth consumers. In 2017, over 318,000 “fitness and health” apps were available, almost double the number two years prior.^296^ Many of these recreational apps monitor daily physical activity and support a healthy lifestyle by counting the number of steps daily, online training, and motivation coaching.^297^

- Cardiorespiratory fitness has an inverse relationship to AF burden.^298^
- Improvement in exercise capacity of 2 METs in overweight individuals may double freedom from AF.^299^

Consumer‐grade fitness technology includes individual fitness trackers that can stand alone, a fitness tracker that is coupled with a companion app, or an app that can be downloaded onto a smartphone, which then utilizes various features of the smartphone to measure activity and sleep. The accuracy of these measurements varies between different products and between measures within the same product.^300^ Furthermore, while step‐counting is long established, measuring the intensity of exercise is more complex. Although fitness technology has the exciting potential to increase physical activity by promoting goal setting and providing feedback, its effectiveness in motivating positive behavioral change remains unclear.^301^

One cautionary tale is the study by Jakicic et al. that examined the effectiveness of a lifestyle intervention with or without a fitness tracker.^302^ Two groups received instruction to promote physical activity and dietary restriction. Six months into the intervention, half of the participants were provided with an upper arm fitness tracker and web‐based support accompanying the device. The other half logged and tracked their activity and diet on a study website. Of note, the group that wore the tracker lost less weight than the group who did not. Moreover, changes in physical activity between the two groups were not significantly different. These results cast doubt on the effectiveness of fitness trackers in promoting greater physical activity, and thus, further data are required to assess the impact of this approach (see Section 5).

##### Competitive athletes

These are a unique category. Endurance athletes may have increased AF risk.^83^, ^84^ Remote evaluation of ECG recordings may be useful in countries that perform preparticipation ECG screening.^85^, ^86^ Mobile devices and apps provide complex data which can be used as a self‐monitoring tool for managing training.^87^, ^88^, ^89^, ^90^, ^91^ Exercise load and performance level can be accessed on a regular basis by coaches as well as athletes. Training guided by daily monitoring of HRV parameters has also been proposed, but data are limited.^92^, ^93^, ^94^ Mobile devices provide the possibility of online real‐time monitoring during indoor and outdoor training and competitions. Monitoring of heart rate provides both information on performance and level of training but can also provide valuable information regarding heart rhythm irregularity suggestive of arrhythmias. Any kind of paroxysmal arrhythmia related to sport participation and detected by mobile devices designed merely for heart rate assessment should trigger further cardiological evaluation. Having in mind data indicating that sports participation may be associated with higher risk of development of AF mobile devices may serve as valuable screening tool for AF detection.

Importantly, mHealth solutions enable easy access to athletes’ medical data. The latter approach can be of special interest in management of athletes’ health during competitions abroad.

#### 4.6.2 Diet

In 2010, the American Heart Assiociation promulgated “Life’s Simple 7” as a public health strategy to improve cardiovascular health with the motto: “7 Small Steps to Big Changes. It’s easy and simple. Anyone can do it. Start with one or two!” Unfortunately, research has shown that this strategy is anything but simple: virtually, no adults (<1%) are compliant with all recommendations and 42% are compliant with only 0‐2 recommendations.^315^ Although there is ample evidence that weight loss and maintaining an ideal weight are beneficial in reducing AF burden and symptoms, compliance with this recommendation is poor; the reasons include among others, inability to track food intake.^96^, ^97^, ^98^

- Weight loss combined with risk factor modification is a Class 1 (B‐R) recommendation in treatment of AF^130^
- >10% weight reduction/target BMI <27 kg/m^2^ reduces AF burden.^318^

There are currently many consumer‐oriented mobile phone‐based applications (apps) designed for tracking food intake, but their utility for use in carbohydrate counting is limited due their design.^319^ Commonly, these consumer‐oriented apps require multiple steps. As an example, the user types in the food consumed and then scrolls through the search results to match with the program’s food and nutrient database. Next, after finding a matching food type, the user must estimate and enter an amount. These apps require significant user input and time burden along with high possibility of error. In addition, they are also plagued by uncertain accuracy. Recently, research has shown that nutrient calculations from leading nutrition tracking apps tended to be lower than results from using 24‐hour recall with analysis by the Nutrition Data System for Research (NDSR), a research‐level dietary analysis software.^320^

By contrast, a visual image‐based app, such as the Technology‐Assisted Dietary Assessment (TADA) system, directly addresses the aforementioned shortcomings.^101^, ^102^, ^103^ This is in research phase. The TADA system consists of two main components: (a) A smartphone app that runs on either iPhones (iOS) or Android devices: the Mobile Food Record (mFR), and (b) Cloud‐based server that communicates with the mFR, processes, and stores the food images. Using the TADA system, a person takes a photograph of the meal they are planning to eat using their smartphone’s camera. The use of geometric models has permitted the TADA system to use a single image of a meal to estimate portion size to within 15% of the actual amount.^324^ Hence, smartphone‐based technology such as the TADA system can facilitate tracking of food intake, which in turn can potentially help with weight management.

Despite the profusion of diet‐ and weight‐related apps, and the interest in weight loss in the community, there remains a dearth of high‐quality evidence that these apps are actually effective.^325^ There remains a need for further evidence development before specific apps or other mHealth technology can be recommended or prescribed.

REFERENCES SECTION 41

Takahashi
PY
, 
Pecina
JL
, 
Upatising
B
, 
Chaudhry
R
, 
Shah
ND
, 
Van Houten
H
, …
Hanson
GJ
. A randomized controlled trial of telemonitoring in older adults with multiple health issues to prevent hospitalizations and emergency department visits. Archives of Internal Medicine. 2012;172:773–779.2250769610.1001/archinternmed.2012.256PMC39142002

Dickinson
M
, 
Allen
L
, 
Albert
NA
, 
DiSalvo
T
, 
Ewald
GA
, 
Vest
AR
, …
Givertz
MM
. Consensus Statement. Remote monitoring of patients with heart failure: A white paper from the Heart Failure Society of America Scientific Statements Committee. Journal of Cardiac Failure. 2018;24:682–694. 10.1016/j.cardfail.2018.08.011.303082423

Ono
M
, 
Varma
N
. Remote monitoring to Improve long‐term prognosis in heart failure patients with implantable cardioverter‐defibrillators. Expert Review of Medical Devices. 2017;14:335–342.2829995610.1080/17434440.2017.13064384

Koehler
F
, 
Koehler
K
, 
Deckwart
O
, 
Prescher
S
, 
Wegscheider
K
, 
Kirwan
BA
, …
Stangl
K
. Efficacy of telemedical interventional management in patients with heart failure (TIM‐HF2): A randomised, controlled, parallel‐group, unmasked trial. Lancet. 2018;392:1047–1057. 10.1016/S0140-6736(18)31880-4.301539855

Abraham
WT
, 
Stevenson
LW
, 
Bourge
RC
, 
Lindenfeld
JA
, 
Bauman
JG
, 
Adamson
PB
; CHAMPION Trial Study Group
. Sustained efficacy of pulmonary artery pressure to guide adjustment of chronic heart failure therapy: Complete follow‐up results from the CHAMPION randomized trial. Lancet. 2016;387:453–461.2656024910.1016/S0140-6736(15)00723-06

Carbo
A
, 
Gupta
M
, 
Tamariz
L
, 
Palacio
A
, 
Levis
S
, 
Nemeth
Z
, 
Dang
S
. Mobile technologies for managing heart failure: A systematic review and meta‐analysis. Telemedicine and e‐Health. 2018;24(12):958–968. 10.1089/tmj.2017.0269.296084307

Desai
AS
, 
Bhimaraj
A
, 
Bharmi
R
, 
Jermyn
R
, 
Bhatt
K
, 
Shavelle
D
, …
Heywood
JT
. Ambulatory hemodynamic monitoring reduces heart failure hospitalizations in “real‐world” clinical practice. Journal of the American College of Cardiology. 2017;69:2357–65.2833075110.1016/j.jacc.2017.03.0098

Carbo
A
, 
Gupta
M
, 
Tamariz
L
, 
Palacio
A
, 
Levis
S
, 
Nemeth
Z
, 
Dang
S
. Mobile technologies for managing heart failure: A systematic review and meta‐analysis. Telemedicine and e‐Health. 2018;24:958–968. 10.1089/tmj.2017.0269.296084309

Cipresso
P
, 
Serino
S
, 
Villan
ID
, 
Repetto
C
, 
Sellitti
L
, 
Albani
A
, …
Rivaet
G
. Is your phone so smart to affect your states? An exploratory study based on psychophysiological measures. Neurocomputing. 2012;84:23–30.10

Molinaro
N
, 
Massaroni
C
, 
Lo Presti
D
, 
Saccomandi
P
, 
Di Tomaso
G
, 
Zollo
L
, …
Schena
E
. Wearable textile based on silver plated knitted sensor for respiratory rate monitoring. *Conference Proceedings IEEE Engineering in Medicine and Biology Society (EMBC)*, 2018;2865–2868. 10.1109/EMBC.2018.8512958.3044099911

Chaudhry
SI
, 
Mattera
JA
, 
Curtis
JP
, 
Spertus
JA
, 
Herrin
J
, 
Lin
Z
, …
Krumholz
HM
. Telemonitoring in patients with heart failure. New England Journal of Medicine. 2010;363:2301–2309.10.1056/NEJMoa1010029PMC32373942108083512

Hamilton
SJ
, 
Mills
B
, 
Birch
EM
, 
Thompson
SC
. Smartphones in the secondary prevention of cardiovascular disease: A systematic review. BMC Cardiovascular Disorders. 2018;18:25.2941568010.1186/s12872-018-0764-xPMC580399813

Piepoli
MF
, 
Conraads
V
, 
Corrà
U
, 
Agoston
IP
, 
Coats
AJ
, 
Conraads
V
, …
Ponikowski
PP
. Exercise training in heart failure: from theory to practice. A consensus document of the Heart Failure Association and the European Association for Cardiovascular Prevention and Rehabilitation. European Journal of Heart Failure. 2011;13:347–357.2143636010.1093/eurjhf/hfr01714

Ponikowski
P
, 
Voors
AA
, 
Anker
SD
, 
Bueno
H
, 
Cleland
JGF
, 
Coats
AJS
, …
van der Meer
P
. ESC Scientific Document Group. 2016 ESC Guidelines for the diagnosis and treatment of acute and chronic heart failure: The Task Force for the diagnosis and treatment of acute and chronic heart failure of the European Society of Cardiology (ESC) Developed with the special contribution of the Heart Failure Association (HFA) of the ESC. European Heart Journal. 2016;37:2129–2200.2720681910.1093/eurheartj/ehw12815

Piotrowicz
E
, 
Piepoli
MF
, 
Jaarsma
T
, 
Lambrinou
E
, 
Coats
AJ
, 
Schmid
JP
, …
Ponikowski
PP
. Telerehabilitation in heart failure patients: The evidence and the pitfalls. International Journal of Cardiology. 2016;220:408–413.2739096310.1016/j.ijcard.2016.06.27716

Smart
N
, 
Haluska
B
, 
Jeffriess
L
, 
Marwick
TH
. Predictors of a sustained response to exercise training in patients with chronic heart failure: A telemonitoring study. American Heart Journal. 2005;150:1240–1247.1633826610.1016/j.ahj.2005.01.03517

Kouidi
E
, 
Farmakiotis
A
, 
Kouidis
N
, 
Deligiannis
A
. Transtelephonic electrocardiographic monitoring of an outpatient cardiac rehabilitation programme. Clinical Rehabilitation. 2006;20:1100–1104.1714852210.1177/026921550607125618

Piotrowicz
E
, 
Stepnowska
M
, 
Leszczyńska‐Iwanicka
K
, 
Piotrowska
D
, 
Kowalska
M
, 
Tylka
J
, …
Piotrowicz
R
. Quality of life in heart failure patients undergoing home‐based telerehabilitation versus outpatient rehabilitation – A randomized controlled study. European Journal of Cardiovascular Nursing. 2015;14:256–263.2484930410.1177/147451511453702319

Ades
PA
, 
Pashkow
FJ
, 
Fletcher
G
, 
Pina
IL
, 
Zohman
LR
, 
Nestor
JR
. A controlled trial of cardiac rehabilitation in the home setting using electrocardiographic and voice transtelephonic monitoring. American Heart Journal. 2000;139:543–548.1068927110.1016/s0002-8703(00)90100-520

Piotrowicz
E
, 
Zieliński
T
, 
Bodalski
R
, 
Rywik
T
, 
Dobraszkiewicz‐Wasilewska
B
, 
Sobieszczańska‐Małek
M
, …
Piotrowicz
R
. Home‐based telemonitored Nordic walking training is well accepted, safe, effective and has high adherence among heart failure patients, including those with cardiovascular implantable electronic devices – A randomized controlled study. European Journal of Preventive Cardiology. 2015;22:1368–1377.2526126810.1177/204748731455153721

Piotrowicz
E
, 
Piotrowski
W
, 
Piotrowicz
R
. Positive effects of the reversion of depression on the sympathovagal balance after telerehabilitation in heart failure patients. Annals of Noninvasive Electrocardiology. 2016;21:358–368.2652469910.1111/anec.12320PMC693159622

Piotrowicz
E
, 
Pencina
MJ
, 
Opolski
G
, 
Zareba
W
, 
Banach
M
, 
Kowalik
I
, …
Piotrowicz
R
. Effects of a 9‐week hybrid comprehensive telerehabilitation program on long‐term outcomes in patients with heart failure. The telerehabilitation in heart failure patients (TELEREH‐HF) randomized clinical trial. Journal of the American Medical Association Cardiology. 2019;5(3):300–308. 10.1001/jamacardio.2019.5006.PMC68653253173470123

Piotrowicz
E
, 
Piotrowicz
R
, 
Opolski
G
, 
Pencina
M
, 
Banach
M
, 
Zaręba
W
. Hybrid comprehensive telerehabilitation in heart failure patients (TELEREH‐HF): A randomized, multicenter, prospective, open‐label, parallel group controlled trial‐Study design and description of the inter vention. American Heart Journal. 2019;217:148–158.3165494410.1016/j.ahj.2019.08.01524

Thomas
RJ
, 
Beatty
AL
, 
Beckie
TM
, 
Brewer
LC
, 
Brown
TM
, 
Forman
DE
, …
Whooley
MA
. Home‐based cardiac rehabilitation. A scientific statement from the American Association of Cardiovascular and Pulmonary Rehabilitation the American Heart Association, and the American College of Cardiology. Journal of the American College of Cardiology. 2019;74:133–153.3109725810.1016/j.jacc.2019.03.008PMC734111225

Balakumar
P
, 
Maung‐U
K
, 
Jagadeesh
G
. Prevalence and prevention of cardiovascular disease and diabetes mellitus. Pharmacological Research. 2016;113(Pt A):600–609.2769764710.1016/j.phrs.2016.09.04026

Donnelan
E
, 
Aagaard
P
, 
Kanj
M
, 
Jabe
RW
, 
Elshazly
M
, 
Hoosien
M
, …
Wazni
O
. Association between pre‐ablation glycemic control and outcomes among patients with diabetes undergoing atrial fibrillation ablation. Journal of the American Medical Association Clinical Electrophysiology. 2019;5:397–903.10.1016/j.jacep.2019.05.0183143928927

Goudis
CA
, 
Korantzopoulos
P
, 
Ntalas
IV
, 
Kallergis
EM
, 
Liu
T
, 
Ketikoglou
DG
. Diabetes mellitus and atrial fibrillation: Pathophysiological mechanisms and potential upstream therapies. International Journal of Cardiology. 2015;184:617–622.2577084110.1016/j.ijcard.2015.03.05228

Wang
A
, 
Green
JB
, 
Halperin
JL
, 
Piccini
JP
Sr
. Atrial fibrillation and diabetes mellitus. Journal of the American College of Cardiology. 2019;74:1107–1115.3143922010.1016/j.jacc.2019.07.02029

Wilkinson
MJ
, 
Zadourian
A
, 
Taub
PR
. Heart failure and diabetes mellitus: Defining the problem and exploring the interrelationship. American Journal of Cardiology. 2019;124(Suppl 1):S3–S11.10.1016/j.amjcard.2019.10.0243174143830

Wingerter
R
, 
Steiger
N
, 
Burrows
A
, 
Estes
NAM
. Impact of lifestyle modification on atrial fibrillation. American Journal of Cardiology. 2020;125:289–297. 10.1016/j.amjcard.2019.10.018.3176114731

Cosentino
F
, 
Grant
PJ
, 
Aboyans
V
, 
Bailey
CJ
, 
Ceriello
A
, …
Wheeler
DC
. ESC Scientific Document Group. 2019 ESC Guidelines on diabetes, pre‐diabetes, and cardiovascular diseases developed in collaboration with the EASD. European Heart Journal. 2020;41:255–323. 10.1093/eurheartj/ehz486.3149785432

Chang
SH
, 
Wu
LS
, 
Chiou
MJ
, 
Liu
JR
, 
Yu
KH
, 
Kuo
CF
, …
See
LC
. Association of metformin with lower atrial fibrillation risk among patients with type 2 diabetes mellitus: A population‐based dynamic cohort and in vitro studies. Cardiovascular Diabetology. 2014;13:123. 10.1186/s12933-014-0123-x.25106079PMC414927333

Chao
TF
, 
Leu
HB
, 
Huang
CC
, 
Chen
JW
, 
Chan
WL
, 
Lin
SJ
, 
Chen
SA
. Thiazolidinediones can prevent new onset atrial fibrillation in patients with non‐insulin dependent diabetes. International Journal of Cardiology. 2012;156:199–202. 10.1016/j.ijcard.2011.08.081.2193031534

Gu
J
, 
Liu
X
, 
Wang
X
, 
Shi
H
, 
Tan
H
, 
Zhou
L
, …
Wang
Y
. Beneficial effect of pioglitazone on the outcome of catheter ablation in patients with paroxysmal atrial fibrillation and type 2 diabetes mellitus. Europace. 2011;13(9):1256–1261. 10.1093/europace/eur131.2155147935

Otake
H
, 
Suzuki
H
, 
Honda
T
, 
Maruyama
Y
. Influences of autonomic nervous system on atrial arrhythmogenic substrates and the incidence of atrial fibrillation in diabetic heart. International Heart Journal. 2009;50(5):627–641. 10.1536/ihj.50.627.1980921136

Andrès
E
, 
Meyer
L
, 
Zulfiqar
AA
, 
Hajjam
M
, 
Talha
S
, 
Bahougne
T
, …
Hajjam El Hassani
A
. Telemonitoring in diabetes: Evolution of concepts and technologies, with a focus on results of the more recent studies. Journal of Medicine and Life. 2019;12:203–214.3166681810.25122/jml-2019-0006PMC681489037

Garabedian
LF
, 
Ross‐Degnan
D
, 
Wharam
JF
. Mobile phone and smartphone technologies for diabetes care and self‐management. Current Diabetes Reports. 2015;15:109.2645838010.1007/s11892-015-0680-8PMC652533138

Fokkert
MJ
, 
van Dijk
PR
, 
Edens
MA
, 
Abbes
S
, 
de Jong
D
, 
Slingerland
RJ
, 
Bilo
HJG
. Performance of the FreeStyle Libre Flash glucose monitoring system in patients with type 1 and 2 diabetes mellitus. BMJ Open Diabetes Research & Care. 2017;5(1):e000320. 10.1136/bmjdrc-2016-000320.PMC53169122824344939

Fleming
GA
, 
Petrie
JR
, 
Bergenstal
RM
, 
Holl
RW
, 
Peters
AL
, 
Heinemann
L
. Diabetes digital app technology: Benefits, challenges, and recommendations. A Consensus Report by the European Association for the Study of Diabetes (EASD) and the American Diabetes Association (ADA) Diabetes Technology Working Group. Diabetes Care. 2020;43:250–260. 10.2337/dci19-0062.3180664940

Veazie
S
, 
Winchell
K
, 
Gilbert
J
, 
Paynter
R
, 
Ivlev
I
, 
Eden
KB
, …
Helfand
M
. Rapid evidence review of mobile applications for self‐management of diabetes. Journal of General Internal Medicine. 2018;33:1167–1176.2974078610.1007/s11606-018-4410-1PMC602568041

Fokkert
M
, 
van Dijk
P
, 
Edens
M
, 
Barents
E
, 
Mollema
J
, 
Slingerland
R
, …
Bilo
H
. Improved well‐being and decreased disease burden after 1‐year use of flash glucose monitoring (FLARE‐NL4). BMJ Open Diabetes Research and Care. 2019;7:e000809. 10.1136/bmjdrc-2019-0008093.PMC69041653187513342

Liang
X
, 
Wang
Q
, 
Yang
X
, 
Cao
J
, 
Chen
J
, 
Mo
X
, …
Gu
D
. Effect of mobile phone intervention for diabetes on glycaemic control: A meta‐analysis. Diabetic Medicine. 2011;28:455–463.2139206610.1111/j.1464-5491.2010.03180.x43

Pillay
J
, 
Armstrong
MJ
, 
Butalia
S
, 
Donovan
LE
, 
Sigal
RJ
, 
Chordiya
P
, …
Dryden
DM
. Behavioral programs for type 1 diabetes mellitus: A systematic review and meta‐analysis. Annals of Internal Medicine. 2015;163:836–847.2641402010.7326/M15-139944

Saffari
M
, 
Ghanizadeh
G
, 
Koenig
HG
. Health education via mobile text messaging for glycemic control in adults with type 2 diabetes: A systematic review and meta‐analysis. Primary Care Diabetes. 2014;8:275–285.2479358910.1016/j.pcd.2014.03.00445

Agarwal
P
, 
Mukerji
G
, 
Desveaux
L
, 
Ivers
NM
, 
Bhattacharyya
O
, 
Hensel
JM
, …
Bhatia
RS
. Mobile app for improved self‐management of type 2 diabetes: Multicenter pragmatic randomized controlled trial. JMIR Mhealth and Uhealth. 2019;7:e10321.3063297210.2196/10321PMC632989646

Quinn
C
, 
Shardell
M
, 
Terrin
M
, 
Barr
EA
, 
Ballew
SH
, 
Gruber‐Baldini
AL
. Cluster‐randomized trial of a mobile phone personalized behavioral intervention for blood glucose control. Diabetes Care. 2011;34:1934–1942.2178863210.2337/dc11-0366PMC316130547

Pal
K
, 
Eastwood
SV
, 
Michie
S
, 
Farmer
A
, 
Barnard
ML
, 
Peacock
R
, …
Murray
E
. Computer‐based interventions to improve self‐management in adults with type 2 diabetes: A systematic review and meta‐analysis. Diabetes Care. 2014;37:1759–1766.2485515810.2337/dc13-138648

Whaley
CM
, 
Bollyky
JB
, 
Lu
W
, 
Painter
S
, 
Schneider
J
, 
Zhao
Z
, 
Meadows
ES
. Reduced medical spending associated with increased use of a remote diabetes management program and lower mean blood glucose values. Journal of Medical Economics. 2019;22:869–877.3101239210.1080/13696998.2019.160948349

Huxley
RR
, 
Lopez
FL
, 
Folsom
AR
, 
Agarwal
SK
, 
Loehr
LR
, 
Soliman
EZ
, …
Alonso
A
. Absolute and attributable risks of atrial fibrillation in relation to optimal and borderline risk factors: The Atherosclerosis Risk in Communities (ARIC) study. Circulation. 2011;123:1501–1508. 10.1161/CIRCULATIONAHA.110.009035.21444879PMC318149850

Bosworth
HB
, 
Powers
BJ
, 
Olsen
MK
, 
McCant
F
, 
Grubber
J
, 
Smith
V
, …
Oddone
EZ
. Home blood pressure management and improved blood pressure control: Results from a randomized controlled trial. Archives of Internal Medicine. 2011;171:1173–1180.2174701310.1001/archinternmed.2011.27651

Kim
Y‐N
, 
Shin
DG
, 
Park
S
, 
Lee
CH
. Randomized clinical trial to assess the effectiveness of remote patient monitoring and physician care in reducing office blood pressure. Hypertension Research. 2015;38:491–497.2578704110.1038/hr.2015.3252

McManus
RJ
, 
Mant
J
, 
Bray
EP
, 
Holder
R
, 
Jones
MI
, 
Greenfield
S
, …
Hobbs
FD
. Telemonitoring and self‐management in the control of hypertension (TASMINH2): A randomised controlled trial. Lancet. 2010;376:163–172.2061944810.1016/S0140-6736(10)60964-653

Duan
Y
, 
Xie
Z
, 
Dong
F
, 
Wu
Z
, 
Lin
Z
, 
Sun
N
, 
Xu
J
. Effectiveness of home blood pressure telemonitoring: A systematic review and meta‐analysis of randomised controlled studies. Journal of Human Hypertension. 2017;31:427–437.2833250610.1038/jhh.2016.9954

Tucker
KL
, 
Sheppard
JP
, 
Stevens
R
, 
Bosworth
HB
, 
Bove
A
, 
Bray
EP
, …
McManus
RJ
. Self‐monitoring of blood pressure in hypertension: A systematic review and individual patient data meta‐analysis. PLoS Medicine. 2017;14:e1002389.2892657310.1371/journal.pmed.1002389PMC560496555

McManus
RJ
, 
Mant
J
, 
Franssen
M
, 
Nickless
A
, 
Schwartz
C
, 
Hodgkinson
J
, …
Hobbs
FDR
. Efficacy of self‐monitored blood pressure, with or without telemonitoring, for titration of antihypertensive medication (TASMINH4): An unmasked randomised controlled trial. Lancet. 2018;391:949–959.2949987310.1016/S0140-6736(18)30309-XPMC585446356

Monahan
M
, 
Jowett
S
, 
Nickless
A
, 
Franssen
M
, 
Grant
S
, 
Greenfield
S
, …
McManus
RJ
. Cost‐effectiveness of telemonitoring and self‐monitoring of blood pressure for antihypertensive titration in primary care (TASMINH4). Hypertension. 2019;73:1231–1239.3106719010.1161/HYPERTENSIONAHA.118.12415PMC651040557

Williams
B
, 
Mancia
G
, 
Spiering
W
, 
Agabiti Rosei
E
, 
Azizi
M
, 
Burnier
M
, …
Desormais
I
. ESC Scientific Document Group. 2018 ESC/ESH Guidelines for the management of arterial hypertension. European Heart Journal. 2018;39:3021–3104.3016551610.1093/eurheartj/ehy33958

Daghlas
I
, 
Dashti
HS
, 
Lane
L
, 
Aragam
JL
, 
Rutter
MK
, 
Saxena
R
, 
Vetter
C
. Sleep duration and myocardial infarction. Journal of the American College of Cardiology. 2019;74:1304–1314.3148826710.1016/j.jacc.2019.07.022PMC678501159

Hirshkowitz
M
, 
Whiton
K
, 
Albert
SM
, 
Alessi
C
, 
Bruni
O
, 
DonCarlos
L
, …
Ware
JC
. National Sleep Foundation's updated sleep duration recommendations: Final report. Sleep Health. 2015;1:233–243.2907339810.1016/j.sleh.2015.10.00460

May
AM
, 
Blackwell
T
, 
Stone
PH
, 
Stone
KL
, 
Cawthon
PM
, 
Sauer
WH
, …
Mehra
R
. Central sleep‐disordered breathing predicts incident atrial fibrillation in older men. American Journal of Respiratory Critical Care Medicine. 2016;193:783–791.2659538010.1164/rccm.201508-1523OCPMC482493261

May
AM
, 
Van Wagoner
DR
, 
Mehra
R
. OSA and cardiac arrhythmogenesis: Mechanistic insights. Chest. 2017;151:225–241.2769359410.1016/j.chest.2016.09.014PMC598964362

Mehra
R
, 
Benjamin
EJ
, 
Shahar
E
, 
Gottlieb
DJ
, 
Nawabit
R
, 
Kirchner
HL
, 
Redline
S
. Sleep Heart Health Study. Association of nocturnal arrhythmias with sleep‐disordered breathing: The Sleep Heart Health Study. American Journal of Respiratory Critical Care Medicine. 2006;173:910–916.1642444310.1164/rccm.200509-1442OCPMC266290963
Institute of Medicine (US) Committee on Sleep Medicine and Research
; Colten HRAB,editors.Sleep Disorders and Sleep Deprivation: An Unmet Public HealthProblem. Washington,DC: National Academies Press (US); 2006. 3, Extent and Health Consequences of Chronic Sleep Loss and Sleep Disorders. Available at: http://www.ncbi.nlm.nih.gov/books/NBK19961/.2066943864

Burgess
HJ
, 
Trinder
J
, 
Kim
Y
, 
Luke
D
. Sleep and circadian influences on cardiac autonomic nervous system activity. The American Journal of Physiology. 1997;273:H1761–H1768.936224110.1152/ajpheart.1997.273.4.H176165

Qureshi
WT
, 
Nasir
UB
, 
Alqalyoobi
S
, 
O’Neal
WT
, 
Mawri
S
, 
Sabbagh
S
, 
Al‐Mallah
MH
. Meta‐analysis of continuous positive airway pressure as a therapy of atrial fibrillation in obstructive sleep apnea. American Journal of Cardiology. 2015;116(11):1767–1773. 10.1016/j.amjcard.2015.08.04.2648218266

Youssef
I
, 
Kamran
H
, 
Yacoub
M
, 
Patel
N
, 
Goulbourne
C
, 
Kumar
S
, 
Kane
J
, …
McFarlane
SI
. Obstructive sleep apnea as a risk factor for atrial fibrillation: A meta‐analysis. Journal of Sleep Disorders & Therapy. 2018;7:282. 10.4172/2167-0277.1000282.29657903PMC589840167

Khosla
S
, 
Deak
MC
, 
Gault
D
, 
Goldstein
CA
, 
Hwang
D
, 
Kwon
Y
, …
Rowley
JA
. American academy of sleep medicine board of directors. Consumer sleep technology: An American Academy of Sleep Medicine Position Statement. Journal of Clinical Sleep Medicine. 2018;14:877–880.2973499710.5664/jcsm.7128PMC594044068

Mantua
J
, 
Gravel
N
, 
Spencer
RM
. Reliability of sleep measures from four personal health monitoring devices compared to research‐based actigraphy and polysomnography. Sensors. 2016;16:646. 10.3390/s16050646.PMC48833372716411069

Selvaraj
N
, 
Narasimhan
R
. Automated prediction of the apnea‐hypopnea index using a wireless patch sensor. *Conference Proceedings IEEE Eng Med Biol Soc*, 2014;1897–900: 10.1109/EMBC.2014.6943981.2557034970

Huang
T
, 
Mariani
S
, 
Redline
S
. Sleep irregularity and risk of cardiovascular events: The multi‐ethnic study of atherosclerosis. Journal of the American College of Cardiology. 2020;75:991–999. 10.1016/j.jacc.2019.12.054.32138974PMC723795571

Berryhill
S
, 
Morton
CJ
, 
Dean
A
, 
Berryhill
A
, 
Provencio‐Dean
N
, 
Patel
SI
, …
Parthasarathy
S
. Effect of wearables on sleep in healthy individuals: A randomized crossover trial and validation study. Journal of Clinical Sleep Medicine. 2020;16:775–783.3204396110.5664/jcsm.8356PMC784981672

Everett
BM
, 
Conen
D
, 
Buring
JE
, 
Moorthy
MV
, 
Lee
IM
, 
Albert
CM
. Physical activity and the risk of incident atrial fibrillation in women. Circulation Cardiovascular Quality Outcomes. 2011;4:321–327.2148709210.1161/CIRCOUTCOMES.110.951442PMC309730773

Mozaffarian
D
, 
Furberg
CD
, 
Psaty
BM
, 
Siscovick
D
. Physical activity and incidence of atrial fibrillation in older adults – The cardiovascular health study. Circulation. 2008;118:800–807.1867876810.1161/CIRCULATIONAHA.108.785626PMC313395874

Piercy
KL
, 
Troiano
RP
, 
Ballard
RM
, 
Carlson
SA
, 
Fulton
JE
, 
Galuska
DA
, …
Olson
RD
. The physical activity guidelines for Americans. Journal of the American Medical Association. 2018;320:2020–2028.3041847110.1001/jama.2018.14854PMC958263175

Kramer
DB
, 
Mitchell
SL
, 
Monteiro
J
, 
Jones
PW
, 
Sharon‐Lise Normand
S‐L
, 
Hayes
DL
, …
Reynolds
MR
. Patient activity and survival following implantable cardioverter‐defibrillator implantation: The ALTITUDE activity study. Journal of the American Heart Association. 2015;4:e001775. 10.1161/JAHA.115.001775.25979902PMC459941076
IQUIVA Institute
. The growing value of digital health: Evidence and impact on human health and the healthcare system. 2017. Available at: https://www.iqvia.com/insights/the‐iqvia‐institute/reports/the‐growing‐value‐of‐digital‐health.77

McConnell
MV
, 
Turakhia
MP
, 
Harrington
RA
, 
King
AC
, 
Ashley
EA
. Mobile health advances in physical activity, fitness, and atrial fibrillation: Moving hearts. Journal of the American College of Cardiology. 2018;71:2691–2701.2988013010.1016/j.jacc.2018.04.03078

Faselis
C
, 
Kokkinos
P
, 
Tsimploulis
A
, 
Pittaras
A
, 
Myers
J
, 
Lavie
CJ
, …
Moore
H
. Exercise capacity and atrial fibrillation risk in veterans: A cohort study. Mayo Clinical Proceedings. 2016;91(5):558–566. 10.1016/j.mayocp.2016.03.002.2706867079

Pathak
RK
, 
Elliott
A
, 
Middeldorp
ME
, 
Meredith
M
, 
Mehta
AB
, 
Mahajan
R
, …
Sanders
P
. Impact of CARDIOrespiratory fitness on arrhythmia recurrence in obese individuals with atrial fibrillation: The CARDIO‐FIT study. Journal of the American College of Cardiology. 2015;66:985–966. 10.1016/j.jacc.2015.06.488.2611340680

Rosenberger
ME
, 
Buman
MP
, 
Haskell
WL
, 
McConnell
MV
, 
Carstensen
LL
. Twenty‐four hours of sleep, sedentary behavior, and physical activity with nine wearable devices. Medicine and Science in Sports and Exercise. 2016;4:457–465.10.1249/MSS.0000000000000778PMC47608802648495381

Sullivan
AN
, 
Lachman
ME
. Behavior change with fitness technology in sedentary adults: A review of the evidence for increasing physical activity. Frontiers in Public Health. 2017;4:289. 10.3389/fpubh.2016.00289.28123997PMC522512282

Jakicic
JM
, 
Davis
KK
, 
Rogers
RJ
, 
King
WC
, 
Marcus
MD
, 
Helsel
D
, …
Belle
SH
. Effect of wearable technology combined with a lifestyle intervention on long‐term weight loss: The IDEA randomized clinical trial. Journal of the American Medical Association. 2016;316:1161–1171.2765460210.1001/jama.2016.12858PMC548020983

Abdulla
J
, 
Nielsen
JR
. Is the risk of atrial fibrillation higher in athletes thanin the general population? A systematic review and meta‐analysis. Europace. 2009;11:1156–1159. 10.1093/europace/eup197.1963330584

Andersen
K
, 
Farahmand
B
, 
Ahlbom
A
, 
Held
C
, 
Ljunghall
S
, 
Michaelsson
K
, 
Sundstrom
J
. Risk of arrhythmias in 52 755 long‐distance crosscountry skiers: A cohort study. European Heart Journal. 2013;34:3624–3631. 10.1093/eurheartj/eht188.2375633285

Brunetti
ND
, 
Dellegrottaglie
G
, 
Di Giuseppe
G
, 
Lopriore
C
, 
Loiacono
T
, 
Gardini
G
, 
Patruno
S
, …
Di Biase
M
. Young football Italian amateur players remote electrocardiogram screening with telemedicine (you first) study: Preliminary results. International Journal of Cardiology. 2014;176:1257–1258.2512499710.1016/j.ijcard.2014.07.19586

Orchard
JJ
, 
Neubeck
L
, 
Orchard
JW
, 
Puranik
R
, 
Raju
H
, 
Freedman
B
, …
Semsarian
C
. ECG‐based cardiac screening programs: Legal, ethical, and logistical considerations. Heart Rhythm. 2019;16:1584–1591.3093033110.1016/j.hrthm.2019.03.02587

Aroganam
G
, 
Manivannan
N
, 
Harrison
D
. Review on wearable technology sensors used in consumer sport applications. Sensors. 2019;19:1983. 10.3390/s19091983.PMC65402703103533388

Li
RT
, 
Kling
SR
, 
Salata
MJ
, 
Cupp
SA
, 
Sheehan
J
, 
Voos
JE
. Wearable performance devices in sports medicine. Sports Health. 2016;8:74–78.2673359410.1177/1941738115616917PMC470215989

Peake
JM
, 
Kerr
G
, 
Sullivan
JP
. A Critical review of consumer wearables, mobile applications, and equipment for providing biofeedback, monitoring stress, and sleep in physically active populations. Frontiers in Physiology. 2018;9:743. 10.3389/fphys.2018.00743.30002629PMC603174690

Peart
DJ
, 
Balsalobre‐Fernández
C
, 
Shaw
MP
. Use of mobile applications to collect data in sport, health, and exercise science: A narrative review. The Journal of Strength & Conditioning Research. 2019;33:1167–1177.2917638410.1519/JSC.000000000000234491

Seshadri
DR
, 
Li
RT
, 
Voos
JE
, 
Rowbottom
JR
, 
Alfes
CM
, 
Zorman
CA
, 
Drummond
CK
. Wearable sensors for monitoring the internal and external workload of the athlete. NPJ Digital Medicine. 2019;2:71.3137250610.1038/s41746-019-0149-2PMC666280992

Coppetti
T
, 
Brauchlin
A
, 
Müggler
S
, 
Attinger‐Toller
A
, 
Templin
C
, 
Schönrath
F
, …
Wyss
CA
. Accuracy of smartphone apps for heart rate measurement. European Journal of Preventive Cardiology. 2017;24:1287–1293.2846470010.1177/204748731770204493

Dobbs
WC
, 
Fedewa
MV
, 
MacDonald
HV
, 
Holmes
CJ
, 
Cicone
ZS
, 
Plews
DJ
, 
Esco
MR
. The accuracy of acquiring heart rate variability from portable devices: A systematic review and meta‐analysis. Sports Medicine. 2019;2019(49):417–435. 10.1007/s40279-019-01061-5.3070623494

Singh
N
, 
Moneghetti
KJ
, 
Christle
JW
, 
Hadley
D
, 
Froelicher
V
, 
Plews
D
. Heart rate variability: An old metric with new meaning in the era of using mhealth technologies for health and exercise training guidance. Part two: Prognosis and training. Arrhythmia & Electrophysiology Review. 2018;7:247–255.3058831210.15420/aer.2018.30.2PMC630479395

Folsom
AR
, 
Yatsuya
H
, 
Nettleton
JA
, 
Lutsey
PL
, 
Cushman
M
, 
Rosamond
WD
, 
Investigators
AS
. Community prevalence of ideal cardiovascular health, by the American Heart Association definition, and relationship with cardiovascular disease incidence. Journal of the American College of Cardiology. 2011;57:1690–1696.2149276710.1016/j.jacc.2010.11.041PMC309304796

Abed
HS
, 
Wittert
GA
, 
Leong
DP
, 
Shirazi
MG
, 
Bahrami
B
, 
Middeldorp
ME
, …
Sanders
P
. Effect of weight reduction and cardiometabolic risk factor management on symptom burden and severity in patients with atrial fibrillation a randomized clinical trial. Journal of the American Medical Association. 2013;310:2050–2060.2424093210.1001/jama.2013.28052197

Donnellan
E
, 
Wazni
O
, 
Kanj
M
, 
Hussein
A
, 
Baranowski
B
, 
Lindsay
B
, …
Saliba
W
. Outcomes of atrial fibrillation ablation in morbidly obese patients following bariatric surgery compared with a nonobese cohort. Circulation: Arrhythmia and Electrophysiology. 2019;12:e007598.3161069310.1161/CIRCEP.119.00759898

Pathak
RK
, 
Middeldorp
ME
, 
Meredith
M
, 
Mehta
AB
, 
Mahajan
R
, 
Sanders
P
. Long‐term effect of goal‐directed weight management in an atrial fibrillation cohort a long‐term follow‐up study (LEGACY). Journal of the American College of Cardiology. 2015;65:2159–2169.2579236110.1016/j.jacc.2015.03.00299

El‐Gayar
O
, 
Timsina
P
, 
Nawar
N
, 
Eid
W
. Mobile applications for diabetes self‐management: Status and potential. Journal of Diabetes Science and Technology. 2013;7:247–262.2343918310.1177/193229681300700130PMC3692239100

Griffiths
C
, 
Harnack
L
, 
Pereira
MA
. Assessment of the accuracy of nutrient calculations of five popular nutrition tracking applications. Public Health Nutrition. 2018;21:1495–1502.2953477110.1017/S1368980018000393PMC10261454101

Boushey
CJ
, 
Spoden
M
, 
Zhu
FM
, 
Delp
EJ
, 
Kerr
DA
. New mobile methods for dietary assessment: Review of image‐assisted and image‐based dietary assessment methods. The Proceedings of the Nutrition Society. 2017;76:283–294.2793842510.1017/S0029665116002913102

Six
BL
, 
Schap
TE
, 
Zhu
FQM
, 
Mariappan
A
, 
Bosch
M
, 
Delp
EJ
, …
Boushey
CJ
. Evidence‐based development of a mobile telephone food record. Journal of the American Dietetic Association. 2010;110:74–79.2010283010.1016/j.jada.2009.10.010PMC3042797103

Zhu
FQ
, 
Bosch
M
, 
Woo
I
, 
Kim
S
, 
Boushey
CJ
, 
Ebert
DS
, 
Delp
EJ
. The use of mobile devices in aiding dietary assessment and evaluation. IEEE Journal of Selected Topics in Signal Processing. 2010;4:756–766.2086226610.1109/JSTSP.2010.2051471PMC2941896104

Fang
S
, 
Liu
C
, 
Zhu
F
, 
Delp
EJ
, 
Boushey
CJ
.Single‐view food portion estimation based on geometric models. 2015. *ISM: IEEE International Symposium on Multimedia: Proceedings IEEE International Symposiumon Multimedia*, *2015*, 3
8
5–3
90.10.1109/ISM.2015.67PMC503527427672682105

Dounavi
K
, 
Tsoumani
O
. Mobile health applications in weight management: A systematic literature review. American Journal of Preventive Medicine. 2019;56:894–903.3100380110.1016/j.amepre.2018.12.005106

Angaran
P
, 
Mariano
Z
, 
Dragan
V
, 
Zou
L
, 
Atzema
CL
, 
Mangat
I
, 
Dorian
P
. The atrial fibrillation therapies after ER visit: Outpatient care for patients with acute AF: the AFTER3 study. Journal of Atrial Fibrillation. 2015;7:1187.2795715010.4022/jafib.1187PMC5135218107

Lin
JS
, 
O’Connor
EA
, 
Evans
CV
, 
Senger
CA
, 
Rowland
MG
, 
Groom
HC
.Behavioral Counseling to Promote a Healthy Lifestyle for Cardiovascular Disease Prevention in Persons With Cardiovascular Risk Factors: An Updated Systematic Evidence Review for the U.S. Preventive Services TaskForce [Internet]. Rockville,MD: Agency for Healthcare Research and Quality(US); 2014. U.S. Preventive Services Task Force Evidence Syntheses, formerly Systematic Evidence Reviews. ReportNo.: 13‐05179‐EF‐1.25232633108

Hendriks
JM
, 
de Wit
R
, 
Crijns
HJ
, 
Vrijhoef
HJ
, 
Prins
MH
, 
Pisters
R
, 
Pison
LA
, 
Blaauw
Y
, 
Tieleman
RG
. Nurseled care vs. usual care for patients with atrial fibrillation: results of a randomized trial of integrated chronic care vs. routine clinical care in ambulatory patients with atrial fibrillation. European Heart Journal. 2012;33(21):2692–2699. 10.1093/eurheartj/ehs071.22453654109
U.S. Department of Veterans Affairs
. VA to provide capability for veterans to access their VA health data on Apple iPhones. Available at: https://www.va.gov/opa/pressrel/pressrelease.cfm?id55199.110

Chow
CK
, 
Ariyarathna
N
, 
Islam
SM
, 
Thiagalingam
A
, 
Redfern
J
. mHealth in cardiovascular health care. Heart Lung & Circulation. 2016;25:802–807.10.1016/j.hlc.2016.04.00927262389111

Walsh
JA
3rd
, 
Topol
EJ
, 
Steinhubl
SR
. Novel wireless devices for cardiac monitoring. Circulation. 2014;130:573–581.2511418610.1161/CIRCULATIONAHA.114.009024PMC4135373112

Slotwiner
DJ
, 
Tarakji
KG
, 
Al‐Khatib
SM
, 
Passman
RS
, 
Saxon
LA
, 
Peters
NS
, …
Marrouche
NF
. Transparent sharing of digital health data: A call to action. Heart Rhythm. 2019;16:e95–e106. 10.1016/j.hrthm.2019.04.042.31077802113

Coorey
GM
, 
Neubeck
L
, 
Mulley
J
, 
Redfern
J
. Effectiveness, acceptability and usefulness of mobile applications for cardiovascular disease self‐management: Systematic review with meta‐synthesis of quantitative and qualitative data. European Journal of Preventive Cardiology. 2018;25:505–521.2931336310.1177/2047487317750913114

Gandhi
S
, 
Chen
S
, 
Hong
L
, 
Sun
K
, 
Gong
E
, 
Li
C
, 
Yan
LL
, 
Schwalm
JD
. Effect of mobile health interventions on the secondary prevention of cardiovascular disease: Systematic review and meta‐analysis. Canadian Journal of Cardiology. 2017;33:219–231.10.1016/j.cjca.2016.08.01727956043115

Pfaeffli
DL
, 
Dobson
R
, 
Whittaker
R
, 
Maddison
R
. The effectiveness of mobile‐health behaviour change interventions for cardiovascular disease self‐management: A systematic review. European Journal of Preventive Cardiology. 2016;23:801–817.2649009310.1177/2047487315613462116

Hagglund
E
, 
Lynga
P
, 
Frie
F
, 
Ullman
B
, 
Persson
H
, 
Melin
M
, 
Hagerman
I
. Patient‐centred home‐based management of heart failure. Findings from a randomised clinical trial evaluating a tablet computer for self‐care, quality of life and effects on knowledge. Scandinavian Cardiovascular Journal. 2015;49:193–199.2596896810.3109/14017431.2015.1035319117

Varnfield
M
, 
Karunanithi
M
, 
Lee
CK
, 
Honeyman
E
, 
Arnold
D
, 
Ding
H
, 
Smith
C
, 
Walters
DL
. Smartphone‐based home care model improved use of cardiac rehabilitation in postmyocardial infarction patients: Results from a randomised controlled trial. Heart. 2014;100:1770–1779.2497308310.1136/heartjnl-2014-305783118

Kotecha
D
, 
Chua
WWL
, 
Fabritz
L
, 
Hendriks
J
, 
Casadei
B
, 
Schotten
U
, …
Kirchhof
P
. European Society of Cardiology (ESC) Atrial Fibrillation Guidelines Taskforce, the CATCH ME consortium and the European Heart Rhythm Association (EHRA). European Society of Cardiology smartphone and tablet applications for patients with atrial fibrillation and their health care providers. Europace. 2018;20:225–233. 10.1093/europace/eux299.29040548PMC5834097119

Guo
Y
, 
Chen
Y
, 
Lane
DA
, 
Liu
L
, 
Wang
Y
, 
Lip
GYH
. Mobile health technology for atrial fibrillation management integrating decision support, education and patient involvement: mAF App trial. The American Journal of Medicine. 2017;130:1388–1396.e6. 10.1016/j.amjmed.2017.07.003.28847546120

Rumsfeld
JS
, 
Alexander
KP
, 
Goff
DC
Jr
, 
Graham
MM
, 
Ho
PM
, 
Masoudi
FA
, …
Zerwic
JJ
. American Heart Association Council on Quality of Care and Outcomes Research, Council on Cardiovascular and Stroke Nursing, Council on Epidemiology and Prevention, Council on Peripheral Vascular Disease, and Stroke Council. Cardiovascular Health: The Importance of Measuring Patient‐Reported Health Status A Scientific Statement From the American Heart Association. Circulation. 2013;127:2233–2249. 10.1161/CIR.0b013e3182949a2e.23648778121

Varma
N
, 
Piccini
JP
, 
Snell
J
, 
Fischer
A
, 
Dalal
N
, 
Mittal
S
. The relationship between level of adherence to automatic wireless remote monitoring and survival in pacemaker and defibrillator patients. Journal of the American College of Cardiology. 2015;65:2601–2610. 10.1016/j.jacc.2015.04.033.25983008122

Chow
CK
, 
Redfern
J
, 
Hillis
GS
, 
Thakkar
J
, 
Santo
K
, 
Hackett
ML
, …
Thiagalingam
A
. Effect of lifestyle‐focused text messaging on risk factor modification in patients with coronary heart disease: A randomized clinical trial. Journal of the American Medical Association. 2015;314:1255–1263.2639384810.1001/jama.2015.10945123

Nahum‐Shani
I
, 
Smith
SN
, 
Spring
BJ
, 
Collins
LM
, 
Witkiewitz
K
, 
Tewari
A
, 
Murphy
SA
. Just‐in‐Time adaptive interventions (JITAIs) in mobile health: Key components and design principles for ongoing health behavior support. Annals of Behavioral Medicine. 2018;52:446–462.2766357810.1007/s12160-016-9830-8PMC5364076124

Gustafson
DH
, 
McTavish
FM
, 
Chih
MY
, 
Atwood
AK
, 
Johnson
RA
, 
Boyle
MG
, …
Shah
D
. A smartphone application to support recovery from alcoholism: A randomized clinical trial. Journal of the American Medical Association Psychiatry. 2014;71:566–572.2467116510.1001/jamapsychiatry.2013.4642PMC4016167125

Patrick
K
, 
Raab
F
, 
Adams
MA
, 
Dillon
L
, 
Zabinski
M
, 
Rock
CL
, 
Griswold
WG
, …
Norman
GJ
. A text message‐based intervention for weight loss: Randomized controlled trial. Journal of Medical Internet Research. 2009;11:e1.1914143310.2196/jmir.1100PMC2729073126

Riley
W
, 
Obermayer
J
, 
Jean‐Mary
J
. Internet and mobile phone text messaging intervention for college smokers. Journal of American College Health. 2008;57:245–248.1880954210.3200/JACH.57.2.245-248127

Nahum‐Shani
I
, 
Hekler
EB
, 
Spruijt‐Metz
D
. Building health behavior models to guide the development of just‐in‐time adaptive interventions: A pragmatic framework. Health Psychology. 2015;34s:1209–1219.2665146210.1037/hea0000306PMC4732268128

Park
LG
, 
Beatty
A
, 
Stafford
Z
, 
Whooley
MA
. Mobile phone interventions for the secondary prevention of cardiovascular disease. Progress in Cardiovascular Diseases. 2016;58:639–650.2700124510.1016/j.pcad.2016.03.002PMC4904827129

Coorey
GM
, 
Neubeck
L
, 
Mulley
J
, 
Redfern
J
. Effectiveness, acceptability and usefulness of mobile applications for cardiovascular disease self‐management: Systematic review with meta‐synthesis of quantitative and qualitative data. European Journal of Preventive Cardiology. 2018;25:505–521.2931336310.1177/2047487317750913130

Pfaeffli
DL
, 
Dobson
R
, 
Whittaker
R
, 
Maddison
R
. The effectiveness of mobile‐health behaviour change interventions for cardiovascular disease self‐management: A systematic review. European Journal of Preventive Cardiology. 2016;23:801–817.2649009310.1177/2047487315613462131

Blondon
K
, 
Meyer
P
, 
Lovis
C
, 
Ehrler
F
. Gamification and mHealth: A model to bolster cardiovascular disease self‐management. Swiss Medical Informatics. 2017;33(00):00398: 10.4414/smi.33.00398.132

Cugelman
B
. Gamification: What it is and why it matters to digital health behavior change developers. JMIR Serious Games. 2013;1:e3.2565875410.2196/games.3139PMC4307817133

Edwards
EA
, 
Lumsden
J
, 
Rivas
C
, 
Steed
L
, 
Edwards
LA
, 
Thiyagarajan
A
, …
Walton
RT
. Gamification for health promotion: Systematic review of behaviour change techniques in smartphone apps. British Medical Journal Open. 2016;6:e012447.10.1136/bmjopen-2016-012447PMC507362927707829134

Johnson
D
, 
Deterding
S
, 
Kuhn
KA
, 
Staneva
A
, 
Stoyanov
S
, 
Hides
L
. Gamification for health and wellbeing: A systematic review of the literature. Internet Interventions. 2016;2016(6):89–106.10.1016/j.invent.2016.10.002PMC609629730135818

## 5 PATIENT SELF‐MANAGEMENT—INTEGRATED CHRONIC CARE

Generally, structured management programs inclusive of intensive patient education may improve outcomes.^106^, ^107^, ^108^ These may be facilitated by mHealth.

### 5.1 Patient engagement

mHealth offers the opportunity to reach more patients more effectively. It may promote patient engagement through ease of access and wider dissemination to regions and communities who may not access health care through traditional modes due to cost, time, distance, embarrassment/stigma, marginalized groups, health inequities, etc.^329^ In this way, mHealth may facilitate information sharing and interaction between patients and HCPs without the need for an elaborate infrastructure (Figure 6).^110^, ^111^ Apps may aid HCPs to explain the condition and treatment options, utilizing videos, avatars, and individualized risk scores, enabling greater patient understanding and encouraging a two‐way exchange of information to achieve a concordant decision about treatment.

#### 5.1.1 Patients' access to their own health data

A recent HRS statement advocates for transparent and secure access by patients to their digital data.^332^ This enables active participation and appropriate self‐management. For instance, many patients with AF are interested in seeing their AF burden and physiologic data, similarly to patients with hypertension tracking their BP or patients with diabetes tracking their glucose. Recent systematic reviews of technology‐based patient‐directed interventions for cardiovascular disease suggest that engaging elements include self‐monitoring of symptoms and measurements, daily tracking of health behaviors, disease education, reminders, and interaction with HCPs.^104^, ^113^, ^114^, ^115^ In some cardiovascular conditions, self‐management (without any HCP input) improved key outcomes.^336,337^

The model requires that patients assume responsibility and accountability for tracking conditions effectively and taking corrective measures. Possibly, this may be facilitated by data organization to present salient elements in a format comprehensible to the lay public. Active role of patients in decision‐making regarding the choice of treatment has been underlined by AF clinical guidance documents. Patients with AF are encouraged to be involved in decision‐taking through better understanding of their disease, which helps to improve communication between patients, their families, and doctors and improves patients' adherence to prescribed therapy. Two applications in AF—one for patients and the other for healthcare providers—have been developed by CATCH ME Consortium in collaboration with European Society of Cardiology,^338^ but these have yet to be formally tested. In China, Guo and colleagues^339^ demonstrated that the mobile atrial fibrillation (mAFA) app, incorporating decision support, education, and patient engagement, significantly improved AF patients' knowledge, medication adherence, quality of life, and satisfaction to anticoagulation compared to usual care.

Limitations should be recognized:

- Demands of self‐management may be excessive for even well intentioned patients required to be facile with setting up their own medical monitoring device, assessing frequency of download, interpreting and acting on data when required, and troubleshooting. These are not trivial challenges.

### 5.2 Behavioral modification

Individual health status has been found to be a strong independent predictor of mortality and cardiovascular events.^340^ mHealth may catalyze positive behavioral change and facilitate health care. An induced healthy‐user effect was likely the basis of survival benefit among CIED patients adhering more closely to remote management.^341^ mHealth may support patients with text messaging^342^ or mobile applications to remind patients of medication doses and times, as well as medical appointments (but synchronization with healthcare providers and/or EMR is generally lacking). The “just‐in‐time adaptive intervention” (JITAI) premise is to provide the appropriate type and amount of support to an individual at the correct time, with the ability to adjust depending on the person’s current internal and situational factors.^343^ mHealth technology is an ideal platform to facilitate JITAIs by providing “real‐time” personalized information, which can be utilized to inform the intervention delivered. JITAIs have been widely employed for health promotion and to support behavior change, but evidence of their efficacy is limited.^124^, ^125^, ^126^ Timing is integral to the perception of benefit, as is receptivity to accept and use the support.^347^ Bespoke, multi‐faceted mHealth tools, with motivational messages and incorporating gamification, are most engaging.^114^, ^128^, ^129^, ^130^

Incorporation of gamification strategies (e.g., rewards, prizes, avatars, performance feedback, leader‐boards, competitions, and social connection) into mHealth promotes patient engagement and sustains healthy behaviors.^131^, ^132^, ^133^, ^134^, ^135^ However, a recent systematic review demonstrated that only 4% (64/1680) of English‐language “top‐rated” health apps incorporated ≥1 gaming feature.^353^ There are limited hypothesis‐generated data for these mHealth interventions, and their efficacy in this context is as yet unmeasured. Self‐regulatory behavior change techniques, such as feedback and monitoring (including self‐monitoring), comparison of behavior, rewards, incentives and threats, and social support, are the most common behavior change techniques employed in gamification apps and are frequently utilized in successful non‐gaming apps targeting health promotion and secondary prevention.^133^, ^136^, ^137^ Engaging with apps involving gamification can also improve emotional well‐being through feelings of accomplishment and social connectivity.^354^

### 5.3 Patients as part of a community

Incorporation of a patient as part of a wider community may offer benefits. Social networking is widely used for health.^358^ Online communities enable individuals to “meet,” share their experiences, discuss treatment, and receive and provide support from peers, patient organizations, or HCPs.^138^, ^139^, ^140^ While crowdsourcing via the Internet and social networks allows collective sharing and exchange of information from a large number of people, the integrity and accuracy of such information remains largely un‐vetted and as such may be unreliable.^361^

### 5.4 Maintaining patient engagement

Sustaining healthy behaviors and minimizing intervention fatigue is paramount to long‐term maintenance. Although mHealth may help to maintain motivation, available data demonstrate significant attrition with mHealth interventions targeting risk factors and chronic conditions, even when people report liking the intervention and have purchased it,^142^, ^143^, ^144^, ^145^, ^146^, ^147^, ^148^, ^149^, ^150^ webEndevaour, Apple Heart Study).

A representative patient’s experience is described below:

A few years ago (2017), a friend told me about a new app that he had installed on his iPhone that would allow him to measure his heart rate through a fingertip pulse. Having an irregular heartbeat, under control through medication, I was very interested to try the new app. I thought it would provide me the opportunity to know more about myself, specifically how my heart operated under stress and at different times of day, before, during, and after physical exertion of a variety of my favorite sports and pastimes like tennis, golf, biking, and fly fishing.

At first, I was quite satisfied with the rudimentary calculations. Then, I noticed during my international business travels that the device was often down during US nighttime hours during which time I thought the ‘hosts’ were making repairs or improvements. I also noticed that there were several radically incorrect readings especially during early morning hours. It simply wasn’t performing up to the standards of more traditional monitoring devices. I found as well that the host’s increasing attempt to up‐sell to premium packages and other online health management tools became quite burdensome.

Before long, I felt almost addicted to the device and ultimately quit on it altogether. In retrospect, I believe that if I had had a proper introduction to the device by a trained medical specialist, I might have had a different expectation of this online tool, how to use it and how to interpret its data output.

Understanding the basis for health‐protective behavior is vital.^371^ Many apps, including those from national heart foundations [*websites*],^152^, ^153^, ^154^ are available to support healthy lifestyle choices, but their efficacy remains largely untested or is limited by design features (i.e., small sample sizes, selection bias, etc.). Cost, service connectivity, and credibility of information sources are important factors. However, patient engagement may be jeopardized by worries about privacy and personal data security.^110^, ^155^, ^156^, ^157^

#### 5.4.1 Continued clinic support

The level and duration of clinic support needed will likely depend on condition monitored and goals for treatment. Reduction in compulsory routine in‐clinic evaluations and reliance on continuous remote monitoring improved retention to long‐term follow‐up of patients with CIEDs.^378^ In one HF trial, gain was related to the period of remote instruction. Whether this indicates that efficacy of the active program had peaked and stabilized or that it needed to be sustained is unclear. 379 Ideally, a training program should be finite in time but its effects durable.

### 5.5 Digital divide

Although mHealth is highly promising in transforming health care, it can potentially exacerbate disparities in health care along sociodemographic lines.

Older people are perceived to engage less with mHealth. A 2017 Pew Research Center survey found that 92% of 18‐29 year olds and 74% of age 50‐64 year olds own a smartphone.^380^ However, the lack of familiarity with the technology and access to mobile devices, rather than lack of engagement *per se*, remain the principal barriers.^161^, ^162^, ^163^ Older users of mHealth prefer personalized information, which is clearly presented and is easy to navigate.^384^

There is also disparity across the educational spectrum, with smartphone usage in 57% of the population with less than high school education and 91% of the population who graduated from college.

Smartphone use differs by income, with smartphone usage in 67% of the population with income annual ≤$30,000 and 93% of the population with income ≥$75,000.^385^ Limited evidence from the USA suggests that, although there is some variation in the mHealth use related to ethnicity, black and Hispanic Americans are not disadvantaged.^386^ mHealth permits information and apps to be tailored appropriately for language, literacy levels (including “text to speech” technology), and cultural differences to promote engagement.^161^, ^167^, ^168^

There is heterogeneity of mHealth availability among different countries.^389^ Even some of the best studied and FDA and CE approved technologies described here may be currently unavailable due to regulatory or marketing rules or simply unaffordable to either individuals or healthcare systems in many other countries.

As healthcare systems leverage and incorporate smartphone‐based technology in their workflow and processes, a strategy is needed in parallel to ensure that those who do not have access to smartphone‐based technology will continue to receive appropriate high‐quality care. This critical initiative will require consensus and action among all stakeholders including HCPs, hospital systems, insurance providers, and state and federal government agencies. Thus enabled, mHealth promises improved patient outcomes in resource‐limited areas.^390^

REFERENCES SECTION 5135

Sardi
L
, 
Idri
A
, 
Fernandez‐Aleman
JL
. A systematic review of gamification in e‐Health. Journal of Biomedical Informatics. 2017;71:31–48.2853606210.1016/j.jbi.2017.05.011136

Conroy
DE
, 
Yang
CH
, 
Maher
JP
. Behavior change techniques in top‐ranked mobile apps for physical activity. American Journal of Preventive Medicine. 2014;46:649–652.2484274210.1016/j.amepre.2014.01.010137

Direito
A
, 
Dale
LP
, 
Shields
E
, 
Dobson
R
, 
Whittaker
R
, 
Maddison
R
. Do physical activity and dietary smartphone applications incorporate evidence‐based behaviour change techniques?
BMC Public Health. 2014;14:646.2496580510.1186/1471-2458-14-646PMC4080693138

Fox
S
.The Social Life of Health Information. 2011. Available at: https://www.pewresearch.org/fact‐tank/2014/01/15/the‐social‐life‐of‐health‐information/.139

Swan
M
. Emerging patient‐driven health care models: An examination of health social networks, consumer personalized medicine and quantified self‐tracking. International Journal of Environmental Research and Public Health. 2009;6:492–525.1944039610.3390/ijerph6020492PMC2672358140

Swan
M
. Health 2050: The realization of personalized medicine through crowdsourcing, the quantified self, and the participatory biocitizen. Journal of Personalized Medicine. 2012;2:93–118.2556220310.3390/jpm2030093PMC4251367141

Besaleva
LI
, 
Weaver
AC
. CrowdHelp: m‐Health application for emergency response improvement through crowdsourced and sensor‐detected information. 2014. Available at: https://ieeexplore.ieee.org/document/6693335.142

Chaudhry
SI
, 
Mattera
JA
, 
Curtis
JP
, 
Spertus
JA
, 
Herrin
J
, 
Lin
Z
, …
Krumholz
HM
. Telemonitoring in patients with heart failure. New England Journal of Medicine. 2010;363:2301–2309.10.1056/NEJMoa1010029PMC323739421080835143

Flores Mateo
G
, 
Granado‐Font
E
, 
Ferre‐Grau
C
, 
Ferré‐Grau
C
, 
Montaña‐Carreras
X
. Mobile phone apps to promote weight loss and increase physical activity: A systematic review and meta‐analysis. Journal of Medical Internet Research. 2015;17:e253.2655431410.2196/jmir.4836PMC4704965144

Fukuoka
Y
, 
Gay
C
, 
Haskell
W
, 
Arai
S
, 
Vittinghoff
E
. Identifying factors associated with dropout during prerandomization run‐in period from an mhealth physical activity education study: The mPED trial. JMIR Mhealth Uhealth. 2015;3:e34.2587275410.2196/mhealth.3928PMC4411363145

Morgan
JM
, 
Kitt
S
, 
Gill
J
, 
McComb
JM
, 
Ng
GA
, 
Raftery
J
, 
Cowie
MR
. Remote management of heart failure using implantable electronic devices. European Heart Journal. 2017;38:2352–2360.2857523510.1093/eurheartj/ehx227PMC5837548146

Owen
JE
, 
Jaworski
BK
, 
Kuhn
E
, 
Makin‐Byrd
KN
, 
Ramsey
KM
, 
Hoffman
JE
. mHealth in the wild: Using novel data to examine the reach, use, and impact of PTSD coach. JMIR Mental Health. 2015;2:e7.2654391310.2196/mental.3935PMC4607374147

Simblett
S
, 
Greer
B
, 
Matcham
F
, 
Curtis
H
, 
Polhemus
A
, 
Ferrão
J
, 
Gamble
P
, 
Wykes
T
. Barriers to and facilitators of engagement with remote measurement technology for managing health: Systematic review and content analysis of findings. Journal of Medical Internet Research. 2018;20:e10480.3000199710.2196/10480PMC6062692148

Whitehead
L
, 
Seaton
P
. The effectiveness of self‐management mobile phone and tablet apps in long‐term condition management: A systematic review. Journal of Medical Internet Research. 2016;18:e97.2718529510.2196/jmir.4883PMC4886099149
Canadian Heart and Stroke Foundation
. Heart and stroke etools for a healthier you. 2014. Available at: http://www.heartandstroke.com/site/c.ikIQLcMWJtE/b.8324551/k.972C/_30_days.htm?utm_campaign=offline&utm_source=30days&utm_medium=vanity.150
Endeavour Partners
. Inside wearables: How the science of human behavior change offers the secret to long‐term. 2017. Available at: https://medium.com/@endeavourprtnrs/inside‐wearable‐how‐the‐science‐of‐human‐behavior‐change‐offers‐the‐secret‐to‐long‐term‐engagement‐a15b3c7d4cf3.151

Dunton
GF
. Sustaining health‐protective behaviors such as physical activity and healthy eating. Journal of the American Medical Association. 2018;320:639–640.2985204610.1001/jama.2018.6621PMC7524543152
National Heart Foundation of Australia
. My heart, my life. 2014. Available at: https://myheartmylife.org.au/.153
American Heart Association
. Sustaining healthy behaviours (AHA Simple 7). 2019. Available at: https://www.heart.org/en/healthy‐living/healthy‐lifestyle/my‐life‐check‐lifes‐simple‐7.154
British Heart Foundation
. Our healthy recipe finder app. 2014. Available at: http://www.bhf.org.uk/heart‐health/prevention/healthy‐eating/our‐healthy‐recipe‐finder‐app.aspx.155

Burke
LE
, 
Ma
J
, 
Azar
KM
, 
Bennett
GG
, 
Peterson
ED
, 
Zheng
Y
, …
Quinn
CC
. Current science on consumer use of mobile health for cardiovascular disease prevention: A scientific statement from the American Heart Association. Circulation. 2015;132:1157–1213.2627189210.1161/CIR.0000000000000232PMC7313380156

Kumar
S
, 
Nilsen
WJ
, 
Abernethy
A
, 
Atienza
A
, 
Patrick
K
, 
Pavel
M
, …
Swendeman
D
. Mobile health technology evaluation: The mHealth evidence workshop. American Journal of Preventive Medicine. 2013;45:228–236.2386703110.1016/j.amepre.2013.03.017PMC3803146157

Steinhubl
SR
, 
Muse
ED
, 
Topol
EJ
. The emerging field of mobile health. Science Translational Medicine. 2015;7:283rv283.10.1126/scitranslmed.aaa3487PMC474883825877894158

Varma
N
, 
Michalski
J
, 
Stambler
B
, 
Pavri
BB
, 
Investigators
TRUST
. Superiority of automatic remote monitoring compared with in‐person evaluation for scheduled ICD follow‐up in the TRUST trial – Testing execution of the recommendations. European Heart Journal. 2014;35:1345–1352. 10.1093/eurheartj/ehu066.24595864PMC4028610159

Varma
N
. Remote patent management of heart failure patients – How long should it go on?
Lancet Digital Health. 2020;2. 10.1016/S2589-7500(19)30221-3.33328036160

Smith
A
. Record shares of Americans now own smartphones, have home broadband. Pew Research Center; 2017. Available at: https://www.pewresearch.org/fact‐tank/2017/01/12/evolution‐of‐technology/.161

Coorey
GM
, 
Neubeck
L
, 
Mulley
J
, 
Redfern
J
. Effectiveness, acceptability and usefulness of mobile applications for cardiovascular disease self‐manegement: Systematic review with meta‐synthesis of quantitative and qualitattive data. European Journal of Preventive Cardiology. 2018;25:505–521. 10.1177/2047487317750913.29313363162

Coorey
GM
, 
Neubeck
L
, 
Mulley
J
, 
Glinatsis
H
, 
Belshaw
J
, 
Kirkness
A
, …
Neubeck
L
. Mobile technology use across age groups in patients eligible for cardiac rehabilitation: Survey study. JMIR Mhealth Uhealth. 2017;5:e161.2906642510.2196/mhealth.8352PMC5676027163

Tarakji
KG
, 
Vives
CA
, 
Patel
AS
, 
Fagan
DH
, 
Sims
JJ
, 
Varma
N
. Success of pacemaker remote monitoring using app‐based technology: Does patient age matter?
Pacing and Clinical Electrophysiology. 2018;41:1329–1335.3005501310.1111/pace.13461164

Neubeck
L
, 
Lowres
N
, 
Benjamin
EJ
, 
Freedman
SB
, 
Coorey
G
, 
Redfern
J
. The mobile revolution–using smartphone apps to prevent cardiovascular disease. Nature Reviews. Cardiology. 2015;12:350–360.2580171410.1038/nrcardio.2015.34165
PR.C Mobile
. Mobile FactSheet. 2018. Available at: https://www.pewresearch.org/internet/fact‐sheet/mobile/.166

Martin
T
. Assessing mHealth: Opportunities and barriers to patient engagement. Journal of Health Care for the Poor and Underserved. 2012;23:935–941.2421214410.1353/hpu.2012.0087167

Neubeck
L
, 
Cartledge
S
, 
Dawkes
S
, 
Gallagher
R
. Is there an app for that? Mobile phones and secondary prevention of cardiovascular disease. Current Opinion Cardiology. 2017;32:567–571.10.1097/HCO.000000000000042828614104168

Redfern
J
, 
Santo
K
, 
Coorey
G
, 
Thakkar
J
, 
Hackett
M
, 
Thiagalingam
A
, 
Chow
CK
. Factors influencing engagement, perceived usefulness and behavioral mechanisms associated with a text message support program. PLoS One. 2016;11:e0163929.2774124410.1371/journal.pone.0163929PMC5065147169

Varma
N
. Remote patent management of heart failure patients – How long should it go on?
Lancet Digital Health. 2020;2(1):E2–E3. 10.1016/S2589-7500(19)30221-3.33328036170

Bhavnani
SP
, 
Sola
S
, 
Adams
D
, 
Venkateshvaran
A
, 
Dash
PK
, 
Sengupta
PP
, …
Kadakia
A
. A randomized trial of pocket‐echocardiography integrated mobile health device assessments in modern structural heart disease clinics. Cardiovascular Imaging. 2018;11:546–557. 10.1016/j.jcmg.2017.06.019.28917688171

Piccini
JP
, 
Clark
RL
, 
Kowey
PR
, 
Mittal
S
, 
Dunnmon
P
, 
Stockbridge
N
, …
Ismat
F
. Long‐term electrocardiographic safety monitoring in clinical drug development: A report from the Cardiac Safety Research Consortium. American Heart Journal. 2017;187:156–169.2845479910.1016/j.ahj.2017.01.012172

Andrade
JG
, 
Champagne
J
, 
Dubuc
M
, 
Deyell
MW
, 
Verma
A
, 
Macle
L
, …
Khairy
P
. Cryoballoon or radiofrequency ablation for atrial fibrillation assessed by continuous monitoring: A randomized clinical trial. Circulation. 2019;140:1779–1788.3163053810.1161/CIRCULATIONAHA.119.042622173

Nguyen
KT
, 
Olgin
JE
, 
Pletcher
MJ
, 
Ng
M
, 
Kaye
L
, 
Moturu
S
, …
Marcus
GM
. Smartphone‐based geofencing to ascertain hospitalizations. Circulation Cardiovascular Quality & Outcomes. 2017;10:e003326. 10.1161/CIRCOUTCOMES.116.003326.28325751PMC5363280174

Perez
MV
, 
Mahaffey
KW
, 
Hedlin
H
, 
Rumsfeld
JS
, 
Garcia
A
, 
Ferris
T
, …
Turakhia
MP
. Large‐scale assessment of a smartwatch to identify atrial fibrillation. New England Journal of Medicine. 2019;381:1909–1917. 10.1056/NEJMoa1901183.PMC811260531722151175

Perez
MV
, 
Mahaffey
KW
, 
Hedlin
H
, 
Rumsfeld
JS
, 
Garcia
A
, 
Ferris
T
, …
Turakhia
MP
. Large‐scale assessment of a smartwatch to identify atrial fibrillation. New England Journal of Medicine. 2019;381:1909–1917. 10.1056/NEJMoa1901183.PMC811260531722151176

Guo
Y
, 
Wang
H
, 
Zhang
H
, 
Liu
T
, 
Liang
Z
, 
Xia
Y
, …
Lip
GYH
. Mobile photoplethysmographic technology to detect atrial fibrillation. Journal of the American College of Cardiology. 2019;2019(74):2365–2375. 10.1016/j.jacc.2019.08.019.31487545177

Guo
Y
, 
Lane
DA
, 
Wang
L
, 
Zhang
H
, 
Wang
H
, 
Zhang
W
, …
Lip
GYH
. Mobile health technology to improve care for patients with atrial fibrillation. Journal of the American College of Cardiology. 2020;75:1523–1534.3224136710.1016/j.jacc.2020.01.052178

Passman
R
, 
Leong‐Sit
P
, 
Andrei
AC
, 
Huskin
A
, 
Tomson
TT
, 
Bernstein
R
, …
Zimetbaum
P
. Targeted anticoagulation for atrial fibrillation guided by continuous rhythm assessment with an insertable cardiac monitor: The rhythm evaluation for anticoagulation with continuous monitoring (REACT.COM) pilot study. Journal of Cardiovascular Electrophysiology. 2016;27:264–270. 10.1111/jce.12864.26511221PMC4789135179

Van Gelder
IC
, 
Groenveld
HF
, 
Crijns
HJ
, 
Tuininga
YS
, 
Tijssen
JG
, 
Alings
AM
, …
Van den Berg
MP
. Lenient versus strict rate control in patients with atrial fibrillation. New England Journal of Medicine. 2010;362:1363–1373. 10.1056/NEJMoa1001337.20231232180

Guo
X
, 
Vittinghoff
E
, 
Olgin
JE
, 
Marcus
GM
, 
Pletcher
MJ
. Volunteer participation in the Health eHeart Study: A comparison with the US population. Science Reports. 2017;2017(7):1956. 10.1038/s41598-017-02232-y.PMC543403928512303181

Chen
LY
, 
Chung
MK
, 
Allen
LA
, 
Ezekowitz
M
, 
Furie
KL
, 
McCabe
P
, …
Turakhia
MP
. Atrial fibrillation burden: moving beyond atrial fibrillation as a binary entity: A scientific statement from the American Heart Association. Circulation. 2018;137:e623–e644.2966194410.1161/CIR.0000000000000568PMC8463258182

Glotzer
TV
, 
Daoud
EG
, 
Wyse
DG
, 
Singer
DE
, 
Ezekowitz
MD
, 
Hilker
C
, …
Ziegler
PD
. The relationship between daily atrial tachyarrhythmia burden from implantable device diagnostics and stroke risk: The TRENDS study. Circulation: Arrhythmia and Electrophysiology. 2009;2:474–480.1984391410.1161/CIRCEP.109.849638183

Piccini
JP
, 
Passman
R
, 
Turakhia
M
, 
Connolly
AT
, 
Nabutovsky
Y
, …
Varma
N
. Atrial fibrillation burden, progression, and the risk of death: A case‐crossover analysis in patients with cardiac implantable electronic devices. Europace. 2019;21:404–413.3046220810.1093/europace/euy222184

Wong
JA
, 
Conen
D
, 
Van Gelder
IC
, 
McIntyre
WF
, 
Crijns
HJ
, 
Wang
J
, …
Healey
JS
. Progression of device‐detected subclinical atrial fibrillation and the risk of heart failure. Journal of the American College of Cardiology. 2018;71:2603–2611.2988011910.1016/j.jacc.2018.03.519185

Varma
N
, 
Marrouche
NF
, 
Aguinaga
L
, 
Albert
CM
, 
Arbelo
E
, 
Choi
JI
, …
Varosy
PD
. HRS/EHRA/APHRS/LAHRS/ACC/AHA worldwide practical guidance for telehealth and arrhythmia monitoring during and after a pandemic. Journal of the American College of Cardiology. 2020. 10.1016/j.jacc.2020.06.019.PMC728908832534936186

Slotwiner
DJ
, 
Abraham
RL
, 
Al‐Khatib
SM
, 
Anderson
HV
, 
Bunch
TJ
, 
Ferrara
MG
, …
Wilkoff
BL
. HRS White Paper on interoperability of data from cardiac implantable electronic devices (CIEDs). Heart Rhythm. 2019;16:e107–e127. 10.1016/j.hrthm.2019.05.002.31077801187

Chow
CK
, 
Redfern
J
, 
Hillis
GS
, 
Thakkar
J
, 
Santo
K
, 
Hackett
ML
, …
Thiagalingam
A
. Effect of lifestyle‐focused text messaging on risk factor modification in patients with coronary heart disease: A randomized clinical trial. Journal of the American Medical Association. 2015;314:1255–1263.2639384810.1001/jama.2015.10945188

Spaulding
EM
, 
Marvel
FA
, 
Lee
MA
, 
Yang
WE
, 
Demo
R
, 
Wang
J
, …
Martin
SS
. Corrie health digital platform for self‐management in secondary prevention after acute myocardial infarction. Circulation. Cardiovascular Quality & Outcomes. 2019;12:e005509.3104306510.1161/CIRCOUTCOMES.119.005509PMC6697167189

Ratwani
RM
, 
Savage
E
, 
Will
A
, 
Arnold
R
, 
Khairat
S
, 
Miller
K
, …
Hettinger
AZ
. A usability and safety analysis of electronic health records: A multi‐center study. Journal of the American Medical Information Association. 2018;25:1197–1201.10.1093/jamia/ocy088PMC764687529982549190

Jalali
MS
, 
Russell
B
, 
Razak
S
, 
Gordon
WJ
. EARS to cyber incidents in health care. Journal of the American Medical Information Association. 2019;26:81–90.10.1093/jamia/ocy148PMC764715830517701191

Kruse
CS
, 
Frederick
B
, 
Jacobson
T
, 
Monticone
DK
. Cybersecurity in healthcare: A systematic review of modern threats and trends. Technology Health Care. 2017;25:1–10.2768956210.3233/THC-161263192

Mansfield‐Devine
S
. Ransomware: Taking businesses hostage. Network Security. 2016;8–17.193
Hospitals become major target for ransomware. Network Security. 2016;1–2. 10.1016/S1353-4858(16)30031-9.194

Perakslis
ED
. Cybersecurity in health care. New England Journal of Medicine. 2014;371:395–397.10.1056/NEJMp140435825075831195

Klonoff
DC
. Cybersecurity for connected diabetes devices. Journal of Diabetes Science and Technology. 2015;9:1143–1147.2588316210.1177/1932296815583334PMC4667325196

Shanon
CE
. Communication theory of secrecy systems'. Bell System Technical Journal. 1949;28.197

Gollakota
S
, 
Hassanieh
H
, 
Ransford
B
, 
Katabi
D
, 
Fu
K
. They can hear your heartbeats: Non‐invasive security for implantablemedical devices. 10.1145/2043164.2018438.198

Saxon
LA
, 
Varma
N
, 
Epstein
LM
, 
Ganz
LI
, 
Epstein
AE
. Factors influencing the decision to proceed to firmware upgrades to implanted pacemakers for cybersecurity risk mitigation. Circulation. 2018;138:1274–1276.2974818810.1161/CIRCULATIONAHA.118.034781199

Voelker
R
. FDA joins new effort to strengthen medical device cybersecurity. Journal of the American Medical Association. 2018;320:1970.10.1001/jama.2018.1783430458476200

Shuren
J
, 
Patel
B
, 
Gottlieb
S
. FDA regulation of mobile medical apps. Journal of the American Medical Association. 2018;320:337–338.2997133910.1001/jama.2018.8832

## 6 CLINICAL TRIALS

mHealth may have particular impact on trials of heart rhythm disorders. Traditionally, clinical trials testing drugs and devices for arrhythmias utilized time‐to‐event outcomes and analyses, such as first recurrence of AF after a blanking period.^391^ Patients randomized to the control and intervention would be monitored intermittently, either with ambulatory devices and/or in‐clinic visit. Such monitoring had limited sensitivity for recurrent arrhythmias, including symptomatic and asymptomatic episodes. Furthermore, time‐to‐first event may not accurately capture reductions in arrhythmia burden, which have also been shown to be beneficial in recent randomized trials.^392^ While CIEDs such as pacemakers and defibrillators can be leveraged for continuous monitoring,^146^ these studies do not generalize to broader CIED‐free populations. ILRs may have a potential role, but are costly and unless used for clinical indications, difficult to justify simply for study event ascertainment.

There are a variety of free‐standing handheld ECG monitors, some of which have automated AF detection (Table 1). However, many do not have cellular or networking capability and therefore generally cannot transmit data or findings in real time. This is where smart‐ or mobile‐connected arrhythmia and pulse detection technologies have significant promise. These may enhance detection and measurement of clinical outcomes while also allowing for remote or virtual data collection without the need for site‐based study visits. Examples include remote rhythm assessment with single‐ or multilead ECGs from smartphone or smartwatch‐based technologies and automatic ascertainment of hospitalizations using smartphone‐based geofencing.^393^ These operational enhancements, in turn, can improve participant satisfaction, reduce cost, improve study efficiency, and facilitate or expand enrollment. An example is the ongoing Health eHeart study, a site‐free cardiovascular research study that leverages self‐reported data, data from wearable sensors, electronic health records, and other importable “big data” to enable rapid‐cycle, low‐cost interventional and observational cardiovascular research (https://www.health‐eheartstudy.org/).

### 6.1 Screening

Two recent large‐scale studies highlight the potential advantages of mHealth for AF screening and treatment.

#### 6.1.1 The Apple heart study

This was a highly pragmatic, single‐arm investigational device exemption study designed to test the performance and safety of a PPG‐based irregular rhythm detection algorithm on the Apple Watch for identification of AF.^67^, ^174^ The study was a siteless “bring your own device” study, such that participants needed their own compatible smartphone and watch to enroll online. All study procedures, including eligibility verification, onboarding, enrollment, and data collection, were performed via the study app, which could be downloaded from the app store. If a participant received an irregular pulse notification, then subsequent study visits were done via video conferencing to study physicians directly with the app. The study enrolled over 419,000 participants without pre‐existing AF in just an eight‐month period, in large part due to the pragmatic, virtual design, and easy accessibility (Figure 4). The algorithm was found to have a positive predictive value of simultaneous ECG‐confirmed AF of 0.84.^395^ Only 0.5% of the enrolled population received any irregular pulse notification, but 3.2% of those age ≥65 years received notifications. However, only 153/450 (34%) patients had AF detected by a subsequent single ECG patches after the irregular rhythm notification was received. This may reflect the paroxysmal nature of early‐stage AF rather than explicit false positives. Because the study only administered ECG patch morning to those with irregular rhythm notification rather than then entire cohort or to negative controls, the negative predictive value was not estimated. It should be noticed that the Apple Heart Study was in a population without diagnosed AF; test performance and diagnostic yield could be considerably different in a population with known AF, and this software is not approved for use for AF surveillance in established AF.

#### 6.1.2 The Huwaei heart study

A similar study was performed using smart device‐based (Huawei fitness band or smartwatch) PPG technology.^396^ The algorithm had been validated with over 29 485 PPG signals before commencement of the trial. More than 246,000 people downloaded the PPG screening app, of which about 187 000 individuals monitored their pulse rhythm for 7 months. AF was found in 0.23% (slightly lower than Apple Heart, possibly due to a younger and healthier enrolled cohort). Validation was achieved in 87% (PPV >90%) compared to 34% in Apple Heart. The results indicated that this was a feasible frequent continuous monitoring approach for the screening and early detection of AF in a large population.

A significant observation was that clinical decision‐support tools provided enabled management decisions, for example, almost 80% high‐risk patients were anticoagulated. Subsequent enrollment into the mAFA II trial showed significantly reduced risk of rehospitalization and clinical adverse events.^397^ These trial results encourage incorporation of such technology effectively into the AF management pathways at multiple levels, that is, screening and detection of AF, as well as early interventions to reduce stroke and other AF‐related complications.

#### 6.1.3 Fitbit study

Another large‐scale virtual study to identify episodes of irregular heart rhythm suggestive of AF was announced by Fitbit in May 2020 (HRS 2020 7 May 2020).

### 6.2 Point of care

The next step beyond parameterizing safety could be to actionably guide therapy at the point of care (Figure 6). For example, patients could obtain ECGs before and after taking “pill‐in‐the‐pocket” antiarrhythmic drug therapy such as flecainide to confirm AF, ensure no QRS widening, and confirm restoration of sinus rhythm. A similar approach has been proposed for rhythm‐guided use of direct OACs in lower‐risk AF patients with infrequent episodes either spontaneously or as the result of a rhythm control intervention including drugs and ablation; a randomized trial is in development.^398^ The use of smartwatch‐guided rate control as a treatment strategy could also be tested, as this may provide a more personalized approach rather than prior randomized trials of lenient versus strict rate control that used population level rather than personalized heart rate treatment thresholds.^399^

### 6.3 Questions

#### 6.3.1 Generalizability

This is key to application of results from trials. mHealth is widely available and often simple to apply and wear.

- Older individuals and those with low health literacy may find technologies difficult to use (5.4.2 Digital Divide), and this may be compounded by disease state, for example, previous stroke.
- Cost and service plans associated with smartphones and smartwatches may preclude their use in lower socioeconomic populations who are already under‐represented in clinical trials and in many geographies.

Thus, patients who volunteer in mHealth studies in the USA are more likely to be a white/non‐Hispanic, more educated, and less likely to have disease.

#### 6.3.2 Adherence

mHealth‐based evaluation of clinical endpoints may be confounded if adherence is low, particularly if there are no secondary means of endpoint assessments.^400^ Virtual designs may be more susceptible to the loss of participant engagement. For example, if monitoring is completely reliant upon mobile health technology and there are no traditional measures or in‐person visits to assess arrhythmia, then significant missing data due to low‐adherence may become a major limitation that could imperil the validity and generalizability of the findings. For example, among the 2,161 of the 419,297 that received an irregular pulse notification in the Apple Heart Study, only 945 completed a subsequent protocoled first study visit. Of these 658 ambulatory ECG patches shipped, there were only 450 with returned and analyzable data^394^

Development of effective strategies to increase retention and maintain high engagement remains an unmet need and is an area ripe for more research.

#### 6.3.3 Outcomes

These are key to adoption and reimbursement. More specifically, the clinical and prognostic impact of new outcome measures based on mobile health technologies may not be clear.

This is important for AF. For example, how do changes in AF burden compare to reductions in time to symptomatic sustained AF? Should AF identified on near‐continuous smartwatch monitoring be considered equivalent to AF diagnosed at hospitalization or in clinic? There is a growing body of literature that the “dose” of AF burden matters for a variety of important clinical endpoints, including stroke, HF, and death (See Section 3.1.3).^33^, ^181^, ^182^, ^183^, ^184^ Does pill‐in‐the‐pocket DOAC treatment of PAF adequately cover the risk of stroke? Some measures remain less well studied, like the occurrence of irregularity with a wearable pulse‐based monitor system, particularly without ECG confirmation.

Since these mHealth prediagnostic or diagnostic tools may then be directly tied to initiation or termination of treatment, rigorous evaluation of clinical safety and efficacy will be required and, in some cases, warrant a combined drug‐device regulatory approval.

Despite these challenges, there is enormous potential for patients to use these technologies to self‐monitor their arrhythmia treatment and extend this to manage comorbidities (See Section 4). The process of data transparency and accessibility to the patient may improve the patient’s engagement with their overall care, even if the data are not directly actionable by the patient. The restrictions to clinic access during the SARS‐Cov‐2 pandemic have accelerated the adoption of mHealth solutions.^405^ ECGs for clinical trials were recorded by smart devices and assessed at virtual visits instead of routine in‐person evaluations. In some cases, the entire management of clinical trials went online.

REFERENCES SECTION 6201

Treskes
RW
, 
van der Velde
ET
, 
Barendse
R
, 
Bruining
N
. Mobile health in cardiology: A review of currently available medical apps and equipment for remote monitoring. Expert Review of Medical Devices. 2016;13:823–30.2747758410.1080/17434440.2016.1218277202

Feldman
DI
, 
Robison
TW
, 
Pacor
JM
, 
Caddell
LC
, 
Feldman
EB
, 
Deitz
RL
, …
Blaha
MJ
. Harnessing mHealth technologies to increase physical activity and prevent cardiovascular disease. Clinical Cardiology. 2018;41:985–991.2967187910.1002/clc.22968PMC6489886203

Varma
N
, 
Epstein
AE
, 
Irimpen
A
, 
Schweikert
R
, 
Love
C
, 
Investigators
TRUST
. Efficacy and safety of automatic remote monitoring for implantable cardioverter‐defibrilla‐tor follow‐up: The Lumos‐T Safely Reduces Routine Office Device Follow‐up (TRUST) trial. Circulation. 2010;2010(122):325–32. 10.1161/CIRCULATIONAHA.110.937409.20625110204

Heidbuchel
H
, 
Hindricks
G
, 
Broadhurst
P
, 
Van Erven
L
, 
Fernandez‐Lozano
I
, 
Rivero‐Ayerza
M
, …
Annemans
L
. EuroEco (European Health Economic Trial on Home Monitoring in ICD Patients): A provider perspective in five European countries on costs and net financial impact of follow‐up with or without remote monitoring. European Heart Journal. 2015;36:158–69. 10.1093/eurheartj/ehu339.25179766PMC4297469205

Crossley
GH
, 
Boyle
A
, 
Vitense
H
, 
Chang
Y
, 
Mead
RH
;CONNECT Investigators
. The CONNECT (Clinical Evaluation of Remote Notification to Reduce Time to Clinical Decision) trial: The value of wireless remote monitoring with automatic clinician alerts. Journal of the American College of Cardiology. 2011;57:1181–9.2125595510.1016/j.jacc.2010.12.012206

Guedon‐Moreau
L
, 
Lacroix
D
, 
Sadoul
N
, 
Clementy
J
, 
Kouakam
C
, 
Hermida
JS
, 
Aliot
E
, …
Kacet
S
. Costs of remote monitoring vs. ambulatory follow‐ups of implanted cardioverter defibrillators in the randomized ECOST study. Europace. 2014;16:1181–8. 10.1093/europace/euu012.24614572PMC4114330207

Hindricks
G
, 
Taborsky
M
, 
Glikson
M
, 
Heinrich
U
, 
Schumacher
B
, 
Katz
A
, …
Søgaard
P
;IN‐TIME Study Group
. Implant‐based multiparameter telemonitoring of patients with heart failure (IN‐TIME): A randomised controlled trial. Lancet. 2014;384:583–590.2513197710.1016/S0140-6736(14)61176-4208

Mabo
P
, 
Victor
F
, 
Bazin
P
, 
Ahres
S
, 
Babuty
D
, 
Da Costa
A
, …
Daubert
JC
. A randomized trial of long‐term remote monitoring of pacemaker recipients (the COMPAS trial). European Heart Journal. 2012;33:1105–11.2212741810.1093/eurheartj/ehr419PMC3341630209

Jiang
X
, 
Ming
WK
, 
You
JH
. The cost‐effectiveness of digital health interventions on the management of cardiovascular diseases: Systematic review. Journal of Medical Internet Research. 2019;21:e13166. 10.2196/13166.31210136PMC6601257210

Orchard
JJ
, 
Neubeck
L
, 
Freedman
B
, 
Webster
R
, 
Patel
A
, 
Gallagher
R
, …
Lowres
N
. Atrial Fibrillation Screen, Management and Guideline Recommended Therapy (AF SMART II) in the rural primary care setting: An implementation study protocol. British Medical Journal Open. 2018;8:e023130.10.1136/bmjopen-2018-023130PMC625275830385444211

Bruining
N
, 
Caiani
E
, 
Chronaki
C
, 
Guzik
P
, 
van der Velde
E
;Task Force of the e‐Cardiology Working Group
. Acquisition and analysis of cardiovascular signals on smartphones: potential, pitfalls and perspectives: By the Task Force of the e‐Cardiology Working Group of European Society of Cardiology. European Journal of Preventive Cardiology. 2014;21(2 Suppl):4–13.10.1177/204748731455260425354948212

Shuren
J
, 
Patel
B
, 
Gottlieb
S
. FDA regulation of mobile medical apps. Journal of the American Medical Association. 2018;320:337–338.2997133910.1001/jama.2018.8832213
Center for Devices, Radiological Health
. Digital Health. U.S. Food and Drug Administration; 2019. [Internet]. Available at: https://www.fda.gov/medical‐devices/digital‐health.214

Lee
TT
, 
Kesselheim
AS
. U.S. Food and Drug Administration precertification pilot program for digital health software: Weighing the benefits and risks. Annals of Internal Medicine, 2018;168:730–732.2963295310.7326/M17-2715215
PRE‐CERT
. Available at: https://www.fda.gov/medical‐devices/digital‐health‐soft‐ware‐precertification‐pre‐cert‐program/precertification‐pre‐cert‐pilot‐program‐milestones‐and‐next‐steps.216

Kagiyama
N
, 
Shrestha
S
, 
Farjo
PD
, 
Sengupta
PP
. Artificial intelligence: Practical primer for clinical research in cardiovascular disease. Journal of the American Heart Association. 2019;8:e012788.3145099110.1161/JAHA.119.012788PMC6755846217

Davenport
T
, 
Kalakota
R
. The potential for artificial intelligence in healthcare. Future Healthcare Journal. 2019;94–98.10.7861/futurehosp.6-2-94PMC661618131363513

## 7 OPERATIONAL CHALLENGES

### 7.1 Healthcare system—Ehealth monitoring and hospital ecosystem

#### 7.1.1 Transmission

A fundamental but as yet unresolved challenge of incorporating mHealth into clinical practice is the channel of data communication between patient and provider. This may differ depending upon whether the data are physician‐facing (e.g., for CIEDs) or patient‐facing (consumer digital health products, e.g., the Apple Watch; Apple Inc., Cupertino, CA).

##### CIEDs

Experience with CIEDs provides a framework. CIEDs generate voluminous quantities of eHealth data. In a single patient, this may be generated from distinct sources, that is, remote monitoring and in‐person interrogations. Transmission from remote monitoring has been well worked out: data flow from the CIED to the remote transceiver and then to the manufacturer’s server for access by individual practices. Unfortunately, this is usually retrieved in an image format rendering the granular data uninterpretable by the practice’s electronic health record (EHR). When shared with the patient, the image file is posted on the EHR’s patient portal. These files are difficult for physicians to interpret and practically uninterpretable by the lay public. In order to engage patients and caregivers, the data will need to be provided in a format that enables the lay public to get a high‐level summary of key features (such as battery status and remote monitor function status) with explanations and the ability to drill down to the more granular details for those individuals who wish to do so.

##### Consumer digital health product data

Consumers are rapidly adopting products to monitor their health status for early detection of abnormalities as well as for managing chronic diseases. These tools empower and engage patients in managing their health, but the very basic task of sharing the data with their healthcare provider presents challenges. From a technical standpoint, many EHR portals do not permit patients to send attachments. Therefore, the patient and provider are left using email, which is not considered secure or HIPPA or GDPR compliant. Even if the EHR portal accepts attachments, incorporating the digital health data into the EHR remains ad hoc and inconsistent. The logistical and practical concerns frighten many care providers into discouraging their patients from using these devices. Concerns among providers include the fear of being inundated with unnecessary transmissions to review as well as the concern that patients may send inappropriate data, for example, BP or glucose monitoring data to their electrophysiologist. Cloud‐based storage may avoid some of these challenges.

#### 7.1.2 Interoperability—Lack of organized infrastructure to receive incoming the data

Assimilating the data obtained from digital health tools, whether implantable or wearable, is proving to be one of the greatest clinical challenges. Clinicians feel increasingly burdened as both the volume of data as well as the sources of data increase. Creating the nomenclature and data models that would enable the information to be incorporated in the electronic medical record is less a technical challenge, but more a political challenge. It requires a consensus from the clinical community regarding definitions of the terminology and agreement on what data are required. For example, for pacemakers, there must be agreement on the definition of battery longevity, pacing thresholds, mode switch, etc. For CIEDs, this work has been done (https://www.iso.org/standard/63904.html,^406^)

The next step is for EHR vendors to support the agreed‐upon nomenclature and the data standard in which it is communicated. With these 2 building blocks, digital health data can be assimilated into the clinical workflow, enabling healthcare providers to review, manage, and document clinical impressions and recommendations within the environment of their EHR. This work is ongoing in the domain of CIEDs but has not started for wearable devices. It requires a coalition of clinicians, engineers, regulatory agencies as well as regulatory and/or financial incentives for vendors. A high‐efficient computerized system with huge storage is necessary infrastructure and may provide the platform for predictive analytics.

#### 7.1.3 Interoperability—Lack of organized infrastructure to transmit data and instructions

There is interest in mHealth to support patients with text messaging^407^ or mobile applications to remind patients of medication doses and times or medical appointments. To be effective, this requires synchronization with healthcare providers, ideally by integration with the EMR, allowing changes in medications and doses, as well as appointments, to flow between patients and clinicians in an accurate and bidirectional manner.^408^ However, EMR systems software is lacking such functionality and interoperability at this point.^409^

### 7.2 Cybersecurity guidance for mHealth devices

Interconnection of medical devices and clinical data promises facilitation of clinical care but also creates opportunities for intrusions by maleficent actors (i.e., hackers) to disable systems and/or access private health information (PHI).^190^, ^191^ The motivation is largely financial. Healthcare facilities and medical device companies present attractive targets because a number of attack strategies can yield large financial rewards:

1. Ransomware. A hospital’s systems can be locked out (e.g., data may be encrypted) until the attacker is paid^192^, ^193^
2. Theft and sale of patient data (i.e., PHI).
3. Company attack. A hacker may identify flaws in a system or device, short the company’s stock, and then make the flaws public. Alternatively, a maleficent user may try to harvest insider information from a breached company’s network. Attackers may compromise a company, but not take any of the above actions. Instead, they may sell their methods or credentials to another group who will use them^414^

Scenarios where a cyber attack results in the deaths of individuals or groups (e.g., by corrupting the firmware of a pacemaker or insulin pump) can be easily imagined and have been demonstrated by researchers,^415^ but to date, no such attack is known to have occurred in the real world. It is possible that that this is because attacks against organizations yield greater gain than attacks against individuals.

It is essential therefore to establish best practice methods to maintain patient safety and privacy in this new ecosystem of remotely managed devices and mass data collection.

#### 7.2.1 Hacking strategies and methods in mHealth technologies

Often times, attackers will not directly compromise the system that they are after; they will instead start by compromising a weaker link. For example, if the goal is to obtain PHI about a specific patient, they may attempt to get the patient (or a staff member) to install a malicious app, compromising the rest of the phone, including email and other credentials. From this point, the attacker is in a better position to attack the actual target. The process of chaining exploits to work through a system is called *pivoting*. Each pivot or “hop” enables new privileges that bring the hacker closer to desired goals.

The easiest thing to exploit is often a person with *phishing* campaigns. A compromised email account can be used to reset passwords for other services and to distribute more realistic phishing messages. More technical attack pathways are used to compromise the remote‐monitoring components of a healthcare system, for example, wireless links (bluetooth, wifi, etc.), Internet and local network communications or servers (databases, web frontends, file servers, etc.)

#### 7.2.2 Recommendations to the manufacturer

It is not possible to create systems that cannot be hacked. However, systems/devices should be designed to *fail gracefully* in conjunction with a plan. This enables rapid correction in the event of intrusion.

Business decisions (e.g., budget, timeline) should not override security which should be the priority. Attempting to close or obscure devices/protocols is not a solution, and the so called *security through obscurity*, as a defensive measure, has long been rejected as inadequate.^416^ A balance between usability and security has to be struck carefully. Securing devices against attackers, while keeping them open to clinicians is a difficult task. In mHealth, this difficulty can be amplified by the dependence on the patient’s devices (e.g., smartphone) and practices, which are outside the control of a healthcare IT system. An example of an engineering compromise in implantable cardiac devices is the requirement for important wireless communications to only work at very short ranges. These communications could be made more secure but less usable (e.g., requiring wires), or less secure but more usable (e.g., using Bluetooth).

#### 7.2.3 Recommendations to clinicians and administrators

The organization should be designed with *security in layers* (also called *defense in depth*), where each system is protected with more than one layer of security. Hence, a breach in one layer will not necessarily result in total compromise. For example, a database may (a) require a password, (b) only grant a minimum level of access to each user, and (c) only accept internal connections. Thus, if a user’s password is compromised (#1 failed), an attacker still cannot use it remotely. If the server is accidentally opened to remote access (#3 failed), the attacker can still only access that one user’s data. Other innovative solutions include delegating security to a personal base station to use a novel radio design that can act as a jammer‐cumreceiver.^417^

When recommending devices for patients, it is important to consider the potential privacy/security weaknesses compared to alternatives, ensure the patient is informed about these tradeoffs, and review how the manufacturer has responded to security incidents in the past.^418^ However, the lack of outcome data, combined with the lack of documented real‐world instances of actual cybersecurity intrusions to these devices or to peripheral products that support device connectivity (programmer, home communicator, database, communication protocols), pose a difficult risk–benefit assessment for clinicians and patients alike.

Regulatory frameworks around cybersecurity are changing rapidly.^419^ The FDA (as well as other regulatory agencies worldwide) now includes security as a part of device safety/efficacy checks, and we encourage readers to report security issues to manufacturers and the government (e.g., through FDA Medwatch).^420^

#### 7.2.4 Recommendations to patients

Clear advice to patients concerning cybersecurity should be followed by a formal patient informed consent.

### 7.3 Reimbursement

Reimbursement is a powerful driver of adoption of new clinical pathways and typically instituted once an intervention has been proven scientifically valid and cost‐effective.^421^ This process has only just started in mHealth and may be more complex to measure given the wide scope of telemedicine.

#### 7.3.1 Reduced costs

This technology may promote an effective means for early diagnosis and treatment of arrhythmias and associated comorbidities, leading to benefits of screening, prevention, and early treatment, thereby reducing adverse effects related to delayed therapy and utilization of costly healthcare resources (e.g., ER visits or hospitalizations). mHealth may help individuals adhere to health recommendations, empower active participation in lifestyle changes to modify cardiovascular risk profile, and promote adherence to medical therapy.^422^ Together, these may reduce the burden of chronic disease and associated long‐term disability. However, assessment of these longer‐term cost advantages is challenging, and value will vary according to country and healthcare system.

#### 7.3.2 Increased costs

Conversely, there are costs associated with administering mHealth programs. The widespread availability of smartphones and other commercially available mobile devices will generate a significant amount of inconclusive or false positive findings, which will in turn lead to additional testing for validation, thereby increasing utilization of healthcare resources. Widespread implementation of screening programs would require additional consideration of costs related to detection of arrhythmias in currently unscreened populations. Healthcare providers will also be required to spend time reviewing and interpreting potentially voluminous results (and associated phone calls) prior to making additional evaluation and management decisions. This requires financial compensation in order to maintain a viable practice.

#### 7.3.3 Remote monitoring of implanted devices

This provides valuable experience. RCTs conducted over many years that demonstrated safe and effective replacement of traditional in‐clinic evaluations, and more effective discovery of asymptomatic clinical events.^423^ Health‐economic studies like EuroEco (ICD patients) showed that clinic time needed for checking web‐based information, telephone contacts, and in‐clinic discussion when required was balanced by fewer planned in‐office visits with remote monitoring, resulting in a similar cost for hospitals vs. purely in‐office follow‐up.^424^ From a payer perspective, there was a trend for cost‐saving given fewer and shorter hospitalizations, seen also in other trials.^205^, ^206^, ^207^, ^208^ However, in systems with fee‐for‐service reimbursement, less in‐office visits (and hospitalizations) will lead to less income for the providers (i.e., physicians and hospitals) without adaption of the new remote‐monitoring paradigm. This illustrates the complexities in reimbursement.

Currently, remote‐monitoring reimbursement (e.g., USA, Germany, France, UK) is implemented in a discrete way following the protocols of randomized trials like TRUST or IN‐TIME,^427^ with billing after demonstration of a remote contact, with a maximum number per year. Given the technological trend toward more continuous transmissions, and decision‐support server systems that alert healthcare providers of potentially relevant information, possibly a subscription‐based system providing a lump sum per year per followed patient may be more effective. This should cover costs of hardware, software, and other services (like potential use of third‐party data monitoring centers) and would result in a much better prospective budgeting for both healthcare insurers and providers. This scheme may be apt for mobile technology.

It is anticipated that mobile health technology may provide a more efficient and cost‐effective approach to healthcare delivery that could improve clinical workflow and enhance clinical care when integrated into clinical practice.^429^ Linking this to improved outcome will be an important driver of reimbursement, for example, for a process leading to an arrhythmia management decision (but not when monitoring the large asymptomatic population without risk factors). Ongoing studies evaluating mobile technology, such as use of a smartphone ECG for AF screening in the AF SMART II (Atrial Fibrillation Screen, Management and Guideline Recommended Therapy) study, include a cost‐effectiveness analysis.^430^ Responsibilities for reimbursement may extend beyond traditional parties in health care and drive novel pathways. Mobile device companies are clearly interested in reimbursement issues, evidenced by contact between Apple health executives and insurance companies.^431^ Initiatives undertaken in the USA are described in Appendix 1.

### 7.4 Regulatory landscape for mHealth devices

The pace of changes and improvement of digital technology is furiously fast. With the release and spread of the 5G cellular technology, this growth will probably be strengthened, and new frontiers around data streaming and associated analytics will be crossed. Unfortunately, this growth has been slower in the field of digital technologies, particularly in the United States. The reasons are probably linked to the unique relationship between the government and its healthcare system. In the United States, mHealth technologies are primarily led by private organizations operating under constraints linked to financial incentives (CMS reimbursement guidelines), patient privacy (Health Insurance Portability and Accountability Act), and patient safety (Food and Drug Administration, FDA). These constraints have become obsolete with the development of the digital health technologies and novel mHealth devices, and a new regulatory paradigm is being formed.

The FDA released an entirely new section under the Medical Device category called “Digital Health” which is managed by the Center for Devices and Radiological Health (CDRH) ^212^, ^213^FDA.Gov. This development was triggered and supported by the 21st Century Cures Act signed into law on December 13, 2016. It is designed to help accelerate medical product development and bring new innovations and advances to patients. The FDA Digital Health policy is currently defined under three main categories: General Wellness, Mobile Medical Apps (MMAs), and Clinical Decision‐Support Systems. mHealth devices are present in these three categories which are defined as follows:

A wellness device is developed “for maintaining or encouraging a healthy lifestyle and is unrelated to the diagnosis, cure, mitigation, prevention, or treatment of a disease or condition” (21 CCA Section 3060 (a)(o)(1)(B)). The FDA‐regulated MMAs on the other hand as software that is focusing on traditionally regulated health functionalities and is categorized as software as a medical device (SaMD). The SaMD must be developed under well‐defined frame‐works involving specific software development life cycles (IEC‐62304), risk assessment, reliability demonstration, and safety that includes cybersecurity. The clinical decision‐support (CDS) systems may rely on mHeath devices, or be included in mHeath devices. The definitions of a CDS are provided in the 21 CCA, Section 520 (o)(1)(E). Briefly, they involve the presentation of medical data, recommendations to physicians about the prevention, diagnosis, or treatment of a condition or disease. It is not the intent that the healthcare professional primarily relies on this information to make a clinical diagnosis or treatment decisions. If wellness devices do not require FDA approval to be commercialized both SaMD and CDS do.

The regulatory policies are changing and adapting over time to fit the technology development of mHeath devices. Today, the time required for approving new technologies is significantly longer than the pace of change of the mHealth technologies. Hence, streamlining the regulatory submission process is of great interest to many stakeholders. One of the very recent initiatives in the USA designed to address this challenge is the FDA’s digital health software Precertification program.^214^, ^215^ The Pre‐CERT is developed to shift the current paradigm of SaMD submission. The program is ambitious and proposes to expedite regulatory review for the companies that can demonstrate a series of components that includes process certification, postmarket review, and real‐world evidence (among others). It is expected that a company gaining FDA Pre‐CERT could ultimately eliminate or streamline their regulatory submission process depending on the risk associated with their SaMD technologies. Started in 2019, this initiative currently involves international companies that are pushing their wellness technologies into the clinical realm. This type of new regulatory framework will certainly help corporate America to accelerate the commercialization of their products, but the Pre‐CERT might be much more difficult to reach by smaller companies that do not have the resources to demonstrate the level of trust, and to implement the level of verification and transparency Pre‐CERT requires.

REFERENCES SECTION 7218

Marcolino
MS
, 
Oliveira
JAQ
, 
D'Agostino
M
, 
Ribeiro
AL
, 
Alkmim
MBM
, 
Novillo‐Ortiz
D
. The impact of mhealth interventions: Systematic review of systematic reviews. Journal of Medical Internet Research Mhealth Uhealth. 2018;6:e23.10.2196/mhealth.8873PMC579269729343463219

Steinhubl
SR
, 
Muse
ED
, 
Topol
EJ
. The emerging field of mobile health. Science Translational Medicine. 2015;7:283rv3.10.1126/scitranslmed.aaa3487PMC474883825877894220

Pogue
D
. Yahoo! Finance. Exclusive: What Fitbit's 6 billion nights of sleep data revealsabout us. 2020. Available at: https://finance.yahoo.com/news/exclusive‐fitbits‐6‐billion‐nights‐sleep‐data‐reveals‐us‐110058417.html.221

Bumgarner
JM
, 
Lambert
CT
, 
Hussein
AA
, 
Cantillon
DJ
, 
Baranowski
B
, 
Wolski
K
, …
Tarakji
KG
. Smartwatch algorithm for automated detection of atrial fibrillation. Journal of the American College of Cardiology. 2018;71:2381–2388.2953506510.1016/j.jacc.2018.03.003222

Nascimento
BR
, 
Beaton
AZ
, 
Nunes
MCP
, 
Tompsett
AR
, 
Oliveira
KKB
, 
Diamantino
AC
, …
Sable
C
. Integration of echocardiographic screening by non‐physicians with remote reading in primary care. Heart. 2018;105:283–290. 10.1136/heartjnl-2018-313593.30181202223

Ribeiro
ALP
, 
Paixão
GMM
, 
Gomes
PR
, 
Ribeiro
MH
, 
Ribeiro
AH
, 
Canazart
JA
, …
Macfarlane
PW
. Teleelectrocardiography and bigdata: The CODE (Clinical Outcomes in Digital Electrocardiography) study. Journal of Electrocardiology. 2019;57S:S75–S78. 10.1016/j.jelectrocard.2019.09.008.31526573224

Seetharam
K
, 
Kagiyama
N
, 
Sengupta
PP
. Application of mobile health, telemedicine and artificial intelligence to echocardiography. Echo Research and Practice. 2019;6:R41–R52.3084475610.1530/ERP-18-0081PMC6432977225

Hannun
AY
, 
Rajpurkar
P
, 
Haghpanahi
M
, 
Tison
GH
, 
Bourn
C
, 
Turakhia
MP
, 
Ng
AY
. Cardiologist‐level arrhythmia detection and classification in ambulatory electrocardiograms using a deep neural network. Nature Medicine. 2019;25:65–69.10.1038/s41591-018-0268-3PMC678483930617320226

Ribeiro
AH
, 
Ribeiro
MH
, 
Paixão
GMM
, 
Oliveira
DM
, 
Gomes
PR
, 
Canazart
JA
, 
Ribeiro
ALP
.Automatic diagnosis of the short‐duration 12‐lead ECG using a deep neural network: TheCODE study [Internet]. 2019. arXiv [cs.LG]. http://arxiv.org/abs/1904.01949
227

Smith
SW
, 
Walsh
B
, 
Grauer
K
, 
Wang
K
, 
Rapin
J
, 
Li
J
, 
Fennell
W
, 
Taboulet
P
. A deep neural network learning algorithm outperforms a conventional algorithm for emergency department electrocardiogram interpretation. Journal of Electrocardiology. 2019;52:88–95.3047664810.1016/j.jelectrocard.2018.11.013228

Zhang
J
, 
Gajjala
S
, 
Agrawal
P
, 
Tison
GH
, 
Hallock
LA
, 
Beussink‐Nelson
L
, …
Deo
RC
. Fully automated echocardiogram interpretation in clinical practice. Circulation. 2018;138:1623–1635.3035445910.1161/CIRCULATIONAHA.118.034338PMC6200386229

Attia
ZI
, 
Kapa
S
, 
Lopez‐Jimenez
F
, 
McKie
PM
, 
Ladewig
DJ
, 
Satam
G
, …
Friedman
PA
. Screening for cardiac contractile dysfunction using an artificial intelligence‐enabled electrocardiogram. Nature Medicine. 2019a;25:70–74.10.1038/s41591-018-0240-230617318230

Attia
ZI
, 
Noseworthy
PA
, 
Lopez‐Jimenez
F
, 
Asirvatham
SJ
, 
Deshmukh
AJ
, 
Gersh
BJ
, …
Friedman
PA
. An artificial intelligence‐enabled ECG algorithm for the identification of patients with atrial fibrillation during sinus rhythm: A retrospective analysis of outcome prediction. Lancet. 2019b;394:861–867.3137839210.1016/S0140-6736(19)31721-0231

Halcox
JPJ
, 
Wareham
K
, 
Cardew
A
, 
Gilmore
M
, 
Barry
JP
, 
Phillips
C
, 
Gravenor
MB
. Assessment of remote heart rhythm sampling using the alivecor heart monitor to screen for atrial fibrillation: The REHEARSE‐AF study. Circulation. 2017;136:1784–1794.2885172910.1161/CIRCULATIONAHA.117.030583232

Chamsi‐Pasha
MA
, 
Sengupta
PP
, 
Zoghbi
WA
. Handheld echocardiography: Current state and future perspectives. Circulation. 2017;136:2178–2188.2918049510.1161/CIRCULATIONAHA.117.026622233

Awan
SE
, 
Bennamoun
M
, 
Sohel
F
, 
Sanfilippo
FM
, 
Dwivedi
G
. Machine learning‐based prediction of heart failure readmission or death: Implications of choosing the right model and the right metrics. European Society of Cardiology Heart Failure Journal. 2019;6:428–435.10.1002/ehf2.12419PMC643744330810291

## 8 PREDICTIVE ANALYTICS

Artificial Intelligence (AI) is a broad term that describes any computational programs that normally require human intelligence such as image perception, pattern recognition, inference, or prediction (www.oed.com).^436^ Most commonly, AI is implemented using analytical methods of machine learning or deep learning. These methods are well suited for pattern classifications, such as images, including ECG.

The potential synergy between AI and mHealth has excited the healthcare community since this may enable solutions to improve patient outcomes and increase efficiency with reduced costs in health care.^217^, ^218^ Smartphone apps and wearable devices generate a huge amount of data that exceed the human capacity of integration and interpretation.^439^ Biometric datasets of astronomical proportions may be compiled. This knowledge may be directed to treat an invidual, or understand populations. For instance, 6 billion nights of surrogate sleep data reflecting global sleep deprivation may potentially inform public health initiatives (https://aasmorg/fitbit‐scientists‐reveal‐results‐analysis‐6‐billion‐nights‐sleep‐data).^440^ Mobile health with Internet connection enables cloud‐based predictive analytics from individual‐level information.^221^, ^222^, ^223^

Cardiology has been an early area of investigation in AI due to the abundance of data well suited for classification and prediction.^444^ Neural networks have been tested, trained, and successfully validated to be at least as accurate, if not more, than physicians in diagnosis or classification of 12‐lead ECGs and recognition of arrhythmias in rhythm strips and ambulatory ECG recordings.^225^, ^226^, ^227^ They have also been shown to successfully estimate ejection fraction, identify left ventricular dysfunction, and even diagnosis diagnose diseases such as hypertrophic cardiomyopathy from the echocardiogram.^448^ More recently, neural networks have also aided in gathering new dimensions of information, such as identifying left ventricular dysfunction.^449^ These methods have the potential for a point‐of‐use diagnosis of a wearable sensor or consumer device and without delays of requiring clinical conformation, although rigorous safety assessments of unsupervised use will be necessary. More recently, AI methods have also been applied to prediction, not just classification, for example, using 12‐lead ECG to predict risk of AF from a sinus rhythm ECG.^450^

Already, AI has been embedded in mHealth applications, such as smartwatch and smartphone‐connect ECG semi‐automated diagnosis of arrhythmias.^221^, ^231^ These diagnoses are intended to serve as prediagnostics rather than supplanting a physician interpretation. Application of artificial intelligence techniques to point‐of‐care ultrasound in the development of machine‐learning systems may aid in the optimization of acquisition and interpretation of a high volume of images, reduce variability, and improve diagnostic accuracy.^452^ AI‐based prediction models have been developed for HF and AF, although sometimes the accuracy of the AI‐derived models seems to be rather limited or not superior than those derived from conventional methods.^233^, ^234^, ^235^, ^236^, ^237^, ^238^ mHealth specific investigations are few. Results from the LINK‐HF study were encouraging. A cloud‐based analytics platform used a general machine‐learning method of similarity‐based modeling which models the behavior of complex systems (e.g., aircraft engines) to create a predictive algorithm for HF decompensation, using data streamed from a chest patch sensor.

Several limitations should be considered and roadblocks removed before AI‐based mHealth strategies become routinely incorporated in clinical practice.^216^, ^219^, ^224^, ^239^ Studies on AI are still scarce and based on observational studies and secondary datasets. Validation in other clinical settings and a deeper evaluation of their meaning in every day practice are generally lacking. Thus, high‐quality evidence that supports the adoption of many new technologies is not available. Most algorithms work with the "black box" principle, without allowing the user to know the reasons why a diagnosis or recommendation was generated, which can be a problem, especially if the algorithms were designed for a different environment than the one that the current patient is inserted^239^, ^240^ Issues regarding cost‐effectiveness, implementation, ethics, privacy, and safety are still unsolved.

REFERENCES SECTION 8234

Clifton
DA
, 
Niehaus
KE
, 
Charlton
P
, 
Colopy
GW
. Health informatics via machine learning for the clinical management of patients. Yearbook of Medical Informatics. 2015;10:38–43.2629384910.15265/IY-2015-014PMC4587065235

Frizzell
JD
, 
Liang
L
, 
Schulte
PJ
, 
Yancy
CW
, 
Heidenreich
PA
, 
Hernandez
AF
, …
Laskey
WK
. Prediction of 30‐day all‐cause readmissions in patients hospitalized for heart failure: Comparison of machine learning and other statistical approaches. Journal of the American Medical Association Cardiology. 2017;2:204–209.2778404710.1001/jamacardio.2016.3956236

Goto
S
, 
Goto
S
, 
Pieper
KS
, 
Bassand
JP
, 
Camm
AJ
, 
Fitzmaurice
DA
, 
Kakkar
AK
. New AI prediction model using serial PT‐INR measurements in AF patients on VKAs: GARFIELD‐AF. European Heart Journal Cardiovasc Pharmacother. 2019. 10.1093/ehjcvp/pvz076.PMC755681131821482237

Safavi
KC
, 
Khaniyev
T
, 
Copenhaver
M
, 
Seelen
M
, 
Zenteno Langle
AC
, 
Zanger
J
, …
Dunn
P
. Development and validation of a machine learning model to aid discharge processes for inpatient surgical care. Journal of the American Medical Association Network Open. 2019;2:e1917221.10.1001/jamanetworkopen.2019.17221PMC699119531825503238

Tripoliti
EE
, 
Karanasiou
GS
, 
Kalatzis
FG
, 
Bechlioulis
A
, 
Goletsis
Y
, 
Naka
K
, …
Fotiadis
DI
. A knowledge management system targeting the management of patients with heart failure. Journal of Biomedical Informatics. 2019;94:103203.3107145510.1016/j.jbi.2019.103203239

Ribeiro
AL
, 
Oliveira
GMM
. Toward a patient‐centered, data‐driven cardiology. Arquivos Brasileiros de Cardiologia. 2019;112:371–373.3099471410.5935/abc.20190069PMC6459428240

Weng
SF
, 
Reps
J
, 
Kai
J
, 
Garibaldi
JM
, 
Qureshi
N
. Can machine‐learning improve cardiovascular risk prediction using routine clinical data?
PLoS One. 2017;12:e0174944.2837609310.1371/journal.pone.0174944PMC5380334241

Curry
SJ
, 
Krist
AH
, 
Owens
DK
, 
Barry
MJ
, 
Caughey
AB
, 
Davidson
KW
, …
Wong
JB
. US Preventive Services Task Torce, screening for atrial fibrillation with electrocardiography: US preventive services task force recommendation statement. Journal of the American Medical Association. 2018;320:478–484.3008801610.1001/jama.2018.10321242

Pluymaekers
NAHA
, 
Hermans
ANL
, 
van der Velden
RM
, 
den Uijl
DW
, 
Vorstermans
B
, 
Buskes
S
, …
Linz
D
. On‐demand app‐based rate and rhythm monitoring to manage atrial fibrillation through tele‐consultations during COVID‐19. International Journal of Cardiology Heart & Vascular. 2020;28:100533. 10.1016/j.ijcha.2020.100533.PMC720562632391412243

Turakhia
MP
. Diagnosing with a camera from a distance — Proceed cautiously and responsibly. JAMA Cardiology. 2020;5(1):107. 10.1001/jamacardio.2019.4572.31774448244

Yan
BP
, 
Lai
WHS
, 
Chan
CKY
, 
Au
ACK
, 
Freedman
B
, 
Poh
YC
, 
Poh
MZ
. High‐throughput, contact‐free detection of atrial fibrillation from video with deep learning. JAMA Cardiol. 2020;5(1):105–107. 10.1001/jamacardio.2019.4004.31774461PMC6902123245

Rosier
A
, 
Mabo
P
, 
Temal
L
, 
Van Hille
P
, 
Dameron
O
, 
Deléger
L
, …
Burgun
A
. Personalized and automated remote monitoring of atrial fibrillation. Europace. 2016;18:347–52. 10.1093/europace/euv234.26487670246

Turakhia
MP
. Diagnosing with a camera from a distance—Proceed cautiously and responsibly. Journal of the American Medical Association Cardiol. 2020;5:107.10.1001/jamacardio.2019.457231774448

## 9 FUTURE DIRECTIONS

**FIGURE 7:**
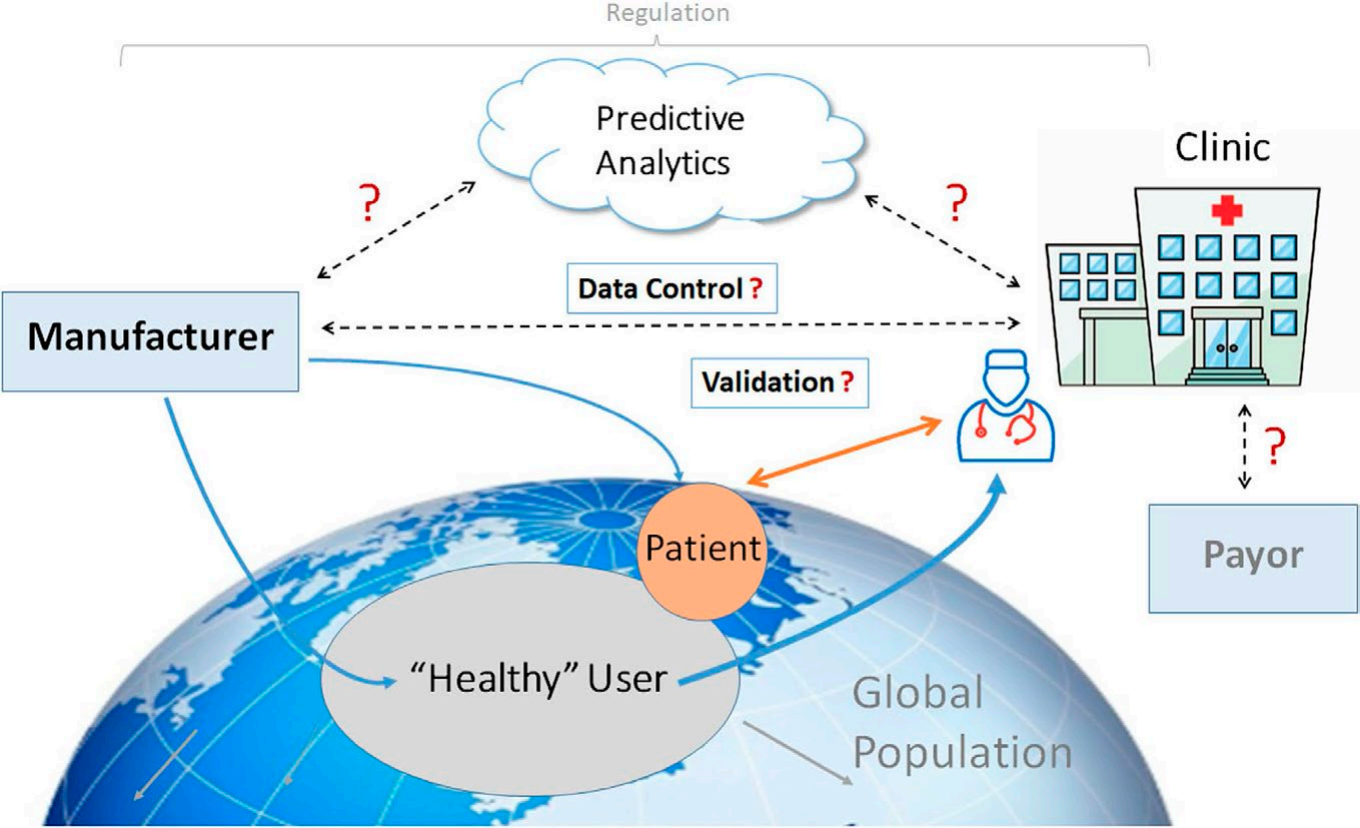
Connectivity and Questions. Multiple levels of cooperation among a variety of stakeholders are needed to capitalize fully on the vast potential of mHealth, but many questions remain unanswered. Healthy consumers (increasing) predominate among mhealth users. Only a minority of patients are prescribed these digital tools. Potential health benefits of mHealth may be realized when manufacturer participates with clinic for validation in defined disease states. Parties responsible for data control—and therby predictive analytics—need to be defined. Ultimately, the payor and physician need to be convinced of benefits before digital tools are firmly embedded in clinical practice.

mHealth is disruptive at multiple levels of health care but requires significant investment in validation, demonstration of clinical utility and value. Stakeholders, each with independent concerns and constraints (Table 5) lack consensus or coordination with design, use cases, and implementation (Figure 7). Thus, formal recommendations for integration of mHealth into clinical practice cannot be made at this time. This is exemplified by the US Preventative Services Task Forces statement that “*evidence is insufficient* to initiate therapy for AF detected by mHealth”—despite the fact that AF has been an early use case with strong patient and clinician interest.^461^ Thus, mHealth devices are currently nonprescription devices marketed directly to consumers to track data without enabling interventions.

**TABLE 5:** Conditions, stakeholders and expectations

	Applications/Conditions	Opportunities	Challenges to resolve
Bio‐signals monitored	Diverse	Multiparametric trending Contactless screening	Lack of validation Transmission frequency Ethics
Target condition	Arrhythmias Treatment Follow‐upRehabilitation Lifestyle modification Chronic disease	Screening Prevention Facilitate management	Lack of outcome data
Users	Healthy consumers	Increase use by patients	Managing the “worried well”
Patient expectations	Confidence Engagement Education	Data access Real‐time treatment Self‐management	Data access Driven by popular press Excessive focus on data without clinical context Digital divide Lack of Internet access
Physician expectations	Versatility	Validation Improve patient outcome Reduce in‐clinic visits Real‐time patient treatment Predictive Analytics Precision Medicine	Absence of FDA approval Lack of outcome data Establish transmission frequency Define clinical actionability Manage false positives Standardize data flowManage data overload Interoperability with EMR Mechanism for feedback to patients for treatment decisions; Assurance of patient adherence Physician or Manufacturer? Reimbursement Legal responsibility
Hospital	Improve efficiencies Improve access	Predictive analytics Interoperability Cybersecurity Reimbursement	Lack of outcome data Value impact Legal responsibility
Technology/Manufacturer	Direct to Consumer Sales	Patient care Community care	Learn treatment pathways Partner with clinic Legal responsibility Predictive analytics
Payor	Reduce costs Improve outcome	Cost–benefit analysis	

Some of the steps needed to standardize mHealth applications are outlined below.

### 9.1 Validation

- Promote standards and create tools for the comparative assessment of functionality, relative to a medical use device.

Results from different devices applied to the same condition may not match: for example, the diagnosis of AF by ECG or PPG based systems are made very differently. This has significant implications for medical decisions.

### 9.2 Identify clinical care pathways

- Screening
  - Assess value according to the population addressed
  - Establish a uniform set of criteria for clinical actionability

Screening should be medically directed and not driven by commercial interests. Caution should be exercised in extrapolating management strategies learned from cohorts with clinically diagnosed AF (usually from healthcare system data, trials or inpatient registries) to AF detected with mHealth technologies (“healthy consumers”). Data from low‐risk populations carry a relatively high risk of false positives, which may generate additional tests with resultant clinical risk to patient (even inducing anxiety rather than reassurance), risk from overtreatment, and costs to the payor. There is a risk that unless directed to a higher risk population, screening for AF using mHealth technologies may fail and follow the trajectory of many medical screening programs throughout history.

*Key knowledge gap*—Identify characteristics (duration, episode number/density) and risk factors that justify anticoagulation for mHealth detected AF.

- Disease management
  - Identify conditions and schedules for home‐based therapeutic strategies that may reduce dependency on clinic evaluations (as shown for CIEDs)
  - Identify signals that predict decompensation and design pre‐emptive interventions
  - Assess efficacy of therapies.
- Outcomes

Evidence for benefit of mHealth directed:

- Arrhythmia treatment
- Management of modulating factors (e.g., comorbidities, lifestyle modifications).

### 9.3 Implementation

- Cost‐effectiveness

Eg impact of improved clinical workflow and enhance clinical care, according to condition.^429^

Impact on healthcare system and reimbursement.

Impact on costs to patient or consumer.

- Public health and professional society initiatives

Education, awareness

Bring together stakeholders

Guidelines.

### 9.4 Patient self‐management

Patients control the intensity of monitoring and act on patient‐facing data. Frequency of data acquisition is sporadic determined by, for example, convenience, or following symptoms, or recreational. This strategy is likely insensitive for events and rarely delivers rapid clinical actionability for life‐threatening conditions. What is required is as follows:

- Education on which data are clinically actionable in individual’s clinical context and
- Tailor monitoring schedule accordingly
- Proof of safety.

In one recent example illustrates an on‐demand use. The Fibricheck app was utilized by patients to monitor rate and rhythm for a week prior to teleconsultations during the COVID‐19 pandemic to enable remote assessment of the disease state and support treatment decisions. This was regulated by a time‐limited prescription to use the app for a predefined period, avoiding unnecessary data‐load and additional follow‐up patients‐contacts.^462^

- patients' legal right to their medical data to include data collected from nonmedical (i.e., consumer) products.

### 9.5 Manufacturer

mHealth introduces the manufacturer as a party with significant responsibilities. mHealth tools largely have been developed as consumer‐facing technologies accessible to a broader market through retail channels rather than through established medical supply channels. This may make business sense for the technology supplier, given high community penetration of wearable, smart‐technology devices (1 in 10 Americans (30 million total)). However, a direct to consumer healthcare delivery bypasses both the clinician, healthcare system, and insurer, without addressing the needs of health professionals—who remain responsible for clinical decision‐making on acquired data. Any advance toward medical application (beyond toys for the worried well/wealthy well) will require manufacturers to:

- Facilitate accessibility and affordability
- Engage with clinicians to engineer devices according to clinical needs and partner in validation. This is vital, since physician carries ultimate responsibility for medical decisions and is best positioned to guide development and application
- Define role as data controllers (e.g., GDPR in Europe).

### 9.6 Assign responsibilities

- Identify parties (manufacturer, hospital, third party) responsible for cybersecurity, data protection, and liability for misdiagnosis or missed diagnosis
- Define standard of care for clinic response time according to condition.

This assumes greater significance as clinical decisions become enabled in real time using cloud processing resources linked to enhanced data transmission rates (5G) and Internet of Things (IoT) and scalability increases.

- Ethical and societal issues with multiple screening.^243^, ^244^

### 9.7 Healthcare delivery

Interconnectedness between individual applications and with existing healthcare architectures may reshape the current environment.

- “Exception‐based” ambulatory care, that is, see patients as they need to be seen
- Centralized (cloud) based processing to forward only clinically relevant data to physician/clinic.
- Identify at‐risk patients early (even before symptoms develop) and permit pre‐emptive care.^465^
- Pooled population screening—altering the paradigm of individual screening.^244^, ^246^
- Extend the role of wearables from ambulatory to in‐hospital care, for example, replace traditional wired monitoring of single parameters for individual analysis, to wireless monitoring of multiple parameters.

For example, a waterproof ring technology (Bodimetrics) was used for multiparametric monitoring (heart rate, sleep, oxygen desaturation index, steps, and calories burned) in ICU management for COVID 19 patients. The ring links to a smartphone or centralized hub in hospitals and permits data sharing and cooperative treatment (https://bodimetrics.com/product/circul‐sleep‐and‐fitness‐ring/).

- Extend function from monitoring only to intervention
- Enable remote programing of therapeutic implantable devices.

For example, CIEDs, emerging wearable cardioverter‐defibrillators, are incorporating smartphone Bluetooth® Low Energy (BLE) based connectivity for the transmission, display, and interpretation of transmitted data by patients and their clinicians. This may permit reprograming of parameters like diagnostic data, detection zones, clearing counters, AV delays/PVARP adjustment, upper rate and lower rate adjustments, reprogram amplitude adjustments; MRI mode, and enable emergency therapies or disable inappropriate therapies due to lead fracture/incessant SVT/double counting.

- Enable interventional procedures, for example, Tele‐Robotic ablations models which could improve access to patients living in remote areas with highly skilled EPs operating remotely.^1^, ^2^, ^3^
- Enable precision medicine by incorporation of the wider range of mobile signals seamlessly into genetic and clinical profile, with environmental and lifestyle data (“big data”) (https://ghr.nlm.nih.gov/primer/precisionmedicine/initiative).

## CONCLUDING REMARKS

mHealth application is at different stages of evolution around the world. Few of the technologies described are universally approved and/or affordable in all countries. As a result, this document reflects largely US perspectives. The experience described may serve to guide other members of the international professional bodies endorsing this collaborative statement. The World Health Organization envisioned that increasing the capacity to implement and scale up cost‐effective innovative digital health could play a major role in toward achieving universal health coverage and ensuring access to quality health services, at the same time recognizing barriers to implementation similar to those discussed in this document. Some of these can be resolved rapidly, as seen in response to the recent SARS‐CoV‐2 global pandemic that forced a need for contactless monitoring and thereby adoption of digital tools (DHSS, FDA).^4^, ^5^, ^6^ Regulatory bodies were responsive, approving technologies, relaxing rules confining use of telehealth services within borders and to certain patient populations, and creating a reimbursement structure, illustrating that appropriate solutions can be created when necessary.

Demonstration of the clinical utility of mHealth has the potential to revolutionize how populations interact with health services, worldwide.

REFERENCES SECTION 91

Choi
PJ
, 
Oskouian
RJ
, 
Tubbs
RS
. Telesurgery: Past, present, and future. Cureus Journal of Medical Science. 2018;10:e2716. 10.7759/cureus.2716.PMC6067812300792822

Haidegger
T
, 
Sándor
J
, 
Benyó
Z
. Surgery in space: The future of robotic telesurgery. Surgical Endoscopy. 2011;25:681–690. 10.1007/s00464-010-1243-3.206523203

Shinoda
Y
, 
Sato
A
, 
Adach
T
, 
Nishi
I
, 
Nogami
A
, 
Aonuma
K
, 
Ieda
M
. Early clinical experience of radiofrequency catheter ablation using an audiovisual telesupport system. Heart Rhythm. 2020;17(5):870–875. 10.1016/j.hrthm.2020.01.018.323544524

Varma
N
, 
Marrouche
NF
, 
Aguinaga
L
, 
Albert
CM
, 
Arbelo
E
, 
Choi
JI
, 
Varosy
PD
. HRS/EHRA/APHRS/LAHRS/ACC/AHA worldwide practical guidance for telehealth and arrhythmia monitoring during and after a pandemic. Journal of the American College of Cardiology. 2020;76:1363–1374. 10.1016/j.jacc.2020.06.019.32534936PMC72890885
U.S. Department of Health and Human Services
.Notification of enforcement discretion for telehealth remote communications during the COVID‐19 nationwide public healthemergency. 2020. Available at: https://www.hhs.gov/hipaa/for‐professionals/special‐topics/emergency‐preparedness/notification‐enforcement‐discretion‐telehealth/index.html.6
U.S. Food Drug Administration
.Enforcement policy for non‐invasive remote monitoring devices used to support patient monitoring during the coronavirus disease‐2019(COVID‐19) public health emergency. 2020. Available at: https://www.fda.gov/regulatory‐information/search‐fda‐guidance‐documents/enforcement‐policy‐non‐invasive‐remote‐monitoring‐devices‐used‐support‐patient‐monitoring‐during.

## Supporting information

Supplement

## APPENDIX 1

In the United States, reimbursement for medical services is guided primarily by the Centers for Medicare & Medicaid Services (CMS). The American Medical Association’s Current Procedural Terminology (CPT) Committee develops descriptive codes for each medical service and assigns a CPT code. Each CPT code is then referred to the association’s Relative Value Update Committee to develop a recommended relative value unit (RVU) which determines reimbursement. CMS usually accepts the recommendations from the AMA. Presently, the CPT Committee is developing codes to represent the clinical work involved in managing mHealth data. These codes will then be evaluated and assigned RVU values. If accepted by CMS, these will be included in the Medicare Fee Schedule and go into clinical use. This process typically takes 2 years. Once the codes and services are approved by CMS and published in the Fee Schedule, other insurers typically accept them as well (at the time of writing CPT 99091 and CPT 99457 had received approval).

In 2015, the CMS in the USA initiated a new chronic care management code that reimburses primary care practices for nonface‐to‐face care for chronic care management (CCM) payment. In November 2018, CMS finalized plans to reimburse healthcare providers for certain remote patient monitoring and telehealth services. These changes focused on three new CPT codes that separate remote patient management (RPM) services from telehealth services. The new CPT codes include #99453, 99454, and 99457. The first two codes describe remote monitoring of physiologic parameters, but do not specifically include ECG monitoring. The third code provides management services, 20 minutes or more of clinical staff/physician/other qualified HCP time in a calendar month requiring interactive communication with the patient/caregiver during the month; however, it is not clear that this code could be utilized for ECG monitoring services through mobile devices. The pre‐existing CPT code 93040 (used for reporting on a Rhythm ECG, 1‐3 leads, without interpretation and report) would not be appropriate for patient initiated mobile device events as this would require an order that is triggered by an event followed by a separate signed and retrievable report. CMS has also proposed establishing a new virtual service HCPCS code, GRAS1, for “Remote Evaluation of Pre‐Recorded Patient Information,” which would reimburse for a provider’s asynchronous review of “recorded video and/or images captured by a patient in order to evaluate the patient’s condition” and determine whether or not an office visit is necessary. This code could be billed separately if there was not an E/M visit within the previous seven days. CMS finalized separate payment for CPT code 99091 (collection and interpretation of physiologic data, e.g., ECG, BP, glucose monitoring) digitally stored and/or transmitted by the patient and/or caregiver to the physician or other qualified HCP, qualified by education, training, licensure/regulation, requiring a minimum of 30 minutes of time. However, there must be a clinically relevant reason for the physician to need to review the data each month.
